# Prognostic Value of Regnase-1 in High-Grade Soft Tissue Sarcoma: Favourable in UPS, Yet Inverted in Adjuvantly Irradiated Patients

**DOI:** 10.3390/cancers18091419

**Published:** 2026-04-29

**Authors:** Julie Zangarini, Axel Künstner, Florian Lenz, Lars Tharun, Jan Vorwerk, Niklas Gebauer, Jutta Kirfel, Hauke Busch, Bruno Christian Köhler, Eva Wardelmann, Dirk Rades, Anastassia Löser, Nikolas von Bubnoff, Cyrus Khandanpour, Maxim Kebenko

**Affiliations:** 1 Clinic for Hematology and Oncology, University Hospital Schleswig-Holstein Campus Lübeck, 23562 Lübeck, Germany; juliezangarini@gmail.com (J.Z.); niklas.gebauer@uksh.de (N.G.); brunochristian.koehler@uksh.de (B.C.K.); nikolaschristiancornelius.vonbubnoff@uksh.de (N.v.B.); cyrus.khandanpour@uksh.de (C.K.); 2 The Research Group for Systems Biology, Institute for Experimental Dermatology (LIED), University Hospital Schleswig-Holstein Campus Lübeck, 23562 Lübeck, Germany; axel.kuenstner@uksh.de (A.K.); hauke.busch@uni-luebeck.de (H.B.); 3 Institute of Pathology, University Hospital Schleswig-Holstein Campus Lübeck, 23562 Lübeck, Germany; florian.lenz@uksh.de (F.L.); l.tharun@hanse-histologikum.de (L.T.); jutta.kirfel@uksh.de (J.K.); 4 Gerhard Domagk Institute of Pathology, University Hospital Münster (UKM), 48149 Münster, Germany; eva.wardelmann@ukmuenster.de; 5 Department of Radiation Oncology, University Hospital Schleswig-Holstein Campus Lübeck, 23562 Lübeck, Germany; dirk.rades@uksh.de (D.R.); anastassia.loeser@uksh.de (A.L.)

**Keywords:** high-grade STS, Regnase-1, ICI, TILs, CD68^+^ TAMs, PD-1, PD-L1, TIGIT

## Abstract

Background: High-grade soft tissue sarcomas (STSs) are heterogeneous tumours lacking robust prognostic or predictive biomarkers. Regnase-1, an immune RNase, enhances antitumour immunity by limiting immunosuppressive tumour microenvironment (TME) components (e.g., myeloid-derived suppressor cells (MDSCs)), but remains unexplored in STS. As CD68^+^ tumour-associated macrophages (TAMs) drive TME suppression and poor prognosis in non-translocation-driven STS, we evaluated Regnase-1 and CD68^+^ TAMs to assess Regnase-1 as an indicator of an immunologically activated TME. Methods: Immunohistochemistry scoring of Regnase-1 and CD68^+^ TAMs was performed in 91 patients. Overall survival (OS) was assessed by Kaplan–Meier and Cox regression, and findings were validated in an independent “The Cancer Genome Atlas” Sarcoma (TCGA-SARC) cohort (*n* = 212). Results: In UPS, Regnase-1-high predicted longer OS (17.0 months vs. not reached; *p* = 0.0247) and lower mortality (univariate hazard ratio (HR) = 0.3; *p* = 0.0343; multivariate HR = 0.4; *p* = 0.0413), but not after radiotherapy. CD68^+^ TAM-high predicted shorter OS (13.0 months vs. not reached; *p* = 0.0274) and higher mortality (HR = 2.0, 95% CI 1.1–3.7; *p* = 0.0325). Both Regnase-1 effects were reproduced in TCGA-SARC. Regnase-1-high tumours showed inflammatory/interferon enrichment, reduced TGF-β signalling, and SERPINE1 upregulation. Conclusions: Regnase-1 marked a pro-inflammatory TME and favourable outcome in UPS, but this effect may reverse upon radiotherapy.

## Simple Summary

High-grade soft tissue sarcomas are rare and aggressive cancers with limited biomarkers to guide prognosis and treatment. We investigated Regnase-1, a protein involved in immune regulation within the tumour environment. High Regnase-1 levels were associated with improved survival in a specific sarcoma subtype, suggesting a more active anti-tumour immune response. However, this effect was not observed in patients receiving radiotherapy, where the association appeared reversed. These findings indicate that the prognostic value of Regnase-1 depends on tumour type and treatment context. Understanding these differences may help to improve risk stratification and support more personalised treatment approaches in soft tissue sarcoma.

## 1. Introduction

Adult soft tissue sarcoma (STS) is a rare and biologically heterogeneous group of malignancies encompassing over 100 histological subtypes, as defined by the current WHO classification [1].

Most STS patients present with localised disease, typically treated with surgery and radiotherapy [2,3]. The benefit of perioperative chemotherapy varies by histology, grade, size, stage, and age. Prediction models such as Fédération Nationale des Centres de Lutte Contre le Cancer (FNCLCC), Sarculator, and Personalised Sarcoma Care (PERSARC) normograms estimate progression-free survival (PFS) and overall survival (OS), but are mainly validated for extremity and trunk STS [4,5]. Histological response is under investigation as a surrogate marker [6]. Despite optimal management, approximately 35% of patients develop metastatic disease within five years of diagnosis [7,8], with limited prognosis (5-year OS 15–30%; median 1.5–2 years). Treatment is based on anthracyclines, with options like trabectedin, pazopanib, and eribulin in later lines [2,9,10]. Robust biomarkers for risk stratification and therapeutic decision-making in both curative and advanced settings remain an urgent unmet need.

The advent of immune checkpoint inhibitors (ICIs) has transformed treatment paradigms in several solid malignancies, including malignant melanoma and non-small cell lung cancer (NSCLC) [11,12,13,14,15]. Established biomarkers such as programmed death-ligand 1 (PD-L1/PD-1) expression, microsatellite instability (MSI), defective mismatch repair (dMMR), and tumour mutational burden (TMB) help predict ICI efficacy [16]. However, in STS, neither PD-1/PD-L1 expression nor tumour-infiltrating lymphocyte (TIL) density has shown consistent prognostic or predictive value across most subtypes [17]. Exceptions include high TMB in angiosarcoma (AS) and the presence of B-cell-rich, tertiary lymphoid structure (TLS) in undifferentiated pleomorphic sarcoma (UPS) and dedifferentiated liposarcoma (DDLPS), both associated with better prognosis and enhanced ICI response [18,19,20,21]. Accordingly, ICIs such as pembrolizumab and nivolumab have been recommended for selected subtypes of advanced or metastatic STS—including UPS [2,22,23]. Combination immunotherapies targeting multiple immune checkpoints—such as PD-1/PD-L1, cytotoxic T-lymphocyte–associated protein 4 (CTLA-4), or lymphocyte-activation gene 3 (LAG-3)—are also under active investigation in UPS and AS [19,24]. Conversely, additional components of the tumour microenvironment (TME)—including CD68^+^ tumour-associated macrophages (TAMs), regulatory T cells (Tregs), and myeloid-derived suppressor cells (MDSCs)—have been implicated in immune evasion and resistance to ICI, contributing to poor outcomes [19,20]. In recent years, Regnase-1 (also known as MCPIP1 or RNase 1; encoded by the gene *ZC3H12A*) has emerged as an additional immunoregulator across multiple cell populations. This cytoplasmic endoribonuclease is strongly—but largely transiently—induced by pro-inflammatory cytokines and chemokines, such as TNF-α or IL-1β, thereby maintaining immune homeostasis, e.g., it downregulates IL6 and IL12B transcripts in monocytes and restricts c-Rel, OX40 and IL2 in T cells [25]. Mechanistically, Regnase-1 mediates selective, UPF1-dependent degradation of inflammation-associated mRNAs. In preclinical models, Regnase-1 knockdown enhanced MDSC recruitment and tumour immune evasion, reducing survival in murine pancreatic cancer [26] as well as in early stages of colorectal cancer [27]. In this retrospective single-centre study, we assessed Regnase-1 distribution and prognostic relevance in non-translocation-driven STS (leiomyosarcoma (LMS), UPS, AS), and explored its utility as a surrogate marker of an activated, pro-inflammatory TME. This analysis builds on prior data, including the reported inactivating effects of Regnase-1 on immunosuppressive TME components such as MDSCs. Additional immune markers—TILs, PD-1/PD-L1, T-cell immunoglobulin and mucin-domain containing protein 3 (TIM-3)/Galectin-9, LAG-3, T-cell immunoreceptor with Ig and ITIM domain (TIGIT), and CD68^+^ TAMs—were analysed exploratorily. Peritumoural CD8^+^ and CD4^+^ lymphocytes were frequent and correlated with PD-1 and TIGIT expression but lacked prognostic relevance, consistent with TIL exhaustion. High CD68^+^ TAM infiltration was associated with shorter mOS (13.0 months vs. NR; *p* = 0.0274) and higher mortality (HR = 2.0; 95% CI 1.1–3.7; *p* = 0.0325). By contrast, Regnase-1^+^ tumours—predominantly UPS—showed longer mOS (17.0 months vs. NR; *p* = 0.0247) and lower mortality (univariate: HR = 0.3; 95% CI 0.11–0.92; *p* = 0.0343; multivariate: HR = 0.4; 95% CI 0.15–0.96; *p* = 0.0413). STS with high Regnase-1 expression were enriched for immune and inflammatory regulators, including NF-κB and interferon-response programs, with reduced TGF-β signalling, consistent with a pro-inflammatory STS-TME and favourable prognosis. In contrast, among patients receiving adjuvant radiotherapy, high Regnase-1 expression was associated with impaired mOS (21.0 months vs. NR; *p* = 0.0838) and increased mortality (univariate: HR 2.8; 95% CI 1.0–7.69; *p* = 0.0491; multivariate: HR 2.8; 95% CI 0.90–8.85; *p* = 0.0547). No prognostic associations were observed in the palliative chemotherapy cohort.

## 2. Materials and Methods

### 2.1. Patient Collective

All patients diagnosed with AS, LMS, or UPS at the Institute of Pathology, University of Lübeck, between 2000 and 2020 were included; only tumour samples from initial diagnosis were analysed. We identified 101 cases (26 AS, 38 LMS, and 37 UPS). To exclude liposarcoma, mouse double minute 2 (MDM2) amplification was assessed by fluorescence in situ hybridisation (FISH) in three blocks, evaluating >100 cell nuclei and quantifying 30 nuclei. No MDM2 amplification was detected (available upon request). Epidemiological variables included sex, age at diagnosis, primary tumour location, histological grade, and metastatic status, as well as treatment data (chemotherapy, radiotherapy, surgical resection, and resection status), were collected. Follow-up was available until 1 September 2023 via ORBIS at University Hospital Schleswig-Holstein, with outpatient records. Ten patients were excluded owing to incomplete pathological or clinical data. The study was approved by the local ethics committee of the University of Lübeck (reference number 21-002), conducted in accordance with the Declaration of Helsinki, and data were anonymised prior to analysis.

### 2.2. Immunohistochemical Staining (IHC)

Preparation of formaldehyde-fixed paraffin-embedded (FFPE) tissue samples and the procedure of IHC were performed according to the manufacturers’ instructions and are described in the Supplements. A full list of all materials and software used is provided in Supplementary Tables S1 and S2. The used antibodies are listed in Table 1.

### 2.3. Microscopy

Stained slides were analysed by an experienced pathologist at the Institute of Pathology, University of Lübeck, using a BX50 microscope (Olympus, Tokyo, Japan) at 400× magnification (HPF). For CD4, CD8, CD68, PD-1, TIM-3, LAG-3, and TIGIT, cells were counted in two regions: one intratumoural HPF (centre, C) and one HPF in adjacent healthy tissue (periphery, P). PD-L1 expression was assessed using Immune Cell Score (IC), Tumour Proportion Score (TPS), and Combined Positive Score (CPS). Galectin-9 staining was evaluated separately in immune and tumour cells, while Regnase-1 expression was assessed exclusively in tumour cells (Table 2). A curated set of primary IHC images is provided in “Supplementary Materials: Original Pathology Images”.

Immunohistochemical scoring was performed independently by two experienced pathologists using standardised criteria and blinded to clinical outcomes. Positive and negative controls were included according to standard diagnostic protocols. Inter- and intra-observer variability were not formally quantified.

### 2.4. Analysis of the Association Between Regnase-1 Expression and Survival in a Curated “The Cancer Genome Atlas” (TCGA) Sarcoma Cohort

#### 2.4.1. Data Retrieval

Clinical BCR Biotab supplements for TCGA-Sarcoma (SARC) were downloaded using TCGAbiolinks (v2.36.0). Histologic diagnoses and key clinicopathological variables were extracted to curate a biologically homogeneous cohort by selecting UPS, LMS, and DDLPS via regex on histology and excluding translocation-driven sarcomas (synovial sarcoma, Ewing sarcoma, myxoid liposarcoma). RNA-seq data (TCGA.SARC.sampleMap/HiSeqV2) were obtained from the UCSC Xena TCGA hub using UCSCXenaTools (v1.6.1). Log2(expr+1) *Regnase-1* expression values were mapped to 12-character patient barcodes, and the median was used to define positive and negative groups (log2 median = 6.8979; Wilcoxon effect size r = 0.864). OS and PFS were retrieved from the Pan-Cancer Clinical Data Resource via pancanAtlas Xena and filtered for SARC.

#### 2.4.2. Gene Expression Analysis

Differential expression was analysed using limma (v3.64.3), retaining genes with adequate variability (IQR > 5th percentile) and <5% missing data. Principal component analysis was used to assess clustering by *Regnase-1* expression. *Regnase-1*-high and -low tumours were compared using limma::lmFit with empirical Bayes moderation (eBayes(trend = TRUE)). To limit biologically negligible effects in the large cohort (*n* = 212), treat was applied with a minimum log2 fold-change of 0.4 (~1.32-fold). Multiple testing was controlled using Benjamini–Hochberg FDR, with significance set at FDR < 0.05.

#### 2.4.3. Pathway Enrichment Analysis

Gene set enrichment was performed using GAGE (v2.58.0) on log2 fold-change values with MSigDB Hallmark gene sets (msigdf v2024.1) filtered to 10–500 genes. The unpaired gage method was used, and significance was defined as q-value < 0.05; stat.mean reflects the direction and magnitude of pathway differences between *Regnase-1*-high and low groups.

#### 2.4.4. Transcription Factor Activity Inference

Upstream transcriptional factor (TF) activity was inferred using decoupleR (v2.14) with CollecTRI regulons. Differential expression t-statistics served as input for the Univariate Linear Model (run ulm()) to estimate TF activity from target gene expression, including regulons with ≥10 target genes. TF scores indicate regulatory activity shifts, with positive values reflecting increased activity in *Regnase-1*-high tumours; significance was defined as adjusted *p* < 0.01 (Benjamini–Hochberg).

### 2.5. Statistics

#### 2.5.1. Survival Analysis in the Primary Cohort

CD68^+^ TAMs and Regnase-1 were prespecified as primary markers; all other immune markers were analysed in an exploratory fashion. Given the exploratory design and small subcohorts, no correction for multiple testing was applied; analyses were hypothesis-generating and focused on effect sizes, cross-subcohort consistency, and biological plausibility, with statistical significance interpreted descriptively. The cohort was analysed overall and stratified by histopathological subtype (AS, LMS, UPS). Metric biomarker data were non-normally distributed and are presented as mean, standard deviation, minimum, median, maximum, and interquartile range; categorical variables as absolute and relative frequencies. Biomarker expression was dichotomised at the median (“positive” above median; “negative” at or below median) for the entire cohort and subgroups; if the median was zero, values > 1 were defined as “positive.” Results are shown in Supplementary Tables S3–S7. Associations between biomarkers were assessed using Spearman Rho’s correlation coefficients (ρ), interpreted as weak (│0.1│–│0.3│), moderate (│0.3│–│0.5│), or strong (│>0.5│). Survival analyses were restricted to biomarkers with ≥30/70% or 70/30% positive/negative distributions; for markers with multiple localisations, the localisation with the highest case number or more balanced distribution was selected. Survival was analysed using Kaplan–Meier curves, with median survival reported if >50% of cases experienced an event within 60 months. Differences were assessed using the log-rank test, followed by univariate and multivariate Cox regression to estimate 60-month mortality risk. A *p*-value ≤ 0.05 was considered significant. To limit overfitting, multivariate models included only prespecified covariates (grading, CD68^+^ TAMs, Regnase-1) at approximately one variable per 8–10 events.

#### 2.5.2. Survival Analysis in the TCGA-SARC Cohort

Continuous Cox Models (Primary Analysis)

*Regnase-1* expression was standardised (mean = 0, SD = 1) and modelled continuously: Surv(time, event) ~ scale(*Regnase-1*_log2) for OS and PFI. HRs represent the effect per SD increase.

Model Diagnostics

Linearity was assessed by comparing linear and restricted cubic spline Cox models (3 df) using likelihood ratio tests. Proportional hazards assumptions were checked with scaled Schoenfeld residuals (cox.zph()). Model discrimination was evaluated by Harrell’s concordance index; overall fit by likelihood ratio, Wald, and score tests.

Dichotomized Analysis (Secondary Analysis)

For clinical clarity, patients were split into high/low *Regnase-1* by median. Kaplan–Meier curves and log-rank tests compared survival; HRs and 95% CIs were estimated using univariate Cox models.

Only patients with complete, non-negative survival data were included. Statistical significance was set at *p* < 0.05. Mann–Whitney U test was used for *Regnase-1* expression comparisons.

## 3. Results

### 3.1. Study Population

The clinicopathological characteristics of the cohort are summarised in Table 3. Overall, 65.9% of patients were female and 34.1% male, with a mean age of 66.4 (28–89) years. The cohort included 27.5% AS, 36.3% LMS, and UPS. Most sarcomas were located in the extremities or the deep and superficial trunk. Within the LMS subgroup, uterine LMS (uLMS) accounted for 27% of cases; to assess immune markers specifically in soft tissue LMS, uLMS cases were excluded to generate a refined subcohort. Most tumours were high grade (G3, 58%) or intermediate grade (G2, 28%), followed by G1 (12%). Metastatic disease was present in 51.3% of patients (M1), while 48.6% were M0. Complete (R0) resection was achieved in 71.2% of cases; 28% were R1, and 27% had an unspecified resection status (Rx). For further analyses, additional subcohorts were defined: surgery without radiotherapy, surgery with radiotherapy, and predominantly palliative chemotherapy. Six G1 sarcomas were treated with surgery alone; treatment data were unavailable for the remaining four G1 cases.

### 3.2. Characterisation of the Immunologic TME of STS

#### 3.2.1. Distribution of Immune Markers

The expression of CD4, CD8, CD68, PD-L1, PD-1, TIM-3, TIGIT, LAG3, and Regnase-1 was assessed in 91 samples across three sarcoma subtypes; results are shown in Supplementary Tables S3–S7 as well as in Table 4. More than 50% of cases were positive for CD4, CD8, and CD68, with comparable expression across subtypes and between intra- and extratumoural regions. CD68^+^ TAMs include both pro-inflammatory M1-like (CD68^+^/CD80^+^) and immunosuppressive M2-like (CD68^+^/CD163^+^/CD206^+^) macrophages, which suppress CD8 T-cell activity via IL-10 and TGF-β. Given the reported predominance of M2-like TAMs in high-grade sarcomas and our TCGA-SARC data showing a significant association between TAM infiltration and M2 polarisation in both *Regnase-1*-low and -high tumours (Supplementary Figure S3A), immunohistochemical discrimination of TAM subsets was not performed. PD-L1 expression assessed by CPS was generally low, with the highest prevalence in UPS (27%). In contrast, PD-1 and TIGIT were frequently expressed across subtypes and regions, with positivity rates of 36–47% and 52–65%, respectively. Regnase-1 expression was restricted to intratumoural regions in 56.6% of cases, with similar levels across AS, LMS, and UPS. Other immune checkpoints, including TIM-3, Galectin-9, and LAG-3, were largely absent or showed only low-level positivity (data available upon request).

#### 3.2.2. Correlation of Immune Markers Within TME

We explored biomarker expression profiles to identify combinatorial patterns. An overview of all correlations assessed using Spearman’s Rho correlation coefficients (ρ), stratified by tumour subtype, is provided in Supplementary Tables S8–S10. In AS and UPS, intratumoural CD4^+^ and CD8^+^ lymphocytes were significantly intercorrelated (AS: ρ = 0.55; 95% CI = 0.15–0.79; *p* = 0.0082; UPS: ρ = 0.65; 95% CI = 0.38–0.81; *p* = 0.0001). Intratumoural CD8^+^ lymphocytes correlated positively with intratumoural CD68^+^ TAMs in AS (ρ = 0.74; 95% CI = 0.42–0.90; *p* = 0.0002) and moderately in LMS (ρ = 0.41; 95% CI = 0.07–0.67; *p* = 0.0184), but not in UPS. In AS and UPS, CD4 positivity was moderately to strongly associated with intratumoural PD-1 expression (AS: ρ = 0.45; 95% CI = 0.04–0.73; *p* = 0.0305; UPS: ρ = 0.58; 95% CI = 0.29–0.78; *p* = 0.0003), and CD8 positivity also correlated significantly with PD-1 (AS: ρ = 0.61; 95% CI = 0.23–0.82; *p* = 0.0029; UPS: ρ = 0.62; 95% CI = 0.34–0.80; *p* = 0.0001). No significant associations were observed in LMS. Despite generally low PD-L1/CPS expression, intratumoural CD8^+^ lymphocytes correlated strongly with PD-L1/CPS in AS, LMS, and UPS (AS: ρ = 0.58; *p* = 0.0047; LMS: ρ = 0.41; *p* = 0.0171; UPS: ρ = 0.58; *p* = 0.0004). Intratumoural CD68^+^ TAMs also correlated with PD-L1/CPS in AS (ρ = 0.48; *p* = 0.0320) and LMS (ρ = 0.48; *p* = 0.0055). Across all subtypes, intratumoural TIGIT expression correlated strongly with intratumoural PD-1 in AS (ρ = 0.57; *p* = 0.0075), LMS (ρ = 0.41; *p* = 0.0175), and UPS (ρ = 0.55; *p* = 0.0008). In AS, TIGIT also correlated with intratumoural CD68^+^ TAMs (ρ = 0.57; *p* = 0.0086), while in UPS, intra- and extratumoural TIGIT correlated with CD8^+^ lymphocytes in the respective compartments (ρ = 0.59; *p* = 0.0002). Correlations between Regnase-1 and other immune markers were also analysed. In LMS, Regnase-1 expression showed inverse correlations with PD-1 (ρ = −0.45; 95% CI = −0.70–0.10; *p* = 0.0120) and CD68^+^ TAMs (ρ = −0.45; 95% CI = −0.70–0.11; *p* = 0.0107). Across predefined cohorts, no significant associations between Regnase-1 and CD68^+^ TAMs were observed (Supplementary Table S11).

### 3.3. Association Between Immune Markers and Clinical Outcome

#### 3.3.1. Survival Analysis in the Total Cohort

**Figure 1:**
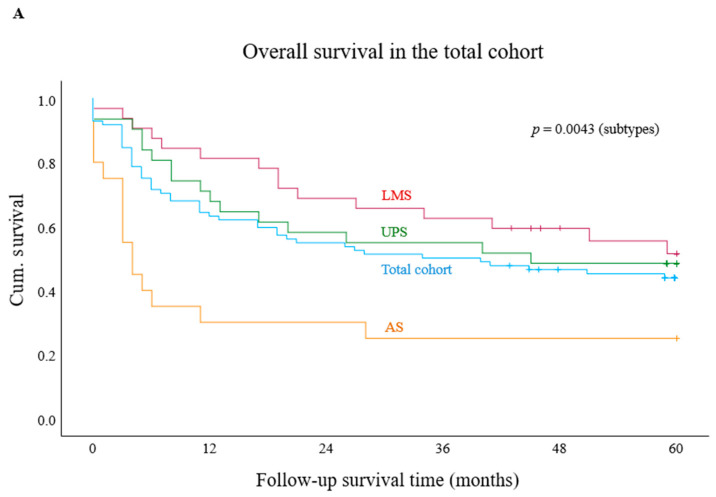

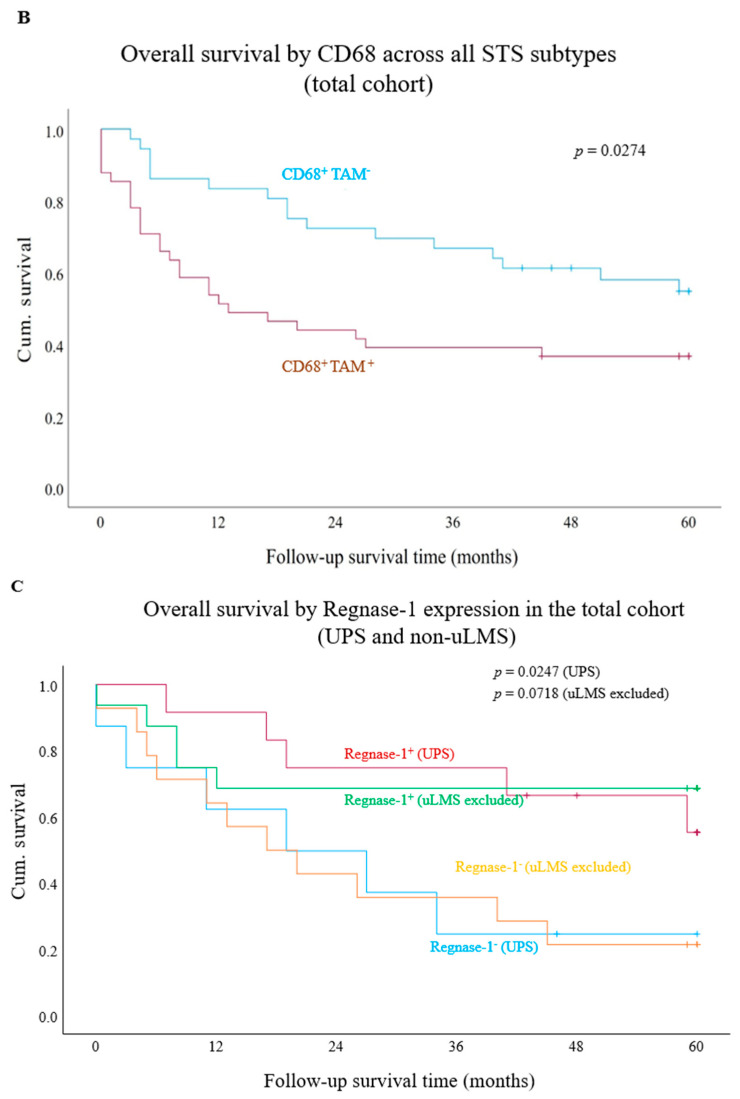
Kaplan–Meier survival analysis in the total cohort and according to tumour subtype (**A**), and stratified by immune marker expression (**B**,**C**). (**A**) Median overall survival in the total cohort and by histological tumour subtype. (**B**) Patients with CD68-negative tumours demonstrated significantly improved median overall survival compared to those with CD68-positive tumours in the total cohort (*p* = 0.0274). (**C**) Survival analysis showed a significant advantage for Regnase-1-positive undifferentiated pleomorphic sarcoma compared with Regnase-1-negative (*p* = 0.0247). After exclusion of uterine leiomyosarcoma, a strong numerical trend toward better median overall survival was observed between Regnase-1-positive and Regnase-1-negative tumours (*p* = 0.0718). Abbreviations: undifferentiated pleomorphic sarcoma, UPS; leiomyosarcoma, uLMS. + represent “censored cases”.

We first assessed OS rates in the entire cohort and then stratified the analysis by AS, UPS, and LMS. The 2-year OS for the overall cohort was 54%. The lowest rate was observed in AS (35%), followed by UPS (58%) and LMS (69%) (Figure 1A). Kaplan–Meier analyses assessed associations between biomarker expression and OS in the total cohort and tumour subtypes. Significant findings were followed by univariate and, where applicable, multivariate Cox regression to estimate HRs. In the total cohort, univariate Cox regression identified G3 (HR = 1.8, *p* = 0.0371), M1 (HR = 1.9, *p* = 0.0264), and non-R0 resection (HR = 2.5, *p* = 0.0013) as adverse prognostic factors, while age was not significant. Peritumoural CD4 and CD8 expression showed no survival association (Supplementary Figures S1 and S2). Intratumoural PD-1 status was not associated with OS overall, although PD-1^+^ AS showed a trend towards poorer survival (*p* = 0.0745; Supplementary Figure S4A–D). PD-L1/CPS positivity also tended to shorter mOS compared with PD-L1^−^/CPS^−^ cases (*p* = 0.1390; Supplementary Figure S5A). Intratumoural Galectin-9 showed no survival associations (Supplementary Figure S6A–C), whereas intratumoural TIGIT showed a trend towards improved survival in LMS (*p* = 0.0553; Supplementary Figure S7A–D). In contrast, intratumoural CD68^+^ TAMs were significantly associated with worse OS in the total cohort (mOS: 13.0 months vs. NR in CD68^−^ cases; *p* = 0.0274; HR = 2.0, 95% CI = 1.1–3.7, *p* = 0.0325), with no significant effects within subgroups. Kaplan–Meier curves are shown in Figure 1B and Supplementary Figure S3B–D. Elevated Regnase-1 expression was associated with improved survival (mOS: 17.0 vs. NR; *p* = 0.0247) and emerged as an independent predictor of reduced mortality risk in UPS (HR = 0.32; 95% CI = 0.11–0.92; *p* = 0.0343; Figure 1C). A trend towards prolonged mOS was also observed in the total cohort (*p* = 0.1440; Supplementary Figure S8A–C). As uterine LMS (uLMS) comprised 27% of the LMS subcohort, analyses were repeated after excluding these cases. Regnase-1^+^ tumours continued to show a trend towards improved mOS (*p* = 0.0720; Figure 1C), whereas no survival associations were observed for PD-1 or CD68^+^ TAMs (Supplementary Figure S9A,B). We further assessed tumour-associated TLS by histological estimation, given its reported prognostic and predictive relevance in UPS. However, additional IHC-based characterisation was not performed due to the limited number of evaluable cases (up to 9 of evaluable 22 UPS cases).

#### 3.3.2. Biomarker Impact on Survival in Surgically Treated Patients

**Figure 2:**
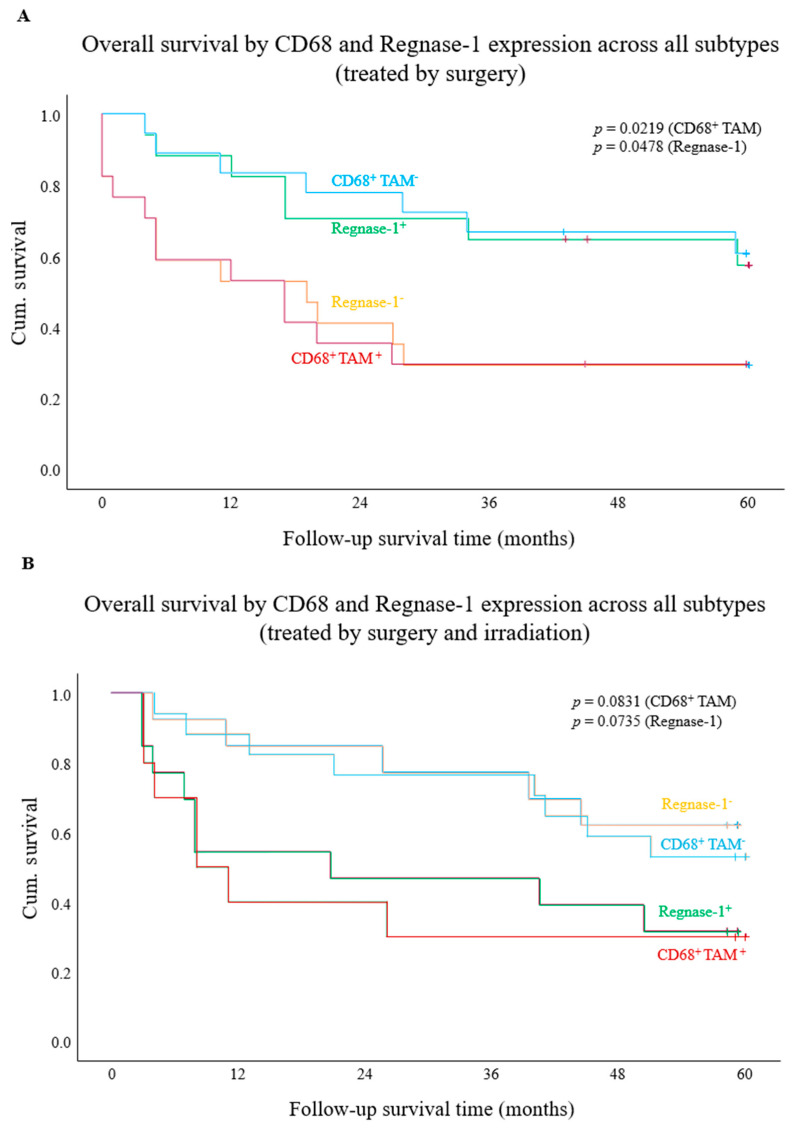
Kaplan–Meier survival analysis stratified by CD68 and Regnase-1 expression in patients treated with surgery, without (**A**) or with (**B**) irradiation. (**A**) CD68-positive tumours were associated with significantly worse overall survival than CD68-negative tumours (*p* = 0.0219). Regnase-1-positive tumours were associated with improved overall survival relative to Regnase-1-negative tumours (*p* = 0.0478). (**B**) CD68-negative tumours showed a trend toward longer overall survival compared with CD68-positive tumours (*p* = 0.0831). Regnase-1-positive tumours showed a non-significant trend toward shorter overall survival relative to Regnase-1-negative tumours (*p* = 0.0838). + represent “censored cases”.

To further delineate the survival relevance of PD-1, PD-L1, CD68, and Regnase-1 observed in the overall cohort, analyses were extended to treatment-based subcohorts (surgery alone, surgery plus irradiation, and chemotherapy). Due to limited case numbers, all STS entities were analysed jointly without histological stratification. In patients treated by only surgery, Kaplan–Meier log-rank analyses showed no significant survival association for PD-L1/CPS or PD-1 positivity (Supplementary Figure S10A,B). In contrast, intratumoural CD68^+^ TAMs were associated with significantly reduced mOS (17.0 vs. NR; *p* = 0.0219; Figure 2A). This finding was corroborated by Cox regression, demonstrating an increased mortality risk (HR = 2.8; 95% CI = 1.1–7.3; *p* = 0.0304). Consistent with the total cohort, Regnase-1 expression showed a robust association with improved mOS compared to Regnase-1^–^ tumours in patients treated solely with surgery (mOS: 19.0 months vs. NR; *p* = 0.0478; Figure 2A). In Cox regression analysis, there was a strong trend toward reduced mortality risk (HR = 0.4, 95% CI = 0.16–1.0; *p* = 0.0593).

#### 3.3.3. Biomarker Impact on Survival in Patients Receiving Surgery and Irradiation

In contrast to the other subcohorts, no significant associations were observed for PD-L1/CPS or PD-1 expression in patients receiving perioperative radiation therapy (Supplementary Figures S11 and S12). CD68^+^ TAMs showed a trend toward reduced mOS (8.0 vs. NR; *p* = 0.0831; HR = 2.5, 95% CI = 0.96–6.72; *p* = 0.0613; Figure 2B). High Regnase-1 expression was likewise associated with shorter mOS (21.0 vs. NR; *p* = 0.0735) and a significantly increased hazard (HR = 2.8, 95% CI = 1.0–7.69; *p* = 0.0491; Figure 2B).

#### 3.3.4. Biomarker Impact on Survival in Chemotherapy-Treated Patients

In the subgroup of patients treated with perioperative chemotherapy, no significant prognostic differences were observed for expression of PD-1, PD-L1, CD68, or Regnase-1 (Supplementary Figures S13–S16).

#### 3.3.5. Continuous Cox Regression for CD68^+^ TAMs and Regnase-1

Continuous Cox regression analyses of intratumoural CD68 and Regnase-1 expression were performed in treatment-defined subcohorts (Figure 1B and Figure 2A,B) to explore potential linear associations alongside dichotomised analyses. In the UPS subgroup, higher Regnase-1 expression trended toward improved survival (HR = 0.986, 95% CI 0.971–1.001, *p* = 0.060; *n* = 31, events = 20), though this did not reach statistical significance. In the surgery-only cohort, neither CD68 (HR = 1.003, 95% CI 0.998–1.007, *p* = 0.243) nor Regnase-1 (HR = 0.990, 95% CI 0.976–1.005, *p* = 0.182) correlated with overall survival. Likewise, no significant associations were observed in the radiotherapy cohort for CD68 (HR = 1.004, 95% CI 0.999–1.008, *p* = 0.149) or Regnase-1 (HR = 1.001, 95% CI 0.987–1.016, *p* = 0.865).

#### 3.3.6. Post Hoc Power Assessment for Cox Models (Schoenfeld Approximation)

To assess the robustness of significant survival associations, we performed a post hoc power sensitivity analysis using the Schoenfeld approximation for Cox/log-rank comparisons. For each significant dichotomised association in the primary and TCGA-SARC cohorts, the number of events required to detect the observed HR with 80% power (α = 0.05; *p* = 0.5) was estimated; for HR < 1, reciprocal values were used. Approximately 30 events were required for the Regnase-1 effect in irradiated patients (HR 2.8), 36 for the UPS-specific multivariable Regnase-1 effect (HR 0.39), and 66–106 for moderate associations. In TCGA-SARC, 82 OS events were observed versus ~106 required for the observed HR (0.58), indicating moderate but limited power. All results are summarised in Supplementary Table S12 and highlight that some nominally significant findings—particularly in small treatment-defined subgroups—may be imprecise and prone to overestimation.

#### 3.3.7. Integrated Survival Analysis: Grading, CD68^+^ TAMs and Regnase-1 as Independet Prognostic Factors

**Figure 3:**
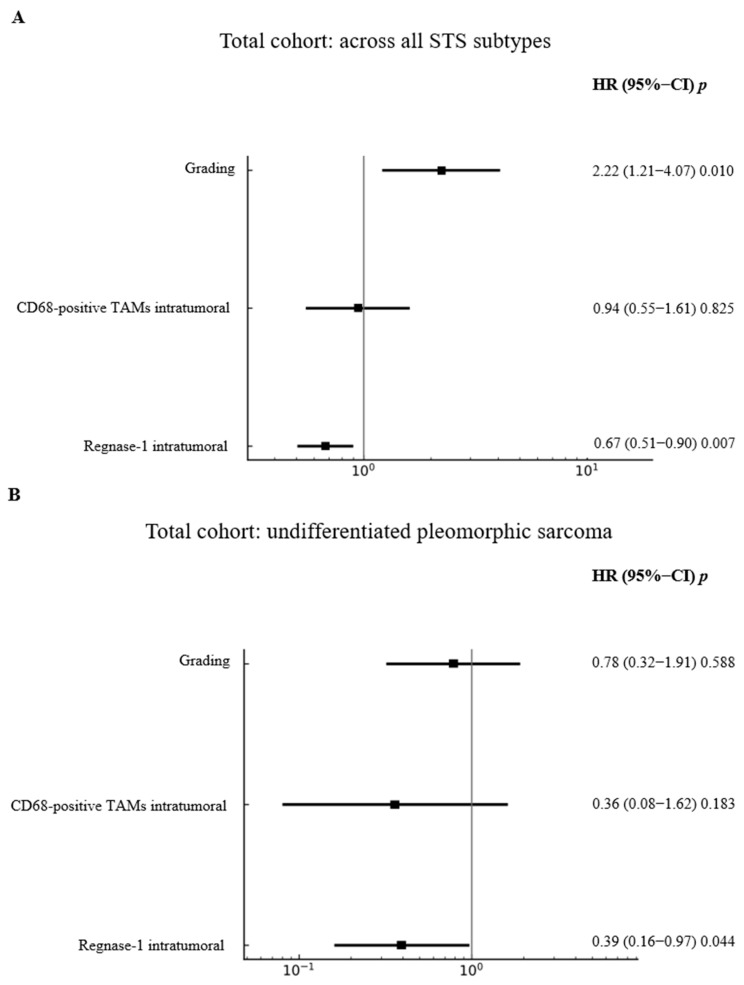

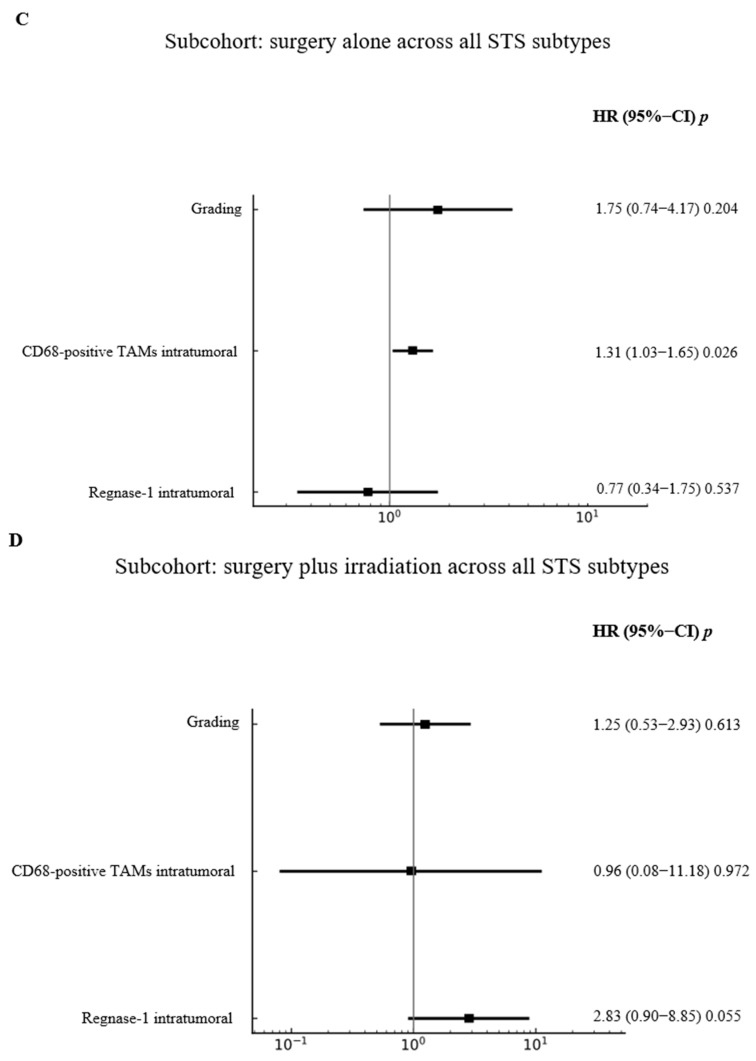
Multivariate Cox regression analysis stratified by grading, intratumoural CD68 expression, and Regnase-1 status in predefined cohort. (**A**) Higher grading was independently associated with a significantly increased risk of death (*p* = 0.010), whereas Regnase-1 positivity correlated with a significantly reduced risk of death (*p* = 0.007) in the total cohort across all sarcoma subtypes. (**B**) In the subgroup of undifferentiated pleomorphic sarcoma within the total cohort, Regnase-1 positivity remained significantly associated with a reduced risk of death (*p* = 0.044). (**C**) Among patients treated with surgery alone, Regnase-1 expression had no significant impact on the hazard ratio, whereas intratumoural CD68-positive TAMs were associated with shorter overall survival (*p* = 0.026). (**D**) In contrast, among patients receiving surgery plus irradiation, elevated Regnase-1 expression was conversely associated with an increased risk of death (*p* = 0.0547).

To account for the prognostic impact of Regnase-1 observed in prior univariate analyses, multivariate Cox regression analyses were performed in the total cohort and in the surgery-only and surgery-plus-irradiation subcohorts, including grading and intratumoural CD68^+^ TAMs as covariates. In the total cohort (Figure 3A), higher grading was associated with poorer prognosis (HR = 2.22; 95% CI = 1.21–4.07; *p* = 0.0102), while Regnase-1 positivity significantly reduced mortality risk (HR = 0.67; 95% CI = 0.51–0.90; *p* = 0.0074), in line with univariate results. In the UPS cohort (Figure 3B), high Regnase-1 expression independently reduced mortality by up to 39% (HR = 0.39; 95% CI = 0.16–0.97; *p* = 0.0443), whereas grading and CD68^+^ TAMs were not significant. In patients treated with surgery alone (Figure 3C), a high proportion of intratumoural CD68^+^ TAMs was associated with increased mortality (HR = 1.31; 95% CI = 1.03–1.65; *p* = 0.0266), while grading and Regnase-1 showed no prognostic impact. Conversely, in the surgery-plus-irradiation subgroup (Figure 3D), high Regnase-1 expression was associated with increased mortality (HR = 2.83; 95% CI = 0.90–8.85; *p* = 0.0547), consistent with univariate analyses, with no significant effects observed for the other markers.

### 3.4. Prognostic Relevance of Regnase-1 (Regnase-1) Expression in High-Malignant, Non-Translocation Driven STS: Analysis of an Independent TCGA Dataset

#### 3.4.1. Cohort Characteristics: TCGA-SARC Subset

To further examine clinical outcomes and transcriptomic associations, an independent TCGA-SARC cohort was established (Table 5A,B). Of 261 patients receiving standard therapies, 214 cases (UPS *n* = 50, LMS *n* = 104, DDLPS *n* = 58) were included in downstream analyses. Normalised expression data were available for 266 tumour samples (20,530 genes); after filtering, 212 cases with matched clinical and expression data were retained for integrated analysis. Survival data were available for 214 patients, with 82 OS and 116 PFS events among the 212 evaluable cases. *Regnase-1* expression showed a median log2 value of 6.8979 and an equal split between high (*n* = 106) and low (*n* = 106) expression groups (Table 5A,B), enabling integration across all three datasets (*n* = 212).

#### 3.4.2. Association Between Regnase-1 Expression and Survival Using Median Stratification

**Figure 4:**
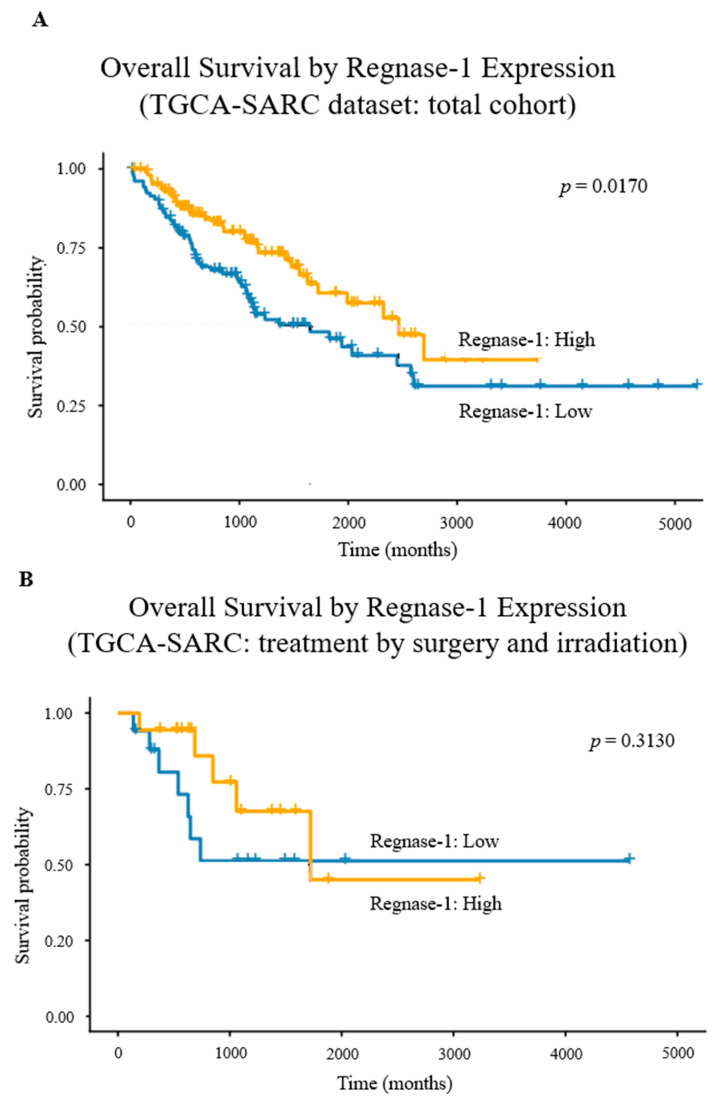
Kaplan–Meier survival analysis stratified by *Regnase-1* in aggressive, non-translocation-driven STS. (**A**) *Regnase-1*-positive tumours were associated with significantly longer overall survival than *Regnase-1*-negative tumours (*p* = 0.0170). (**B**) In patients receiving adjuvant irradiation, this effect was lost, yet a trend toward worse survival in the *Regnase-1*-positive group persisted (*p* = 0.3130). + represent “censored cases”.

*Regnase-1* expression was dichotomised at the median (Wilcoxon effect size r = 0.864; large). Expression distributions in the full cohort and subgroups are shown in Supplementary Figure S17A,B. *p*-values were calculated using the Mann–Whitney U test. Kaplan–Meier analysis demonstrated significantly improved OS in the *Regnase-1*^+^ group compared with *Regnase-1*^−^ patients (log-rank χ^2^ = 5.7; df = 1; *p* = 0.0248). In univariable Cox regression, *Regnase-1*^+^ status was associated with a reduced hazard of death (HR = 0.58; 95% CI = 0.37–0.91; *p* = 0.0170; *n* = 212; events = 82; Figure 4A), with a concordance index of 0.582 (SE = 0.029). As observed previously, the favourable prognostic effect of *Regnase-1* in the total cohort and in surgery-only patients was lost—and shifted towards a negative trend—after adjuvant radiotherapy. Validation in the TCGA-SARC cohort confirmed attenuation of this effect, with loss of statistical significance and a weak negative trend (*p* = 0.3130; Figure 4B).

#### 3.4.3. Differential Gene Expression Analysis Between Regnase-1-High and Regnase-1-Low Sarcoma Patients Using a Median-Split Model

To investigate differential gene expression, patients were stratified by *Regnase-1* expression using a median split, ensuring balanced groups, reproducibility across cohorts, and reduced risk of data snooping and inflated Type I error. After filtering, 17,133 genes were retained for analysis. Principal Component Analysis (PCA) demonstrated sample separation according to *Regnase-1* expression (Supplementary Figure S18A). A total of 140 genes were differentially expressed between *Regnase-1*^+^ and *Regnase-1*^−^ groups, with 122 upregulated and 18 downregulated in the *Regnase-1*^+^ group. *Regnase-1* itself showed a log2 fold change of 1.80 with an FDR of 1.94 × 10^−23^ (not shown in the volcano plot; Supplementary Figure S19B).

**Figure 5:**
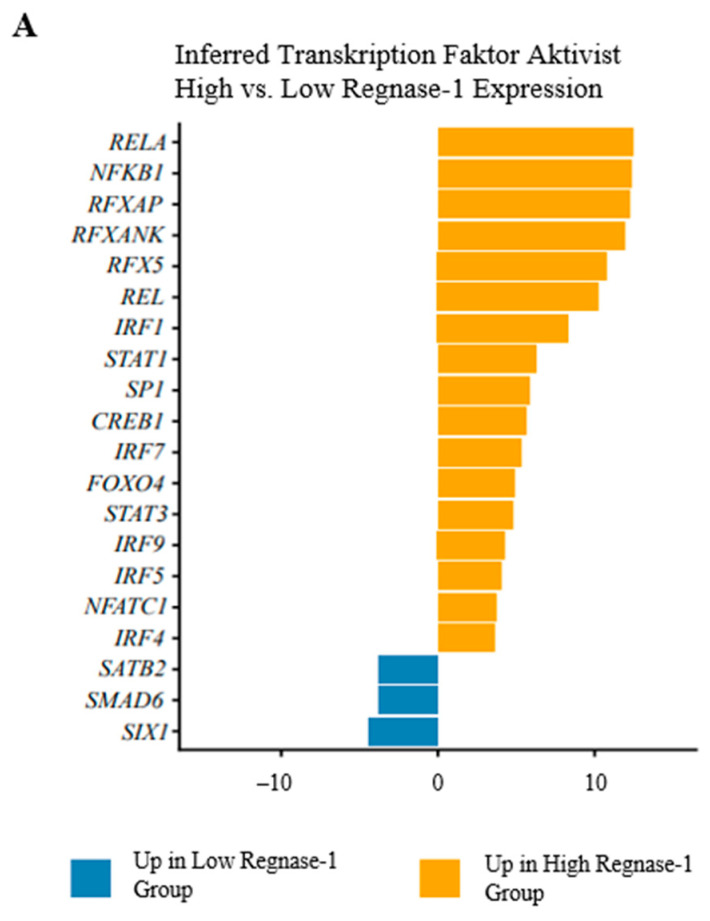

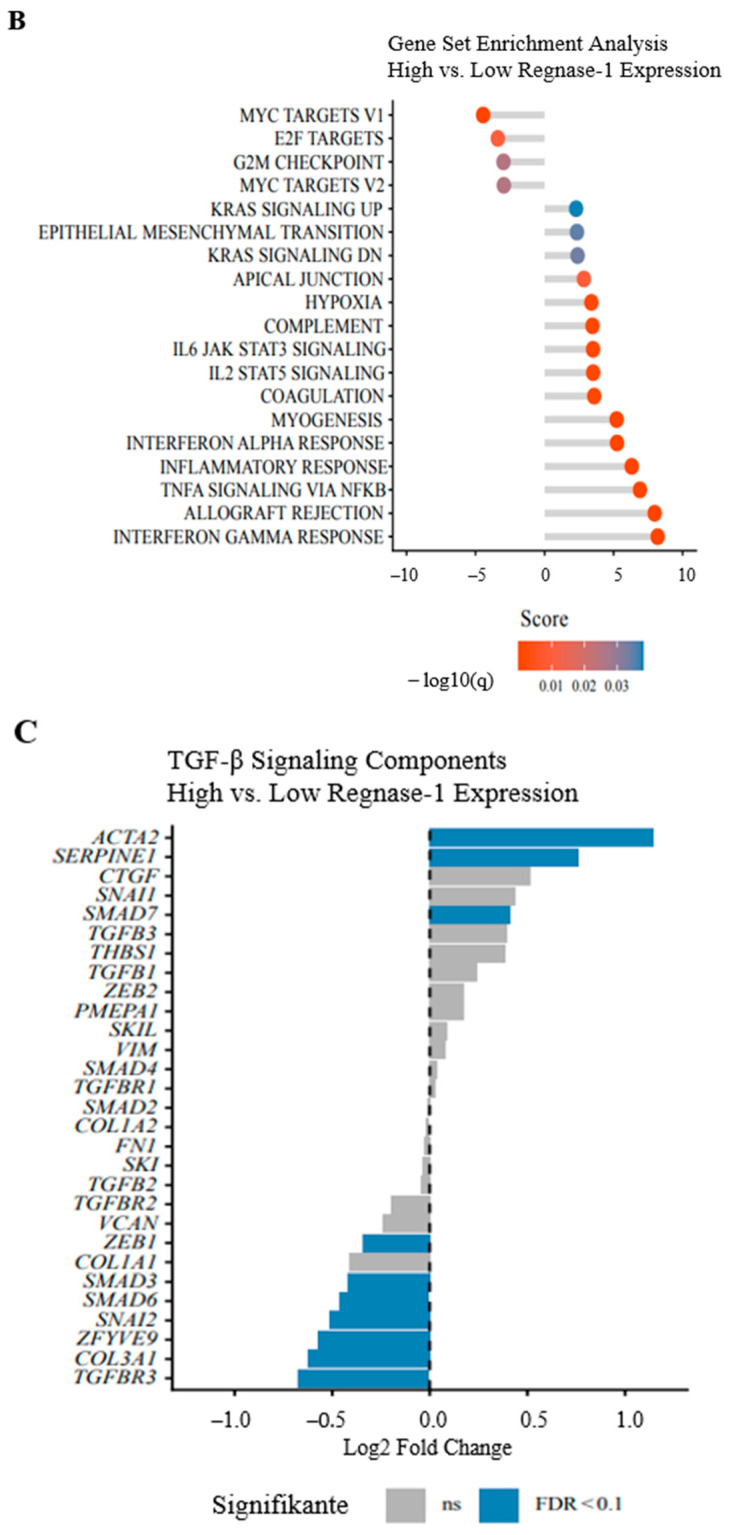
Transcriptional and pathway analysis of *Regnase-1*-high versus *Regnase-1*-low high-grade STS. (**A**) Transcription factor activity analysis. Inferred transcription factor activities using decoupleR with CollecTRI regulatory networks. Bars represent TF activity scores (positive = higher activity in *Regnase-1*-high tumours, negative = lower activity). Orange bars indicate transcription factors with increased activity in *Regnase-1*-high tumours, blue bars indicate decreased activity. Only transcription factors with FDR < 0.01 are shown. (**B**) Gene set enrichment analysis. GAGE analysis of Hallmark pathways using log2 fold-change values from differential expression analysis. Pathway activity scores represent mean log2 fold-change of genes within each pathway (positive = upregulated in *Regnase-1*-high tumours, negative = downregulated). Point colours indicate statistical significance (log10 q-value). (**C**) TGF-β signalling pathway components. Individual gene expression changes for TGF-β pathway components in *Regnase-1*-high versus *Regnase-1*-low tumours. Bars represent log2 fold-change (positive = upregulated in *Regnase-1*-high, negative = downregulated). Blue bars indicate genes with FDR < 0.1, gray bars indicate non-significant changes. All analyses compare *Regnase-1*-high (above median) versus *Regnase-1*-low (below median) expression groups. *N* = 212 patients from TCGA sarcoma cohort.

TF activity analysis is shown in Figure 5A and Supplementary Figure S19. Only limited transcriptional repression was observed, mainly involving reduced activity of *SIX1, SMAD6*, and *SATB2*, whereas *Regnase-1*-high tumours exhibited broad transcriptional activation. Immune and inflammatory regulators, including *RELA*, *NFKB1*, *REL*, *RFXAP*, *RFXANK*, and *RFX5*, were strongly induced, consistent with NF-κB activation. Concurrent upregulation of *IRF1*, *IRF4*, *IRF5*, *IRF7*, and *IRF9*, together with increased *STAT1* and *STAT3*, indicated enhanced interferon-driven inflammatory programs. Pathway analysis revealed enrichment of immune- and stress-related pathways, including TNF-α signalling via NF-κB, hypoxia, complement activation, coagulation, myogenesis, and apical junction pathways, reflecting microenvironmental activation and tissue remodelling in *Regnase-1*-high tumours (Figure 5B). MYC targets V1/V2, E2F targets, and the G2M checkpoint were downregulated, consistent with reduced proliferation, despite increased KRAS signalling without detectable adverse survival effects. Oncogenic TGF-β signalling was coordinately suppressed in *Regnase-1*-high tumours, including reduced expression of *SMAD3*, the *SMAD2/3* co-adapter *ZFYVE9*, and the co-receptor *TGFBR3*. In line with this, the endogenous inhibitor *SMAD7* was markedly upregulated, likely contributing to reduced TGF-β signalling. Notably, pro-fibrotic markers *ACTA2*, *SERPINE1*, and EMT pathways—typically induced by TGF-β—were strongly increased (Figure 5C).

## 4. Discussion

STS represents some of the rarest adult malignancies and encompasses a broad spectrum of histological subtypes [1]. In localised disease, ~60% of patients can be cured with surgery, perioperative radiotherapy and/or chemotherapy, providing additional benefit in selected cases [2,4]. For metastatic STS, particularly high-grade entities such as UPS and AS, prognosis remains poor (5-year OS 15–30%; median OS 1.5–2 years) [8], and the lack of robust biomarkers for risk stratification and therapeutic guidance continues to represent a critical unmet need. While ICIs have reshaped outcomes in several solid and haematological malignancies [11,12,13] and biomarkers such as PD-1/PD-L1, MSI/dMMR, and TMB support treatment selection across tumour types, their prevalence and clinical utility in STS remain limited and inconsistent [17].

In this study, we systematically assessed Regnase-1 as a surrogate marker of an activated TME in patients with non-translocation-driven, higher-grade STS, and exploratorily analysed TILs, PD-1/PD-L1, TIM-3/Galectin-9, LAG-3, TIGIT, and CD68^+^ TAMs. Across the cohort and histological subtypes, CD4^+^ and CD8^+^ lymphocytes were abundant, and PD-1 and TIGIT expression was frequent, each observed in >50% of cases. TIL subsets were strongly intercorrelated and closely associated with intra- and peritumoural PD-1 and TIGIT expression, yet none of these parameters associated with survival, supporting functional TIL exhaustion. This pattern aligns with previous work describing prominent lymphocyte infiltration in the STS-TME [24], generally limited prognostic relevance for CD8^+^, modest effects for CD4^+^, and potential benefit linked to CD20^+^ TLS [28,29]. Further, it is consistent with reports of PD-1 and TIGIT upregulation in exhausted CD4^+^ and CD8^+^ T cells, as well as in FOXP3^+^ Tregs and intratumoural NK cells, in aggressive STS—features typical of an immunologically “cold” TME [29,30,31,32]. By contrast, intratumoural CD68^+^ TAMs—while correlating with CD8^+^ TILs—were associated with reduced survival in the overall target cohort, most clearly among patients treated by surgery alone, underscoring a genuine adverse prognostic impact of TAMs in high-grade STS. This observation is biologically plausible given the established role of TAMs in immune evasion and ICI resistance [19,20,33]. In most high-grade STS, M2-like TAMs (CD68^+^/CD163^+^/CD206^+^) predominate over both TILs and pro-inflammatory M1-like CD68^+^/CD80^+^ macrophages, suppressing CD8 T-cell activity and promoting exhaustion via IL-10 and TGF-β [34]. Their dominance has been linked to inferior survival in UPS, DDLPS, and LMS [35,36]. Notably, Regnase-1 contrasted with the “cold” immune landscape and emerged as a favourable prognostic factor in the overall cohort, most prominently in UPS. This association persisted in surgery-only patients, supporting biological robustness. However, in UPS, Regnase-1 expression did not significantly associate with CD68^+^ TAMs.

As mentioned above, continuous Cox regression analyses were additionally performed to assess potential linear associations and complement dichotomised results. The lack of significant findings in continuous models suggests that the observed survival differences are not driven by a linear dose–response relationship, but rather reflect threshold-dependent or context-specific effects, particularly in UPS and under radiotherapy.

Regnase-1 is an endogenous immunomodulator integrating microenvironmental cues, regulating apoptosis, differentiation, and angiogenesis, and limiting monocyte and T-cell activation via negative feedback. Although its regulation is incompletely understood, Regnase-1 is strongly induced by TNF-α, IL-1β, and monocyte chemoattractant protein-1 via MAPK and NF-κB activation, and can also be upregulated by IL-17A through JAK/STAT3 signalling; induction is typically transient and curtailed by proteasomal degradation via the TLR–IKK–IRAK1 complex, miRNAs such as miR-9 (upregulated by PDGF-BB), and autoregulation through its ribonuclease activity [25]. Overall, Regnase-1 facilitates rapid cellular adaptation while maintaining immune homeostasis. Recent data indicate tumour type-specific roles for Regnase-1 in oncogenesis. In neuroblastoma cell lines, enforced Regnase-1 expression reduced viability/proliferation by downregulating CTL1 and miRNA-3613-3p 37. In MCF7 breast cancer cells, Regnase-1 overexpression suppresses tumour growth by stabilising the tumour suppressor RGS2 and promoting decay of anti-apoptotic mRNAs such as Bcl2L1 and Bcl2A1; low expression correlates with poor long-term survival [37]. Conversely, suppression of Regnase-1 accelerated tumour progression in pancreatic cancer by enhancing MDSC recruitment and weakening cytotoxic T-lymphocyte immunity [26], and was associated with shorter survival in colorectal cancer, potentially via CXCL1/2/3 dysregulation [27]. Pro-tumourigenic activity has also been reported, with S100A8/A9-induced upregulation promoting tumour growth in colon cancer cell lines [38], whereas in the B16-Ova melanoma model, Regnase-1-null CD8^+^ T cells adopted long-lived effector characteristics with increased intratumoural persistence, translating into stronger antitumour activity [39]. Consistent with Regnase-1 as a marker of inflammatory activation, TF and signalling pathway analyses in the independent TCGA-SARC cohort linked high *Regnase-1* expression to activated *NF-κB*, *TNF-α*, and interferon pathways, alongside reduced activity of pro-oncogenic and mostly anti-inflammatory *TGF-β* signalling [40,41,42,43], supporting the favourable prognosis observed in *Regnase-1*^+^ high-grade STS. In contrast, among high-risk STS patients receiving adjuvant irradiation, high *Regnase-1* was associated with worse prognosis in uni- and multivariate analyses. TCGA-SARC patterns support plausible mechanisms: *SERPINE1* upregulation in *Regnase-1^+^* tumours—potentially driven by *NF-κB* and hypoxia pathways—may promote radioresistance via enhanced EMT and TME remodelling, as described in NSCLC and breast cancer [44,45,46,47]. In parallel, *KRAS*-signalling activation in *Regnase-1*-high STS—known in gastrointestinal cancer and NSCLC to promote aggressive behaviour and reduced radiosensitivity [48,49,50]—may further account for the adverse prognostic effect in this cohort.

Taken together, our high-grade STS cohort displayed a predominantly “cold” TME with abundant PD-1- and TIGIT-expressing TILs lacking prognostic impact, likely reflecting exhaustion in a TAM-rich context. CD68^+^ TAMs were associated with poor survival, whereas Regnase-1 marked a prognostically favourable, pro-inflammatory STS-TME—particularly in UPS—supported by TCGA-SARC validation. However, this benefit was lost in the adjuvant irradiation setting, where pro-fibrotic SERPINE1 induction and EMT-/TME-remodelling programmes may impair radiosensitivity and negate survival advantages.

## 5. Limitations of This Study

Some limitations should be acknowledged. The retrospective, single-centre design may introduce selection bias, and subgroup analyses were limited by restricted formal power. Although the overall cohort size is acceptable for a rare disease, clinically relevant stratifications—particularly UPS, surgery-only, irradiation-treated, and chemotherapy-treated patients—substantially reduced effective sample sizes. Post hoc Schoenfeld-based sensitivity analysis suggests that some nominally significant findings, especially in small treatment-defined subgroups, may be imprecise and prone to effect size overestimation. Moreover, dichotomisation may have introduced bias, given the non-normal distribution and frequent zero values of the biomarkers. Continuous analyses did not confirm these findings, likely reflecting limited sample sizes in the respective subcohorts, and therefore warrant cautious interpretation and validation. We did not further distinguish between M1- and M2-like TAMs in the primary cohort, given the known predominance of M2-like TAMs in high-grade STS; this will be addressed in future studies with larger cohorts. Mechanistic interpretations—particularly regarding divergent effects in irradiated vs. non-irradiated STS—remain correlative and are based on transcriptomic and immunologic associations within TCGA-SARC, leaving it unclear whether Regnase-1 reflects a pro-inflammatory TME or actively contributes to pathway activation.

Taken together, these findings are hypothesis-generating and require validation in larger, preferably multicentre cohorts before clinical implementation.

## 6. Conclusions

Our data identify Regnase-1 as a promising biomarker in UPS, displaying context-dependent prognostic relevance that warrants further validation. To refine this observation, we will quantify TLS to corroborate its established prognostic value in UPS, while concurrently assessing the impact of Regnase-1 through integrated survival analyses to determine whether its prognostic effect is independent of established factors.

To move beyond association, we will interrogate the functional role of Regnase-1 using knockdown and overexpression approaches in UPS cell models, examining effects on proliferation, apoptosis, migration, and inflammatory signalling. In parallel, comparative analyses of the STS-TME before and after radiotherapy will help clarify mechanisms of immune activation and potential radioresistance, particularly in Regnase-1–high tumours.

## Figures and Tables

**Table 1:** List of primary antibodies used for immunohistochemistry, including clone designation, producer, batch/lot number, and working dilution. Antibodies: CD4 (cluster of differentiation 4), CD8 (cluster of differentiation 8), PD-L1 (programmed death-ligand 1), PD-1 (programmed cell death protein 1), TIM-3 (T cell immunoglobulin and mucin-domain containing-3), LAG-3 (lymphocyte-activation gene 3), Galectin-9 (β-galactoside-binding lectin 9), CD68 (cluster of differentiation 68), TIGIT (T cell immunoreceptor with Ig and ITIM domains), and Regnase-1 (zinc finger CCCH-type containing 12A, Regnase-1). RTU = ready to use. ^1^ Ventana by Roche, Basel, Switzerland. ^2^ Cell Signaling Technology, Cambridge, UK. ^3^ Cell Marque Corporation, Rocklin, CA, USA. ^4^ Bethyl Laboratories Inc., Montgomery, TX, USA. ^5^ Thermo Fisher Scientific, Waltham, MA, USA.

Antibody	Clone	Producer	Batch Number	Dilution
CD4	SP35	Ventana ^1^	LOTH18587	RTU
CD8	SP57	Ventana ^1^	LOTH15791	RTU
PD-L1	E1L3N	Cell Signaling ^2^	Lot:18	1:50
PD-1	NAT105	Cell Marque ^3^	LOTV0002805	RTU
TIM-3	BLR033F	Bethyl Laboratories ^4^	LOT#1	1:200
LAG3	D2G4O	Cell Signaling ^2^	LOT6	1:200
Galectin-9	D9R4A	Cell Signaling ^2^	Lot: 1	1:200
CD68 (a pan-marker for TAMs)	KP-1	Ventana ^1^	LOTH15799	RTU
TIGIT	BLR047F	Bethyl Laboratories ^4^	LOT#3	1:500
Regnase-1	*Regnase-1*	Thermofisher ^5^	WF3142137	1:500

**Table 2:** Overview of the examination methods for each biomarker. CD4 (cluster of differentiation 4), CD8 (cluster of differentiation 8), CD68 (cluster of differentiation 68), PD-1 (programmed cell death protein 1), TIM-3 (T cell immunoglobulin and mucin-domain containing-3), TIGIT (T cell immunoreceptor with Ig and ITIM domains), and LAG-3 (lymphocyte-activation gene 3) were assessed in tumour center and periphery, quantified as positive cells per high-power field (HPF). PD-L1 (programmed death-ligand 1) expression was evaluated using tumour proportion score (TPS), immune cell score (IC), and combined positive score (CPS), each calculated as the percentage of positive cells relative to all tumour cells, the percentage of positive immune cells relative to all tumour cells and the percentage of the subtraction of positive tumour cells to positive immune cells relative to all tumour cells, respectively. Galectin-9 (β-galactoside-binding lectin 9) was analysed in tumour cells (positive tumour cells per total tumour cells) and immune cells (positive immune cell area per total area). Regnase-1 (zinc finger CCCH-type containing 12A, *Regnase-1*) was assessed in tumour centre as a percentage of positive tumour cells.

Biomarker	Division	Definition
CD4, CD8, CD68, PD-1, TIM-3, TIGIT, LAG-3	Intratumoural	Positive cells/HPF
	Extratumoural	Positive cells/HPF
PD-L1	TPS	(Positive tumour cells/all tumour cells) × 100 (%)
	IC	(Positive immune cells/all tumour cells) × 100 (%)
	CPS	((Positive tumour cells + positive immune cells)/all tumour cells) × 100
Galectin-9	Tumour cells	(Positive tumour cells/all tumour cells) × 100 (%)
	Immune cells	(Positive immune cells area/all area) × 100 (%)
Regnase-1	Intratumoural	(Positive tumour cells/all tumour cells) × 100 (%)

**Table 3:** Clinicopathological features of the overall cohort. Baseline characteristics include sex distribution, age at diagnosis (mean and range), tumour localisation (extremities, head and neck, trunk superficial, trunk deep, unknown), and tumour subtype (angiosarcoma, leiomyosarcoma, undifferentiated pleomorphic sarcoma). Tumour grading was classified as G1 (well differentiated), G2 (moderately differentiated), and G3 (poorly differentiated). Metastatic disease was classified as M0 (not metastatic), M1 (metastatic), and unknown. Resection status refers to resection margin status: R0 (complete resection with negative margins), R1 (microscopically positive margins), and unknown. Data on surgery, irradiation and chemotherapy are presented as absolute numbers with corresponding percentages.

Baseline Characteristics	*n* = 91 (100%)
Sex	
Female	60 (65.9%)
Male	31 (34.1%)
Age at diagnosis (years)	
Mean	66.4
Range	28–89
Tumour localisation	
Extremities	25 (27.5%)
Head and Neck	7 (8.4%)
Trunk, superficial	17 (20.5%)
Trunk, deep	23 (41.0%)
Uterine	11 (27.5%)
Unknown	8
Tumour subtype	
Angiosarcoma	25 (27.5%)
Leiomyosarcoma	33 (36.3%)
Undifferentiated pleomorphic sarcoma	33 (36.3%)
Grading	
G1	10 (12.8%)
G2	22 (28.2%)
G3	46 (58.9%)
Unknown	11
Metastatic disease	
M0	36 (48.6%)
M1	38 (51.3%)
Unknown	17
Resection status	
R0	47 (71.0%)
R1	19 (28.0%)
Rx	25 (27.0%)
Surgery alone	41 (60.3%)
with perioperative chemotherapy	7 (7.3%)
(doxorubicine plus ifosfamide or	
doxorubicine plus dacarbacine)	
Surgery with irradiation	27 (39.7%)
adjuvant irradiation	25 (92.6%)
Irradiation state unknown	23
Chemotherapy	
Yes	32 (43.2%)
perioperative	7 (21.8%)
palliative	25 (78.1%)
No	42 (56.7%)
Unknown	17

**Table 4:** Biomarker distribution according to tumour subtype. Expression of CD4 (cluster of differentiation 4), CD8 (cluster of differentiation 8), CD68 (cluster of differentiation 68), PD-1 (programmed cell death protein 1), and Regnase-1 (zinc finger CCCH-type containing 12A, Regnase-1) was assessed in tumour centre and periphery. Tumour subtypes are shown as (angiosarcoma), LMS (leiomyosarcoma), and UPS (undifferentiated pleomorphic sarcoma). Numbers represent absolute cases with positive and negative expression, given as *n* and percentage. I = intratumoural; E = extratumoural.

Biomarker	Localisation	Tumour Subtype (Missing *n*)	Expression	*n* (%)
CD4	I	AS (2)	Positive	9 (39.1%)
			Negative	14 (60.9%)
		LMS (2)	Positive	15 (48.4%)
			Negative	16 (51.6%)
		UPS (0)	Positive	16 (48.5%)
			Negative	17 (51.5%)
	E	AS (5)	Positive	9 (45.0%)
			Negative	11 (55.0%)
		LMS (5)	Positive	8 (28.6%)
			Negative	20 (71.4%)
		UPS (3)	Positive	18 (60.0%)
			Negative	12 (40.0%)
CD8	I	AS (3)	Positive	10 (45.5%)
			Negative	12 (54.5%)
		LMS (0)	Positive	14 (42.4%)
			Negative	19 (57.6%)
		UPS (0)	Positive	16 (48.5%)
			Negative	17 (51.5%)
	E	AS (7)	Positive	8 (44.4%)
			Negative	10 (55.6%)
		LMS (3)	Positive	13 (43.3%)
			Negative	17 (56.7%)
		UPS (3)	Positive	14 (46.7%)
			Negative	16 (53.3%)
CD68	I	AS (5)	Positive	10 (50.0%)
			Negative	10 (50.0%)
		LMS (1)	Positive	16 (50.0%)
			Negative	16 (50.0%)
		UPS (0)	Positive	14 (42.4%)
			Negative	19 (57.6%)
	E	AS (5)	Positive	7 (35.0%)
			Negative	13 (65.0%)
		LMS (1)	Positive	13 (40.6%)
			Negative	19 (59.4%)
		UPS (0)	Positive	14 (42.4%)
			Negative	19 (57.6%)
PD-L1	IC	AS (2)	Positive	3 (13.0%)
			Negative	20 (87.0%)
		LMS (0)	Positive	3 (9.1%)
			Negative	30 (90.9%)
		UPS (0)	Positive	16 (48.5%)
			Negative	17 (51.5%)
	TPS	AS (2)	Positive	3 (13.0%)
			Negative	20 (87.0%)
		LMS (0)	Positive	2 (6.1%)
			Negative	31 (93.9%)
		UPS (0)	Positive	9 (27.3%)
			Negative	24 (72.7%)
	CPS	AS (2)	Positive	3 (13.0%)
			Negative	20 (87.0%)
		LMS (0)	Positive	3 (9.1%)
			Negative	30 (90.9%)
		UPS (0)	Positive	9 (27.3%)
			Negative	24 (72.7%)
PD-1	I	AS (2)	Positive	9 (39.1%)
			Negative	14 (60.9%)
		LMS (0)	Positive	15 (45.5%)
			Negative	18 (54.5%)
		UPS (0)	Positive	13 (39.4%)
			Negative	20 (60.6%)
	E	AS (6)	Positive	8 (42.1%)
			Negative	11 (57.9%)
		LMS (3)	Positive	11 (36.7%)
			Negative	19 (63.3%)
		UPS (3)	Positive	14 (46.7%)
			Negative	16 (53.3%)
TIGIT	I	AS (4)	Positive	6 (28.6%)
			Negative	15 (71.4%)
		LMS (0)	Positive	16 (48.5%)
			Negative	17 (51.5%)
		UPS (0)	Positive	13 (39.4%)
			Negative	20 (60.6%)
	E	AS (4)	Positive	7 (33.3%)
			Negative	14 (66.7%)
		LMS (0)	Positive	10 (30.3%)
			Negative	23 (69.7%)
		UPS (0)	Positive	16 (48.5%)
			Negative	17 (51.5%)
Regnase-1	I	AS (5)	Positive	10 (50.0%)
			Negative	10 (50.0%)
		LMS (2)	Positive	14 (45.2%)
			Negative	17 (54.8%)
		UPS (1)	Positive	16 (50.0%)
			Negative	16 (50.0%)

**Table 5:** Baseline characteristics (A) and *Regnase-1* expression distribution in the TCGA-SARC cohort (B).

(**A**)			
**Tumour Subtype**		***n***	
Leiomyosarcoma		104	
Dedifferentiated liposarcoma		58	
Pleomorphic malignant fibrous histiocytoma/undifferentiated pleomorphic sarcoma		29	
Undifferentiated pleomorphic sarcoma		21	
(**B**)			
**Tumour Subtype**	**Regnase-1 Group**	***n***	**Prop**
Dedifferentiated liposarcoma	Negative	41	0.387
Dedifferentiated liposarcoma	Positive	17	0.160
Leiomyosarcoma	Negative	43	0.406
Leiomyosarcoma	Positive	61	0.575
Pleomorphic malignant fibrous histiocytoma/undifferentiated pleomorphic sarcoma	Negative	15	0.142
Pleomorphic malignant fibrous histiocytoma/undifferentiated pleomorphic sarcoma	Positive	14	0.132
Undifferentiated pleomorphic sarcoma	Negative	7	0.066
Undifferentiated pleomorphic sarcoma	Positive	14	0.132

## Data Availability

The data presented in this study are available on request from the corresponding author.

## Supplementary Materials

The following supporting information can be downloaded at: https://www.mdpi.com/article/10.3390/cancers18091419/s1.

## Abbreviations

The following abbreviations are used in this manuscript:

ACTA2, actin alpha 2, smooth muscle; AS, angiosarcoma; BCL2A1, BCL2-related protein A1; BCL2L1, BCL2-like 1; CPS, combined positive score; CTL1, choline transporter-like 1; CTLA-4, cytotoxic T-lymphocyte–associated protein 4; CXCL1/2/3, C-X-C motif chemokine ligand 1/2/3; DDLPS, dedifferentiated liposarcoma; dMMR, defective mismatch repair; E2F, E2F transcription factors; EMT, epithelial–mesenchymal transition; FISH, fluorescence in situ hybridisation; FNCLCC, Fédération Nationale des Centres de Lutte Contre le Cancer; FOXP3, forkhead box P3; G2M, G2/M phase; Galectin-9, galactoside-binding lectin 9; IC, immune cell score; ICI, immune checkpoint inhibitor; IHC, immunohistochemistry (immunohistochemical staining); IKK, IκB kinase; IL, interleukin; IRAK1, interleukin-1 receptor-associated kinase 1; IRF1, interferon regulatory factor 1; IRF4, interferon regulatory factor 4; IRF5, interferon regulatory factor 5; IRF7, interferon regulatory factor 7; IRF9, interferon regulatory factor 9; KRAS, KRAS proto-oncogene, GTPase; LAG-3, lymphocyte-activation gene 3; MAPK, mitogen-activated protein kinase; MCPIP1, monocyte chemotactic protein-1–induced protein 1; MDM2, mouse double minute 2; MDSCs, myeloid-derived suppressor cells; miRNAs, microRNAs; MSI, microsatellite instability; MYC, MYC proto-oncogene; NF-κB, nuclear factor kappa-light-chain-enhancer of activated B cells; NFKB1, nuclear factor kappa B subunit 1; NSCLC, non-small cell lung cancer; OS, overall survival; OX40, tumour necrosis factor receptor superfamily member 4; PCA, principal component analysis; PD-1, programmed cell death protein 1; PD-L1, programmed death-ligand 1; PDGF-BB, platelet-derived growth factor BB; PERSARC, Personalised Sarcoma Care; PFS, progression-free survival; REL, REL proto-oncogene, NF-κB subunit; RELA, RELA proto-oncogene, NF-κB subunit; RFXANK, regulatory factor X-associated ankyrin-containing protein; RFXAP, regulatory factor X-associated protein; RFX5, regulatory factor X5; RGS2, regulator of G-protein signalling 2; S100A8/A9, S100 calcium-binding protein A8/A9; SATB2, special AT-rich sequence-binding protein 2; SERPINE1, serpin family E member 1; SIX1, sine oculis homeobox homolog 1; SMAD3, SMAD family member 3; SMAD6, SMAD family member 6; SMAD7, SMAD family member 7; STS, soft tissue sarcoma; STAT1, signal transducer and activator of transcription 1; STAT3, signal transducer and activator of transcription 3; TAMs, tumour-associated macrophages; TCGA, The Cancer Genome Atlas; TGF-β, transforming growth factor beta; TGFBR3, transforming growth factor beta receptor 3; TIGIT, T-cell immunoreceptor with Ig and ITIM domains; TILs, tumour-infiltrating lymphocytes; TIM-3, T-cell immunoglobulin and mucin-domain containing protein 3; TLR, toll-like receptor; TLS, tertiary lymphoid structures; TMB, tumour mutational burden; TME, tumour microenvironment; TNF-α, tumour necrosis factor alpha; TPS, tumour proportion score; Tregs, regulatory T cells; UPF1, up-frameshift protein 1; UPS, undifferentiated pleomorphic sarcoma; ZFYVE9, zinc finger FYVE-type containing 9.
