# Supplementary material for: Prognostic Value of Regnase-1 in High-Grade Soft Tissue Sarcoma: Favourable in UPS, Yet Inverted in Adjuvantly Irradiated Patients
Source: Cancers (Basel). 2026 Apr 29;18(9):1419. doi: 10.3390/cancers18091419 (PMC13162679; doi:10.3390/cancers18091419)
Supplement: Supplementary file 1 [file cancers-18-01419-s001.zip › cancers-4242346-supplementary.pdf]

Preparation of Formaldehyde-fixed Paraffin-embedded (FFPE) Tissue Samples

To identify suitable pathological sections, all available H&E sections from the patients were collected and reviewed by an experienced pathologist at the Institute of Pathology, University of Lübeck. This process enabled confirmation of the diagnosis and qualitative assessment of the sections. Sections were selected to ensure that both tumour tissue and the adjacent healthy tissue were present, allowing evaluation of the tumour microenvironment. For each case, exactly one kerosene block was retrieved from the central archive at the University of Lübeck.

The FFPE tissue samples were sectioned in preparation for immunohistochemical staining using a Microm HM355S (Thermo Fisher Scientific, Waltham, MA, USA) at a thickness of 3.5 µm. Eleven sections per sample were mounted on slides (Roche, Basel, CHE) in a 50 °C water bath. Sections were then incubated at 70 °C for 60 minutes to dissolve the kerosene. Removal of residual kerosene and subsequent staining were performed using the fully automated Ventana BenchMark Ultra system (Roche, Basel, CHE).

Immunohistochemical Staining (IHC)

All samples were stained by indirect immunohistochemistry using peroxidase. The following antibodies were employed: CD4 (#18587, clone SP35, Roche, Basel, CHE), CD8 (#15791, clone SP57, Roche, Basel, CHE), PD-L1 (#18, clone E1L3N, Cell Signaling Technology, Cambridge, UK), PD-1 (#0002805, clone NAT105, Cell Marque Corporation, Rocklin, CA, USA), TIM-3 (#1, clone BLR033F, Bethyl Laboratories, Montgomery, TX, USA), LAG3 (#6, clone D2G4O, Cell Signaling Technology, Cambridge, UK), Galectin-9 (#1, clone D9R4A, Cell Signaling Technology, Cambridge, UK), CD68 (#15799, clone KP-1, Roche, Basel, CHE), TIGIT (#3, clone BLR047F, Bethyl Laboratories, Montgomery, TX, USA), and Regnase-1 (#3142137, clone *ZC3H12A*, Thermo Fisher Scientific, Waltham, MA, USA). Optimal staining conditions were determined by testing different antibody dilutions and staining protocols. The following dilutions were selected: CD4 (ready to use), CD8 (ready to use), PD-L1 (1:50), PD-1 (ready to use), TIM-3 (1:200), LAG3 (1:200), Galectin-9 (1:200), CD68 (ready to use), TIGIT (1:500), and Regnase-

1 (1:500). Staining success was confirmed using positive controls in accordance with the manufacturers' instructions. Suitable foreign tissue was used as controls, detailed in Table 1.

Slides were first heated to 72 °C and deparaffinized using an aqueous solution (Ventana EZ Prep, Roche, Basel, CHE). To expose the epitopes for antibody binding, the protein cross-links formed by formalin fixation were reversed by heating slides to 92 °C and incubating them with a buffer (Ventana CC1 or CC2, Roche, Basel, CHE). CC1 is a slightly basic Tris/borate/EDTA buffer (pH 8.4), while CC2 is slightly acidic (pH 6.0). Buffer CC1 was used for CD4, CD8, CD68, PD-L1, and PD-1; buffer CC2 was used for TIM-3, Galectin-9, TIGIT, LAG3, and Regnase-1. Incubation time was 32 minutes for most antibodies, except CD8 (64 minutes), CD68 (24 minutes), and PD-L1 (72 minutes). Endogenous peroxidase activity was blocked by incubation with 3% H_2O_2 (Supplementary Table S1).

Primary antibodies were applied and incubated for 32 minutes, except for CD4 (16 minutes), CD8 (16 minutes), CD68 (12 minutes), PD-L1 (60 minutes), and PD-1 (16 minutes). The antibodies were diluted in a casein-containing solution (Ventana Antibody Diluent, Roche, Basel, CHE) as previously indicated. This was followed by incubation with the secondary antibody (Ventana HQ Universal Linker, Roche, Basel, CHE), a mixture of three polyclonal antibodies conjugated to a synthetic hapten (hydroxy xylene), enhancing sensitivity by amplifying binding sites. Next, the tertiary antibody (Ventana OptiView HRP Multimer, Roche, Basel, CHE), conjugated with horseradish peroxidase (HRP), was applied to bind the haptens.

For chromogenic detection, a 0.2% 3,3'-diaminobenzidine (DAB) solution and 0.04% H_2O_2 were added. The peroxidase catalysed the oxidation of DAB, producing the characteristic brown precipitate. Slides were counterstained with haematoxylin (Roche, Basel, CHE) and subjected to bluing (Roche, Basel, CHE) for 8 minutes each to visualise tissue background and enhance contrast between chromogen and preparation.

Following staining, slides were rinsed under tap water, dehydrated through an ascending alcohol series (1: 70% ethanol, 2: 96% ethanol, 3–4: 100% ethanol, all Th. Geyer, Renningen, GER; 5: isopropanol, Carl Roth, Karlsruhe, GER; 6–7: 100% xylene, J. T. Baker, Avantor, PA, USA), air-dried at room temperature, and mounted using a coverslipping machine (Supplementary Table S2).

**Denomination**	**Manufacturer**
**Devices**	
Immunohistochemistry slide staining system, Ventana Benchmark Ultra	Roche Tissue Diagnostics (Basel, Switzerland)
Microtome, Microm HM355S	Thermo Fisher Scientific (Waltham, Massachusetts, USA)
Microscope, BX50	Olympus (Tokio, Japan)
Fully Automated Glass Coverslipper, CV5030	Leica (Nußloch, Germany)
**Solutions and reagents**	
Ventana Hematoxylin II	Roche Tissue Diagnostics (Basel, Switzerland)
Ventana Bluing Reagent	Roche Tissue Diagnostics (Basel, Switzerland)
Paraffin removal solution (concentrate), Ventana EZ Prep	Roche Tissue Diagnostics (Basel, Switzerland)
Tris based buffer solution (concentrate), Ventana Reaction Buffer Concentrate	Roche Tissue Diagnostics (Basel, Switzerland)
Ventana Liquid Cover Slip	Roche Tissue Diagnostics (Basel, Switzerland)
Ventana Antibody Diluent with Casein	Roche Tissue Diagnostics (Basel, Switzerland)
Ventana OptiView DAB IHC Detection Kit	Roche Tissue Diagnostics (Basel, Switzerland)
Ventana HQ Universal Linker secondary antibody	Roche Tissue Diagnostics (Basel, Switzerland)
Ventana OptiView HRP Multimer tertiary antibody	Roche Tissue Diagnostics (Basel, Switzerland)
Pretreatment buffer solution, Ventana CC1™	Roche Tissue Diagnostics (Basel, Switzerland)
Pretreatment buffer solution, Ventana CC2™	Roche Tissue Diagnostics (Basel, Switzerland)
Xylene based medium, Pertex	Avantor (Pennsylvania, USA)
Ethanol (70%, 96%, 100%)	Th. Geyer (Renningen, Germany)
Isopropanol	Roth, (Karlsruhe, Germany)
Xylol (100%)	J. T. Baker, Avantor (Pennsylvania, USA)
**Materials**	
Superfrost™Plus Adhesion Microscope Slides	Epredia Holdings Ltd (Portsmouth, New Hampshire, USA)
Pipettes	Eppendorf (Hamburg, Germany)
Pipette tips	Sarstedt (Nümbrecht, Germany)

Software	
Nexus	Nexus AG (Donaueschingen, Germany)
AGFA Orbis	Dedalus Healthcare GmbH (Bonn, Germany)
SPSS Statistics Version 29.0.1.0	IBM (Armonk, New York, USA)
Microsoft Excel	Microsoft 365 (Redmond, Washington, USA)
Microsoft Word	Microsoft 365 (Redmond, Washington, USA)

Supplementary Table S1. List of materials used in this study. Devices, solutions and reagents, materials, and software are provided together with the corresponding manufacturers and locations.

Step	Solution	Duration
1	Ethanol 70%	Until slides are free of streaks
2	Ethanol 96%	
3	Ethanol 100%	
4	Ethanol 100%	
5	Isopropyl alcohol	
6	Xylene 100%	
7	Xylene 100%	

Supplementary Table S2. Rehydration protocol used for slide preparation. Slides were sequentially incubated in graded ethanol solutions (70%, 96%, 100%), isopropyl alcohol, and xylene (100%) until free of streaks.

Biomarker	Localisation	n (missing)	Mean (SD)	Minimum	Median	Maximum	Interquartile Range
CD4	I	87 (4)	39.55 (76.83)	0	10	500	27
	E	78 (13)	45.49 (88.12)	0	20	700	45
CD8	I	88 (3)	77.67 (125.27)	0	20	700	85
	E	78 (13)	49.67 (63.08)	0	25	400	65
CD68	I	85 (6)	98.89 (105.08)	0	50	500	120
	E	85 (6)	56.15 (71.72)	0	40	500	65
PD-L1	TPS	89 (2)	6.37 (19.78)	0	0	90	0
	IC	89 (2)	7.48 (16.85)	0	0	80	1
	CPS	89 (2)	11.78 (28.09)	0	0	100	1
PD-1	I	89 (2)	11.81 (24.71)	0	1	150	10

	E	79 (12)	16.04 (36.16)	0	5	250	10
TIM-3	I<br>E	88 (3)<br>87 (4)	1.36 (4.51)<br>0.65 (2.61)	0<br>0	0<br>0	25<br>20	0<br>0
LAG-3	I<br>E	86 (5)<br>85 (6)	0.77 (3.64)<br>0.66 (3.00)	0<br>0	0<br>0	30<br>20	0<br>0
Galectin-9	T<br>I	87 (4)<br>87 (4)	0.0443 (0.17)<br>0.1090 (0.18)	0<br>0	0<br>0	0,9<br>0,8	0<br>0,15
TIGIT	I<br>E	87 (4)<br>87 (4)	10.60 (190.02)<br>9.56 (12.23)	0<br>0	5<br>5	120<br>50	9<br>14
Regnase-1	T	83 (8)	56 (35.78)	0	60	100	70

Supplementary Table S3. Statistical values for the whole cohort. Shown are biomarker expression data including number of evaluable cases (n, with missing values in parentheses), mean with standard deviation (SD), minimum, median, maximum, and interquartile range. CD4 (cluster of differentiation 4), CD8 (cluster of differentiation 8), CD68 (cluster of differentiation 68), PD-L1 (programmed death-ligand 1), PD-1 (programmed cell death protein 1), TIM-3 (T cell immunoglobulin and mucin-domain containing-3), LAG-3 (lymphocyte-activation gene 3), Galectin-9 (β-galactoside-binding lectin 9), TIGIT (T cell immunoreceptor with Ig and ITIM domains), and Regnase-1 (zinc finger CCCH-type containing 12A, ZC3H12A) were analyzed. TPS = tumor proportion score; IC = immune cell score; CPS = combined positive score; T = tumor cells; I = intratumoral; E = extratumoral.

**Biomarker**	**Localisation (missing n)**	**Expression**	**Value (%)**
CD4	I (4)	Positiv<br>Negativ	46 (47.1%)<br>46 (52.9%)
	E (13)	Positiv<br>Negativ	43 (55.1%)<br>35 (44.9%)
CD8	I (3)	Positiv<br>Negativ	48 (54.5%)<br>40 (45.5%)
	E (4)	Positiv<br>Negativ	42 (53.8%)<br>36 (46.2%)
CD68	I (6)	Positiv<br>Negativ	50 (58.8%)<br>35 (41.2%)
	E (6)	Positiv<br>Negativ	45 (52.9%)<br>40 (47.1%)

PD-L1	IC (2)	Positiv<br>Negativ	22 (24.7%)<br>67 (75.3%)
	TPS (2)	Positiv<br>Negativ	14 (15.7%)<br>75 (84.3%)
	CPS (2)	Positiv<br>Negativ	21 (23.6%)<br>68 (76.4%)
PD-1	I (2)	Positiv<br>Negativ	45 (50.6%)<br>40 (49.4%)
	E (12)	Positiv<br>Negativ	42 (53.2%)<br>37 (46.8%)
TIM-3	I (3)	Positiv<br>Negativ	12 (13.6%)<br>76 (86.4%)
	E (4)	Positiv<br>Negativ	8 (9.2%)<br>79 (90.8%)
Galectin-9	T (4)	Positiv<br>Negativ	10 (11.0%)<br>77 (84.6%)
	I (4)	Positiv<br>Negativ	40 (46.0%)<br>47 (54.0%)
LAG-3	I (5)	Positiv<br>Negativ	7 (8.0%)<br>79 (91.9%)
	E (6)	Positiv<br>Negativ	6 (7.1%)<br>79 (92.9%)
TIGIT	I (4)	Positiv<br>Negativ	46 (52.9%)<br>41 (47.1%)
	E (4)	Positiv<br>Negativ	57 (65.5%)<br>30 (34.5%)
Regnase-1	I (8)	Positiv<br>Negativ	47 (56.6%)<br>36 (43.4%)

Supplementary Table S4. Biomarker distribution in the whole cohort. Expression status is shown as positive or negative cases with corresponding percentages. Analyzed biomarkers include CD4 (cluster of differentiation 4), CD8 (cluster of differentiation 8), CD68 (cluster of differentiation 68), PD-L1 (programmed death-ligand 1), PD-1 (programmed cell death protein 1), TIM-3 (T cell immunoglobulin and mucin-domain containing-3), Galectin-9 (β-

galactoside-binding lectin 9), LAG-3 (lymphocyte-activation gene 3), TIGIT (T cell immunoreceptor with Ig and ITIM domains), and Regnase-1 (zinc finger CCCH-type containing 12A, ZC3H12A). TPS = tumor proportion score; IC = immune cell score; CPS = combined positive score; I = intratumoral; E = extratumoral.

Biomarker	Localisation	n (missing)	Mean (SD)	Minimum	Median	Maximum	Interquartile Range
CD4	I	23 (2)	50.57 (111.69)	1	15	500	46
	E	20 (5)	74.20 (153.17)	0	25	700	93
CD8	I	22 (3)	98.77 (178.37)	3	20	700	76
	E	18 (7)	32.28 (24.67)	1	25	70	45
CD68	I	20 (5)	100 (118.24)	5	75	500	159
	E	20 (5)	94.25 (125.26)	5	50	500	128
PD-L1	TPS	23 (2)	7.39 (23.01)	0	0	80	0
	IC	23 (2)	5 (15.74)	0	0	70	0
	CPS	23 (2)	9.57 (28.84)	0	0	100	0
PD-1	I	23 (2)	12.09 (32.77)	0	0	150	5
	E	19 (6)	7.74 (14.61)	0	1	60	10
TIM-3	I	22 (3)	0.73 (2.33)	0	0	10	0
	E	21 (4)	0.95 (2.56)	0	0	10	0
LAG-3	I	22 (3)	1.86 (6.45)	0	0	30	0
	E	21 (4)	0.05 (0.22)	0	0	1	0
Galectin-9	T	21 (4)	0.1595 (0.31)	0	0.9	0	0.1
	I	21 (4)	0.1219 (0.17)	0	0.05	0,5	0.2
TIGIT	I	21 (4)	12.76 (21.31)	0	5	80	12
	E	21 (4)	9.48 (14.49)	0	5	50	10
Regnase-1	T	20 (5)	38.30 (35.61)	0	25	100	70

Supplementary Table S5. Descriptive statistics in the angiosarcoma subgroup. Shown are biomarker expression data including number of evaluable cases (n, with missing values in parentheses), mean with standard deviation (SD), minimum, median, maximum, and interquartile range. Biomarkers analyzed were CD4 (cluster of differentiation 4), CD8 (cluster of differentiation 8), CD68 (cluster of differentiation 68), PD-L1 (programmed death-ligand 1), PD-1 (programmed cell death protein 1), TIM-3 (T cell immunoglobulin and mucin-domain containing-3), LAG-3 (lymphocyte-activation gene 3), Galectin-9 (β-

galactoside-binding lectin 9), TIGIT (T cell immunoreceptor with Ig and ITIM domains), and Regnase-1 (zinc finger CCCH-type containing 12A, ZC3H12A). TPS = tumor proportion score; IC = immune cell score; CPS = combined positive score; I = intratumoral; E = extratumoral.

Biomarker	Localisation	n (missing)	Mean (SD)	Minimum	Median	Maximum	Interquartile Range
CD4	I	31 (2)	22.58 (52.42)	0	3	250	14
	E	28 (5)	15.32 (21.61)	0	5	100	19
CD8	I	33 (0)	66.48 (107,19)	0	15	500	105
	E	30 (3)	34.10 (51.52)	0	10	200	48
CD68	I	32 (1)	60.97 (84.95)	1	27.5	300	40
	E	32 (1)	25.41 (26.91)	0	15	100	31
PD-L1	TPS	33 (0)	0.45 (1.92)	0	0	10	0
	IC	33 (0)	2.88 (10.23)	0	0	50	0
	CPS	33 (0)	2.73 (9.11)	0	0	40	0
PD-1	I	33 (0)	7 (15.50)	0	0	60	5
	E	30 (3)	7.7 (19.45)	0	0	90	5
TIM-3	I	33 (0)	1.45 (4.89)	0	0	20	0
	E	33 (0)	0.61 (3.48)	0	0	20	0
LAG-3	I	32 (1)	0 (0)	0	0	0	0
	E	32 (1)	0 (0)	0	0	0	0
Galectin-9	T	33 (0)	0.0091 (0.05)	0	0	0.3	0
	I	33 (0)	0.0279 (0.07)	0	0.05	0.3	0.01
TIGIT	I	33 (0)	7.58 (12.45)	0	4	60	9
	E	33 (0)	8.3 (11.43)	0	5	50	7
Regnase-1	T	31 (2)	70 (33.47)	0	80	100	50

Supplementary Table S6. **Descriptive statistics in the leiomyosarcoma subgroup.** Biomarker expression data are shown including number of evaluable cases (n, with missing values in parentheses), mean with standard deviation (SD), minimum, median, maximum, and interquartile range. Biomarkers analyzed were CD4 (cluster of differentiation 4) and CD8 (cluster of differentiation 8). C = center; P = periphery; I = intratumoral; E = extratumoral.

Biomarker	Localisation	n (missing)	Mean (SD)	Minimum	Median	Maximum	Interquartile Range
CD4	I<br>E	33 (0)<br>30 (3)	47.82 (65.38)<br>54.50 (57.29)	1<br>5	20<br>35	250<br>250	65<br>50
CD8	I<br>E	33 (0)<br>30 (3)	74.79 (99.25)<br>75.67 (80.11)	0<br>0	30<br>60	400<br>400	85<br>85
CD68	I<br>E	33 (0)<br>33 (0)	135 (104.43)<br>62.88 (39.81)	0<br>15	100<br>50	400<br>200	175<br>60
PD-L1	TPS<br>IC<br>CPS	33 (0)<br>33 (0)<br>33 (0)	11.58 (25.36)<br>13.82 (20.94)<br>22.36 (36.21)	0<br>0<br>0	0<br>0<br>1	90<br>80<br>100	1<br>20<br>40
PD-1	I<br>E	33 (0)<br>30 (3)	16.42 (25.59)<br>29.63 (51.94)	0<br>0	10<br>5	120<br>250	20<br>45
TIM-3	I<br>E	33 (0)<br>33 (0)	1.7 (5.25)<br>0.48 (1.40)	0<br>0	0<br>0	25<br>5	0<br>0
LAG-3	I<br>E	32 (1)<br>32 (1)	0.78 (2.57)<br>1.72 (4.75)	0<br>0	0<br>0	10<br>20	0<br>0
Galectin-9	T<br>I	33 (0)<br>33 (0)	0.006 (0.02)<br>0.1818 (0.22)	0<br>0	0<br>0.1	0.1<br>0.8	0<br>0.3
TIGIT	I<br>P	33 (0)<br>33 (0)	12.24 (22.77)<br>10.88 (11.70)	0<br>0	5<br>5	120<br>40	17<br>18
Regnase-1	T	32 (1)	53.50 (33.46)	0	65	100	68

Supplementary Table S7. Descriptive statistics in the undifferentiated pleomorphic sarcoma (UPS) subgroup. Shown are biomarker expression data including number of evaluable cases (n, with missing values in parentheses), mean with standard deviation (SD), minimum, median, maximum, and interquartile range. Biomarkers analyzed were CD4 (cluster of differentiation 4), CD8 (cluster of differentiation 8), CD68 (cluster of differentiation 68), PD-L1 (programmed death-ligand 1), PD-1 (programmed cell death protein 1), TIM-3 (T cell immunoglobulin and mucin-domain containing-3), LAG-3 (lymphocyte-activation gene 3), Galectin-9 (β-galactoside-binding lectin 9), TIGIT (T cell immunoreceptor with Ig and ITIM domains), and Regnase-1 (zinc finger CCCH-type containing 12A, ZC3H12A). TPS = tumor proportion score; IC = immune cell score; CPS = combined positive score; T = tumor cells; C = center; P = periphery; I = intratumoral; E = extratumoral.

AS	CD4<br>I	CD4<br>E	CD8<br>I	CD8<br>E	CD68<br>I	CD68<br>E	PD-L1<br>TPS	PD-L1<br>IC	PD-L1<br>CPS	PD-1<br>I	PD-1<br>E	TIM-3<br>I	TIM-3<br>E	LAG3<br>I	LAG3<br>E	Gal-9<br>Tumor	Gal-9<br>Histios	TIGIT<br>I	TIGIT<br>E
**CD4<br>E**	0.65<br>(0.0018)<br>20																		
**CD8<br>I**	0.55<br>(0.0082)<br>22	0.60<br>(0.0062)<br>19																	
**CD8<br>E**	0.61<br>(0.0069)<br>18	0.78<br>(0.0001)<br>18	0.68<br>(0.0018)<br>18																
**CD68<br>I**	0.40<br>(0.0834)<br>20	0.31<br>(0.2041)<br>18	0.74<br>(0.0002)<br>19	0.57<br>(0.0226)<br>16															
**CD68<br>E**	0.43<br>(0.0570)<br>20	0.38<br>(0.1199)<br>18	0.76<br>(0.0001)<br>19	0.51<br>(0.0417)<br>16	0.94<br>(0.0000)<br>20														
**PD-L1<br>TPS**	-0.02<br>(0.9266)<br>23	0.24<br>(0.3013)<br>20	0.58<br>(0.0047)<br>22	0.32<br>(0.1974)<br>18	0.48<br>(0.0320)<br>20	0.48<br>(0.0310)<br>20													
**PD-L1<br>IC**	-0.03<br>(0.8946)<br>23	0.24<br>(0.3013)<br>20	0.58<br>(0.0046)<br>22	0.32<br>(0.1974)<br>18	0.48<br>(0.0335)<br>20	0.48<br>(0.0316)<br>20	1<br>(0.000)<br>23												
**PD-L1<br>CPS**	-0.02<br>(0.9266)<br>23	0.24<br>(0.3013)<br>20	0.58<br>(0.0047)<br>22	0.32<br>(0.1974)<br>18	0.48<br>(0.0320)<br>20	0.48<br>(0.0310)<br>20	1.000<br>23	1<br>(0.000)<br>23											
**PD-1<br>I**	0.45<br>(0.0305)<br>23	0.50<br>(0.0261)<br>20	0.61<br>(0.0029)<br>22	0.70<br>(0.0011)<br>18	0.57<br>(0.0081)<br>20	0.54<br>(0.0135)<br>20	0.53<br>(0.0094)<br>23	0.53<br>(0.0096)<br>23	0.53<br>(0,0094)<br>23										
**PD-1<br>E**	0.61<br>(0.0057)<br>19	0.61<br>(0.0059)<br>19	0.68<br>(0.0018)<br>18	0.78<br>(0.0001)<br>18	0.61<br>(0.0092)<br>17	0.63<br>(0.0067)<br>17	0.41<br>(0.0822)<br>19	0.41<br>(0,0822)<br>19	0.41<br>(0.0822)<br>19	0.88<br>(0.0000)<br>19									
**TIM-3<br>I**	0.33<br>(0.1282)<br>22	-0.17<br>(0.4905)<br>19	0.13<br>(0.5736)<br>21	0.08<br>(0.7620)<br>17	0.18<br>(0.4565)<br>20	0.09<br>(0.7130)<br>20	0.28<br>(0.2057)<br>22	0.26<br>(0,2398)<br>22	0.28<br>(0.2057)<br>22	0.32<br>(0.1437)<br>22	0.05<br>(0.8418)<br>18								
**TIM-3<br>E**	0.20<br>(0.3835)<br>21	-0.12<br>(0.6164)<br>19	-0.13<br>(0.5716)<br>20	0.09<br>(0.7253)<br>17	-0.25<br>(0.2880)<br>19	-0.38<br>(0.1036)<br>19	-0.13<br>(0.5686)<br>21	-0.13<br>(0,5686)<br>21	-0.13<br>(0.5686)<br>21	-0.01<br>(0.9496)<br>21	-0.10<br>(0.7063)<br>18	0.75<br>(0.0001)<br>21							
**LAG3<br>I**	0.06<br>(0.7985)<br>22	0.35<br>(0.1396)<br>19	0.42<br>(0.0552)<br>21	0.45<br>(0.0725)<br>17	0.36<br>(0.1219)<br>20	0.34<br>(0.1445)<br>20	0.58<br>(0.0051)<br>22	0.57<br>(0,0054)<br>22	0.58<br>(0.0051)<br>22	0.27<br>(0.2228)<br>22	0.16<br>(0.5342)<br>18	0.24<br>(0.2897)<br>22	0.28<br>(0.2223)<br>21						
**LAG3<br>E**	-0.04<br>(0.8732)<br>21	0.02<br>(0.9301)<br>19	-0.08<br>(0.7372)<br>20	0.03<br>(0.9219)<br>17	-0.30<br>(0.2025)<br>19	-0.35<br>(0.1451)<br>19	-0.07<br>(0.7550)<br>21	-0.07<br>(0,7550)<br>21	-0.07<br>(0.7550)<br>21	-0.16<br>(0.4629)<br>21	-0.22<br>(0.3682)<br>18	-0.07<br>(0.7550)<br>21	0.61<br>(0.0035)<br>21	0.58<br>(0.0062)<br>21					
**Gal-9<br>Tumor**	-0.15<br>(0.5284)<br>21	-0.12<br>(0.6257)<br>19	0.07<br>(0.7845)<br>20	-0.10<br>(0.6969)<br>17	0.12<br>(0.6184)<br>20	0.07<br>(0.7827)<br>20	0.39<br>(0.0794)<br>21	0.39<br>(0,0796)<br>21	0.39<br>(0.0794)<br>21	0.23<br>(0.3185)<br>21	-0.05<br>(0.8475)<br>18	0.49<br>(0.0247)<br>21	0.54<br>(0.0131)<br>20	0.53<br>(0.0141)<br>21	0.37<br>(0.1103)<br>20				
**Gal-9<br>Histios**	-0.12<br>(0.5937)<br>21	0.1 (0.6932)<br>19	-0.28<br>(0.2354)<br>20	-0.16<br>(0.5310)<br>17	-0.29<br>(0.2145)<br>20	-0.32<br>(0.1654)<br>20	-0.30<br>(0.1885)<br>21	-0.30<br>(0,1804)<br>21	-0.30<br>(0.1885)<br>21	-0.30<br>(0.1845)<br>21	-0.47<br>(0.0520)<br>18	-0.09<br>(0.6850)<br>21	0.06<br>(0.8166)<br>20	0.11<br>(0.6402)<br>21	0.21<br>(0.3761)<br>20	0.04<br>(0.8797)<br>21			
**TIGIT<br>I**	0.32<br>(0.1518)<br>21	0.01<br>(0.9723)<br>19	0.29<br>(0.2157)<br>20	0.13<br>(0.6246)<br>18	0.57<br>(0.0086)<br>20	0.48<br>(0.0323)<br>20	0.27<br>(0.2362)<br>21	0.26<br>(0,2629)<br>21	0.27<br>(0.2362)<br>21	0.57<br>(0.0075)<br>21	0.43<br>(0.0787)<br>18	0.35<br>(0.1246)<br>21	-0.02<br>(0.9259)<br>20	0.07<br>(0.7794)<br>21	-0.24<br>(0.3012)<br>20	0.10<br>(0.6800)<br>21	-0.33<br>(0.1488)<br>21		
**TIGIT<br>E**	0.47<br>(0.0305)<br>21	0.40<br>(0.0890)<br>19	0.29<br>(0.2119)<br>20	0.49<br>(0.0479)<br>17	0.43<br>(0.0561)<br>20	0.47<br>(0.0352)<br>20	0.22<br>(0.3492)<br>21	0.21<br>(0,3688)<br>21	0.22<br>(0.3492)<br>21	0.27<br>(0.2305)<br>21	0.36<br>(0.1482)<br>18	0.24<br>(0.3027)<br>21	-0.03<br>(0.9169)<br>20	0.24<br>(0.2998)<br>21	-0.14<br>(0.5466)<br>20	0.04<br>(0.8819)<br>21	-0.12<br>(0.5973)<br>21	0.48<br>(0.0261)<br>21	
**Regnase-1<br>Tumor**	0.11<br>(0.6573)<br>20	0.11<br>(0.6758)<br>18	0.26<br>(0.2806)<br>19	0.27<br>(0.3073)<br>16	0.19<br>(0.4242)<br>20	0.15<br>(0.5409)<br>20	0.30<br>(0.2046)<br>20	0.29<br>(0,2130)<br>20	0.30<br>(0.2046)<br>20	0.44<br>(0.0511)<br>20	0.30<br>(0.2364)<br>17	0.45<br>(0.0441)<br>20	0.14<br>(0.5790)<br>19	0.42<br>(0.0675)<br>20	-0.15<br>(0.5348)<br>19	0.26<br>(0.2637)<br>20	0.16<br>(0.4886)<br>20	0.11<br>(0.6393)<br>20	0.03<br>(0.8865)<br>20

Supplementary Table S8. Biomarker correlation in the angiosarcoma subgroup. Shown are correlation coefficients with corresponding p-values for all analyzed biomarkers. Biomarkers include CD4 (cluster of differentiation 4), CD8 (cluster of differentiation 8), CD68 (cluster of differentiation 68), PD-L1 (programmed death-ligand 1), PD-1 (programmed cell death protein 1), TIM-3 (T cell immunoglobulin and mucin-domain containing-3), LAG-3 (lymphocyte-activation gene 3), Galectin-9 (β-galactoside-binding lectin 9), TIGIT (T cell immunoreceptor with Ig and ITIM domains), and Regnase-1 (zinc finger CCCH-type containing 12A, ZC3H12A). TPS = tumor proportion score; IC = immune cell score; CPS = combined positive score; T = tumor cells; C = center; P = periphery; I = intratumoral; E = extratumoral.

**LMS**	**CD4<br>I**	**CD4<br>E**	**CD8<br>I**	**CD8<br>E**	**CD68<br>I**	**CD68<br>E**	**PD-L1<br>TPS**	**PD-L1<br>IC**	**PD-L1<br>CPS**	**PD-1<br>I**	**PD-1<br>E**	**TIM-3<br>I**	**TIM-3<br>E**	**Gal-9<br>Tumor**	**Gal-9<br>Histios**	**TIGIT<br>I**	**TIGIT<br>E**
**CD4<br>I**	0.57<br>(0.0017)<br>28																
**CD8<br>I**	0.57<br>(0.0007)<br>31	0.46<br>(0.0129)<br>28															
**CD8<br>E**	0.18<br>(0.3548)<br>28	0.49<br>(0.0082)<br>28	0.74<br>(0.0000)<br>30														
**CD68<br>I**	0.34<br>(0.0639)<br>30	0.32<br>(0.1006)<br>27	0.41<br>(0.0184)<br>32	0.18<br>(0.3419)<br>29													
**CD68<br>E**	0.32<br>(0.0872)<br>30	0.29<br>(0.1450)<br>27	0.50<br>(0.0035)<br>32	0.39<br>(0.0348)<br>29	0.67<br>(0.0000)<br>32												
**PD-L1<br>TPS**	0.27<br>(0.1462)<br>31	0.33<br>(0.0861)<br>28	0.38<br>(0.0307)<br>33	0.40<br>(0.0291)<br>30	0.39<br>(0.0285)<br>32	0.31<br>(0.0821)<br>32											
**PD-L1 IC**	0.35<br>(0.0566)<br>31	0.33<br>(0.0861)<br>28	0.41<br>(0.0179)<br>33	0.40<br>(0.0291)<br>30	0.48<br>(0.0056)<br>32	0.42<br>(0.0171)<br>32	0.78<br>(0.0000)<br>33										
**PD-L1<br>CPS**	0.35<br>(0.0548)<br>31	0.33<br>(0.0861)<br>28	0.41<br>(0.0171)<br>33	0.40<br>(0.0291)<br>30	0.48<br>(0.0055)<br>32	0.42<br>(0.0171)<br>32	0.80<br>(0.0000)<br>33	1<br>(0.0000)<br>33									
**PD-1<br>I**	0.33<br>(0.0694)<br>31	0.48<br>(0.0096)<br>28	0.33<br>(0.0573)<br>33	0.20<br>(0.2850)<br>30	0.25<br>(0.1678)<br>32	0.06<br>(0.7288)<br>32	0.11<br>(0.5606)<br>33	0.17<br>(0.3479)<br>33	0.17<br>(0.3320)<br>33								
**PD-1<br>E**	0.11<br>(0.5754)<br>28	0.21<br>(0.2804)<br>28	0.34<br>(0.0642)<br>30	0.45<br>(0.0137)<br>30	0.29<br>(0.1292)<br>29	0.33<br>(0.0819)<br>29	0.44<br>(0.0150)<br>30	0.44<br>(0.0150)<br>30	0.44<br>(0.0150)<br>30	0.46<br>(0.0110)<br>30							
**TIM-3<br>I**	0.49<br>(0.0049)<br>31	0.46<br>(0.0129)<br>28	0.56<br>(0.0007)<br>33	0.52<br>(0.0032)<br>30	0.44<br>(0.0113)<br>32	0.36<br>(0.046)<br>32	0.70<br>(0.0000)<br>33	0.52<br>(0.0020)<br>33	0.54<br>(0.0012)<br>33	0.41<br>(0.0182)<br>33	0.47<br>(0.0095)<br>30						
**TIM-3<br>E**	0.27<br>(0.1462)<br>31	0.33<br>(0.0861)<br>28	0.24<br>(0.1710)<br>33	0.26<br>(0.1631)<br>30	0.29<br>(0.1032)<br>32	0.26<br>(0.1432)<br>32	0.72<br>(0.0000)<br>33	0.56<br>(0.0007)<br>33	0.60<br>(0.0000)<br>33	0.29<br>(0.1060)<br>33	0.34<br>(0.0697)<br>30	0.51<br>(0.0026)<br>33					
**Gal-9<br>Tumor**	0.31<br>(0.0916)<br>31	0.22<br>(0.2603)<br>28	0.24<br>(0.1710)<br>33	0.26<br>(0.1631)<br>30	0.29<br>(0.1032)<br>32	0.30<br>(0.0909)<br>32	-0.05<br>(0.8041)<br>33	-0.06<br>(0.7576)<br>33	-0.06<br>(0.7576)<br>33	0.32<br>(0.0721)<br>33	0.36<br>(0.0502)<br>30	0.51<br>(0.0026)<br>33	-0.03<br>(0.8620)<br>33				

**Gal-9<br>Histios**	0.13<br>(0.4800)<br>31	0.28<br>(0.1471)<br>28	0.38<br>(0.0312)<br>33	0.41<br>(0.0247)<br>30	0.32<br>(0.0768)<br>32	0.54<br>(0.0014)<br>32	0.21<br>(0.2517)<br>33	0.35<br>(0.0494)<br>33	0.35<br>(0.0473)<br>33	0.27<br>(0.1239)<br>33	0.25<br>(0.1801)<br>30	0.33<br>(0.0622)<br>33	0.37<br>(0.0338)<br>33	0.40<br>(0.0228)<br>33			
**TIGIT<br>I**	0.23<br>(0.2212)<br>31	0.18<br>(0.3632)<br>28	0.16<br>(0.3905)<br>33	-0.16<br>(0.3844)<br>30	0.06<br>(0.7308)<br>32	-0.07<br>(0.7149)<br>32	-0.22<br>(0.2197)<br>33	-0.22<br>(0.2226)<br>33	-0.22<br>(0.2096)<br>33	0.41<br>(0.0175)<br>33	-0.07<br>(0.7217)<br>30	0.11<br>(0.5294)<br>33	-0.24<br>(0.1884)<br>33	0.28<br>(0.1121)<br>33	0.01<br>(0.9762)<br>33		
**TIGIT<br>E**	-0.08<br>(0.6524)<br>31	0.03<br>(0.8825)<br>28	-0.13<br>(0.4817)<br>33	-0.26<br>(0.1610)<br>30	0.34<br>(0.0582)<br>32	0.25<br>(0.1763)<br>32	-0.18<br>(0.3053)<br>33	-0.14<br>(0.4302)<br>33	-0.15<br>(0.3993)<br>33	0.13<br>(0.4800)<br>33	-0.14<br>(0.4770)<br>30	-0.05<br>(0.7675)<br>33	-0.26<br>(0.1476)<br>33	0.27<br>(0.1327)<br>33	0.09<br>(0.6159)<br>33	0.44<br>(0.0096)<br>33	
**Regnase-1<br>Tumor**	-0.16<br>(0.3924)<br>30	-0.45<br>(0.0181)<br>27	-0.36<br>(0.0468)<br>31	-0.36<br>(0.0579)<br>28	-0.45<br>(0.0107)<br>31	-0.45<br>(0.0120)<br>31	-0.27<br>(0.1352)<br>31	-0.35<br>(0.0554)<br>31	-0.35<br>(0.0533)<br>31	-0.45<br>(0.0120)<br>31	-0.17<br>(0.3778)<br>28	-0.45<br>(0.0107)<br>31	-0.27<br>(0.1352)<br>31	-0.31<br>(0.0940)<br>31	-0.4859<br>(0.0056)<br>31	-0.26<br>(0.1651)<br>31	-0.12<br>(0.5040)<br>31

Supplementary Table S9. Biomarker correlation in the leiomyosarcoma (LMS) subgroup. Shown are correlation coefficients with corresponding p-values for all analyzed biomarkers. Biomarkers include CD4 (cluster of differentiation 4), CD8 (cluster of differentiation 8), CD68 (cluster of differentiation 68), PD-L1 (programmed death-ligand 1), PD-1 (programmed cell death protein 1), TIM-3 (T cell immunoglobulin and mucin-domain containing-3), LAG-3 (lymphocyte-activation gene 3), Galectin-9 (β-galactoside-binding lectin 9), TIGIT (T cell immunoreceptor with Ig and ITIM domains), and Regnase-1 (zinc finger CCCH-type containing 12A, ZC3H12A). TPS = tumor proportion score; IC = immune cell score; CPS = combined positive score; T = tumor cells; C = center; P = periphery; I = intratumoral; E = extratumoral.

**UPS**	**CD4<br>I**	**CD4<br>E**	**CD8<br>I**	**CD8<br>E**	**CD68<br>I**	**CD68<br>E**	**PD-L1<br>TPS**	**PD-L1<br>IC**	**PD-L1<br>CPS**	**PD-1<br>I**	**PD-1<br>E**	**TIM-3<br>I**	**TIM-3<br>E**	**LAG3<br>I**	**LAG3<br>E**	**Gal-9<br>Tumor**	**Gal-9<br>Histios**	**TIGIT<br>I**	**TIGIT<br>E**
**CD4<br>E**	0.28<br>(0.1334)<br>30																		
**CD8<br>I**	0.65<br>(0.0000)<br>33	0.32<br>(0.0813)<br>30																	
**CD8<br>E**	0.04<br>(0.8405)<br>30	0.46<br>(0.0109)<br>30	0.31<br>(0.0945)<br>30																
**CD68<br>I**	0.26<br>(0.1425)<br>33	0.10<br>(0.5978)<br>30	0.25<br>(0.1578)<br>33	0.08<br>(0.6686)<br>30															
**CD68<br>E**	-0.07<br>(0.7134)<br>33	0.02<br>(0.9363)<br>30	0.10<br>(0.5902)<br>33	0.30<br>(0.1046)<br>30	0.66<br>(0.0000)<br>33														
**PD-L1<br>TPS**	-0.03<br>(0.8733)<br>33	0.34<br>(0.0673)<br>30	0.27<br>(0.1231)<br>33	0.35<br>(0.0607)<br>30	0.16<br>(0.3638)<br>33	0.38<br>(0.0303)<br>33													
**PD-L1<br>IC**	0.49<br>(0.0042)<br>33	0.40<br>(0.0277)<br>30	0.61<br>(0.0000)<br>33	0.59<br>(0.0005)<br>30	0.29<br>(0.0980)<br>33	0.33<br>(0.0601)<br>33	0.47<br>(0.0062)												
**PD-L1<br>CPS**	0.38<br>(0.0293)<br>33	0.43<br>(0.0189)<br>30	0.58<br>(0.000)<br>33	0.57<br>(0.0011)<br>30	0.28<br>(0.1102)<br>33	0.39<br>(0.0231)<br>33	0.70<br>(0.0000)<br>33	0.93<br>(0.0000)<br>33											
**PD-1<br>I**	0.58<br>(0.0000)<br>33	0.49<br>(0.0062)<br>30	0.62<br>(0.0000)	0.18<br>(0.3402)<br>30	-0.12<br>(0.4965)<br>33	-0.25<br>(0.1663)<br>33	0.13<br>(0.4778)<br>33	0.26<br>(0.1499)<br>33	0.29<br>(0.1013)<br>33										
**PD-1<br>E**	-0.02<br>(0.9097)<br>30	0.64<br>(0.0000)<br>30	0.09<br>(0.6490)<br>30	0.55<br>(0.0017)<br>30	-0.06<br>(0.7377)<br>30	0.08<br>(0.6829)<br>30	0.16<br>(0.4016)<br>30	0.30<br>(0.1108)<br>30	0.34<br>(0.0699)<br>30	0.41<br>(0.0253)<br>30									

**TIM-3<br>I**	-0.02<br>(0.9069)<br>33	0.05<br>(0.7752)<br>30	0.29<br>(0.1003)<br>33	0.10<br>(0.6173)<br>30	0.30<br>(0.0934)<br>33	0<br>(0.9856)<br>33	0.13<br>(0.4618)<br>33	0.14<br>(0.4441)<br>33	0.14<br>(0.4521)<br>33	0.01<br>(0.9648)<br>33	-0.14<br>(0.4719)<br>30								
**TIM-3<br>E**	-0.15<br>(0.4164)<br>33	-0.09<br>(0.6245)<br>30	0.14<br>(0.4345)<br>33	0.15<br>(0.4457)<br>30	0.04<br>(0.8352)<br>33	-0.02<br>(0.9319)<br>33	-0.02<br>(0.9107)<br>33	0.0235<br>(0.8966)<br>33	0.01<br>(0.9427)<br>33	-0.14<br>(0.4538)<br>33	-0.10<br>(0.6046)<br>30	0.62<br>(0.0001)							
**LAG3<br>I**	0.07<br>(0.7207)<br>32	0.14<br>(0.4584)<br>29	0.47<br>(0.0073)<br>32	0.46<br>(0.0112)<br>29	0.25<br>(0.1719)<br>32	0.45<br>(0.0107)<br>32	0.37<br>(0.0381)<br>32	0.37<br>(0.0392)<br>32	0.44<br>(0.0114)<br>32	0.31<br>(0.0829)<br>32	0.22<br>(0.2504)<br>29	0.20<br>(0.2652)<br>32	0.18<br>(0.3222)<br>32						
**LAG3<br>E**	-0.04<br>(0.8315)<br>32	0.33<br>(0.0823)<br>29	0.26<br>(0.1439)<br>32	0.57<br>(0.0014)<br>29	0.19<br>(0.3021)<br>32	0.32<br>(0.0761)<br>32	0.36<br>(0.0406)<br>32	0.43<br>(0.0144)<br>32	0.48<br>(0.0059)<br>32	0.19<br>(0.3105)<br>32	0.36<br>(0.0575)<br>29	0.10<br>(0.5965)<br>32	0.09<br>(0.6347)<br>32	0.80<br>(0.0000)<br>32					
**Gal-9<br>Tumor**	0.07<br>(0.6828)<br>33	-0.01<br>(0.9675)<br>30	0.34<br>(0.0517)<br>33	0.29<br>(0.1151)<br>30	-0.13<br>(0.4797)<br>33	0.12<br>(0.5250)<br>33	0.20<br>(0.2754)<br>33	0.18<br>(0.3168)<br>33	0.29<br>(0.1046)<br>33	0.28<br>(0.1099)<br>33	0.21<br>(0.2586)<br>33	-0.11<br>(0.5539)<br>33	0.32<br>(0.0717)<br>33	0.37<br>(0.0353)<br>32	0.24<br>(0.1796)<br>32				
**Gal-9<br>Histios**	0.19<br>(0.2932)<br>33	0.33<br>(0.0716)<br>30	0.30<br>(0.0862)<br>33	0.04<br>(0.8169)<br>30	0.30<br>(0.0929)<br>33	0.12<br>(0.5250)<br>33	0.04<br>(0.8254)<br>33	0.01<br>(0.9716)<br>33	0.03<br>(0.8897)<br>33	0.33<br>(0.0579)<br>33	0.24<br>(0.2117)<br>30	0.18<br>(0.3062)<br>33	0.23<br>(0.2060)<br>33	0.03<br>(0.8708)<br>32	0.04<br>(0.8303)<br>32	0.26<br>(0.1513)<br>33			
**TIGIT<br>I**	0.34<br>(0.0510)<br>33	0.43<br>(0.0189)<br>30	0.59<br>(0.0009)<br>33	0.07<br>(0.7032)<br>30	-0.07<br>(0.7040)<br>33	-0.27<br>(0.1270)<br>33	0.16<br>(0.3905)<br>33	0.31<br>(0.0787)<br>33	0.32<br>(0.0671)<br>33	0.55<br>(0.0008)<br>33	0.24<br>(0.2034)<br>30	0.27<br>(0.1278)<br>33	0.09<br>(0.6170)<br>33	0.07<br>(0.7201)<br>32	0.03<br>(0.8886)<br>32	0.17<br>(0.3481)<br>33	0.38<br>(0.0305)<br>33		
**TIGIT<br>E**	0.36<br>(0.0383)<br>33	0.64<br>(0.0000)<br>30	0.49<br>(0.0040)<br>33	0.43<br>(0.0186)<br>30	0.05<br>(0.7811)<br>33	-0.11<br>(0.5311)<br>33	0.33<br>(0.0628)<br>33	0.54<br>(0.0012)<br>33	0.52<br>(0.0019)<br>33	0.42<br>(0.0161)<br>33	0.28<br>(0.1294)<br>30	0.25<br>(0.1572)<br>33	-0.03<br>(0.8895)<br>33	0.15<br>(0.4287)<br>32	0.12<br>(0.5019)<br>32	0.00 (1.0)<br>33	0.16<br>(0.3806)<br>33	0.58<br>(0.0004)<br>33	
**Regnase-<br>1 Tumor**	-0.11<br>(0.5612)<br>32	-0.01<br>(0.9604)<br>29	-0.14<br>(0.4460)<br>32	0.32<br>(0.0951)<br>29	0.11<br>(0.5547)<br>32	-0.01<br>(0.9432)<br>32	-0.20<br>(0.2719)<br>32	-0.11<br>(0.5474)<br>32	-0.19<br>(0.2988)<br>32	-0.23<br>(0.2142)<br>32	-0.03<br>(0.8842)<br>29	-0.11<br>(0.5619)<br>32	0.07<br>(0.6963)<br>32	-0.14<br>(0.4446)<br>31	0.03<br>(0.8566)<br>31	-0.21<br>(0.2450)<br>32	0.08<br>(0.6511)<br>32	-0.19<br>(0.2919)<br>32	-0.02<br>(0.8976)<br>32

Supplementary Table S10. Biomarker correlation in the undifferentiated pleomorphic sarcoma (UPS) subgroup. Shown are correlation coefficients with corresponding p-values for all analyzed biomarkers. Biomarkers include CD4 (cluster of differentiation 4), CD8 (cluster of differentiation 8), CD68 (cluster of differentiation 68), PD-L1 (programmed death-ligand 1), PD-1 (programmed cell death protein 1), TIM-3 (T cell immunoglobulin and mucin-domain containing-3), LAG-3 (lymphocyte-activation gene 3), Galectin-9 (β-galactoside-binding lectin 9), TIGIT (T cell immunoreceptor with Ig and ITIM domains), and Regnase-1 (zinc finger CCCH-type containing 12A, ZC3H12A). TPS = tumor proportion score; IC = immune cell score; CPS = combined positive score; T = tumor cells; C = center; P = periphery; I = intratumoral; E = extratumoral

Cohort	Spearman’s rho (r) for CD68-positive TAMs and Regnase-1	p-value
Total cohort (all subtypes)	–0.157	0.157
Total cohort (UPS subtype)	+0.048	0.796

Supplementary Table S11. Correlation between CD68-positive TAMs and Regnase-1 expression in the predefined patient cohorts Shown are coefficients with corresponding p-values for all analyzed biomarkers. No significant correlations were found.

Cohort / Analysis	Marker	Model	HR	95% CI	p-value	Required events (80% power)
Total cohort	G3 grading	Univariable Cox	1.80	-	0.0371	91
Total cohort	Metastatic disease (M1)	Univariable Cox	1.90	-	0.0264	77
Total cohort	Non-R0 resection	Univariable Cox	2.50	-	0.0013	38
Total cohort	CD68+ TAM high	Univariable Cox	2.00	1.1–3.7	0.0325	66
UPS cohort	Regnase-1 high	Univariable Cox	0.32	0.11–0.92	0.0343	25

Total cohort	Higher grading	Multivariable Cox	2.22	1.21–4.07	0.0102	50
Total cohort	Regnase-1 positive	Multivariable Cox	0.67	0.51–0.90	0.0074	196
UPS cohort	Regnase-1 high	Multivariable Cox	0.39	0.16–0.97	0.0443	36
Surgery + irradiation	Regnase-1 high	Univariable Cox	2.80	1.0–7.69	0.0491	30
TCGA-SARC (OS)	ZC3H12A high	Univariable Cox	0.58	0.37–0.91	0.0170	106

Supplementary Table S12. Post hoc power sensitivity analysis using the Schoenfeld approximation for Cox/log-rank comparisons across primary and TCGA-SARC cohorts. For each statistically significant dichotomized survival association, the observed HR, corresponding 95% CI, p-value, and the estimated number of events required to detect the effect with 80% power ($\alpha = 0.05$; $p = 0.5$) are shown. For HR < 1, reciprocal values were used for calculation. Required event numbers varied substantially, reflecting differences in effect size and cohort composition, with lower event requirements for larger effects and higher requirements for more moderate associations. Abbreviations: UPS, undifferentiated pleomorphic sarcoma; TAMs, tumour-associated macrophages; OS, overall survival.

A

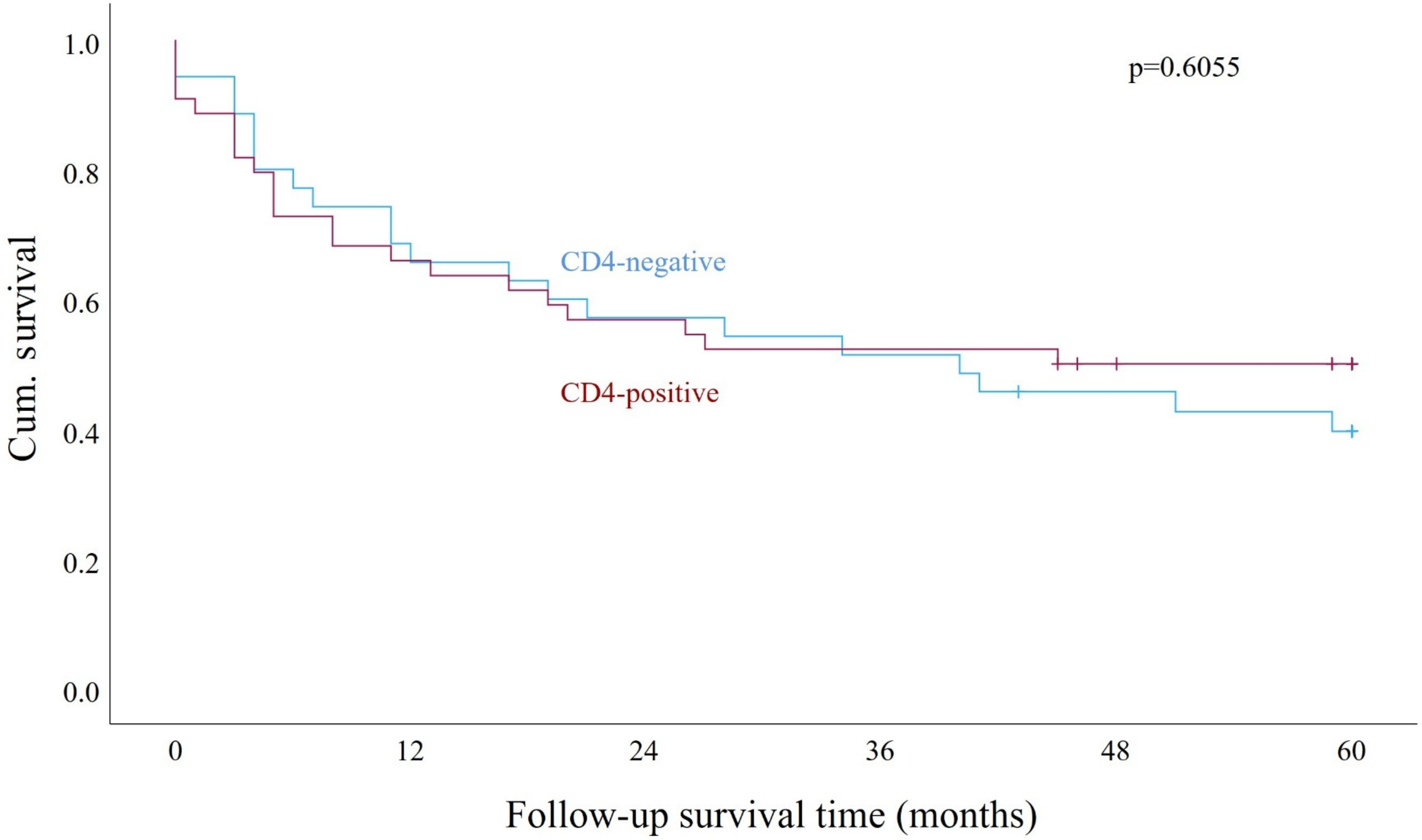
p=0.6055
CD4-negative
CD4-positive
Cum. survival
1.0
0.8
0.6
0.4
0.2
0.0
0
12
24
36
48
60
Follow-up survival time (months)

B

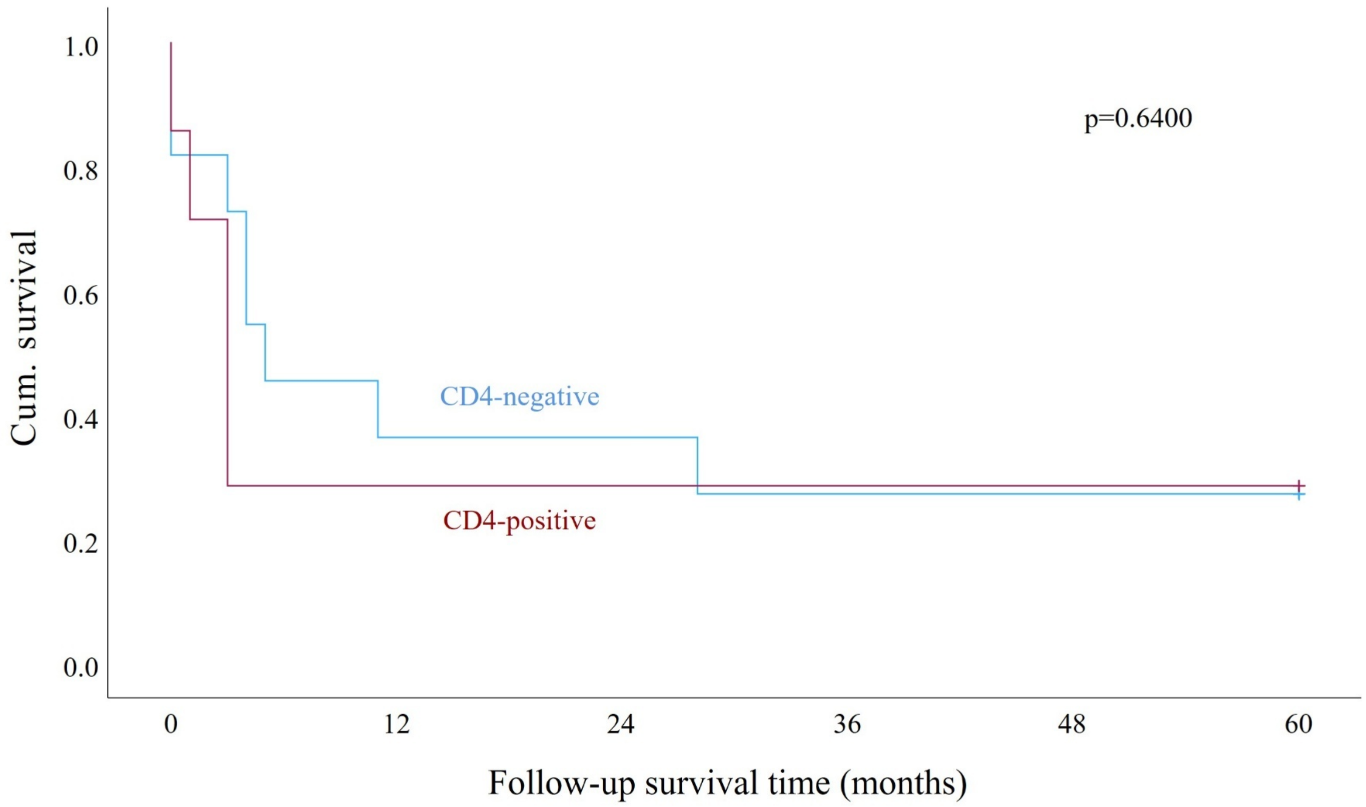
p=0.6400
Cum. survival
1.0
0.8
0.6
0.4
0.2
0.0
CD4-negative
CD4-positive
0
12
24
36
48
60
Follow-up survival time (months)

C

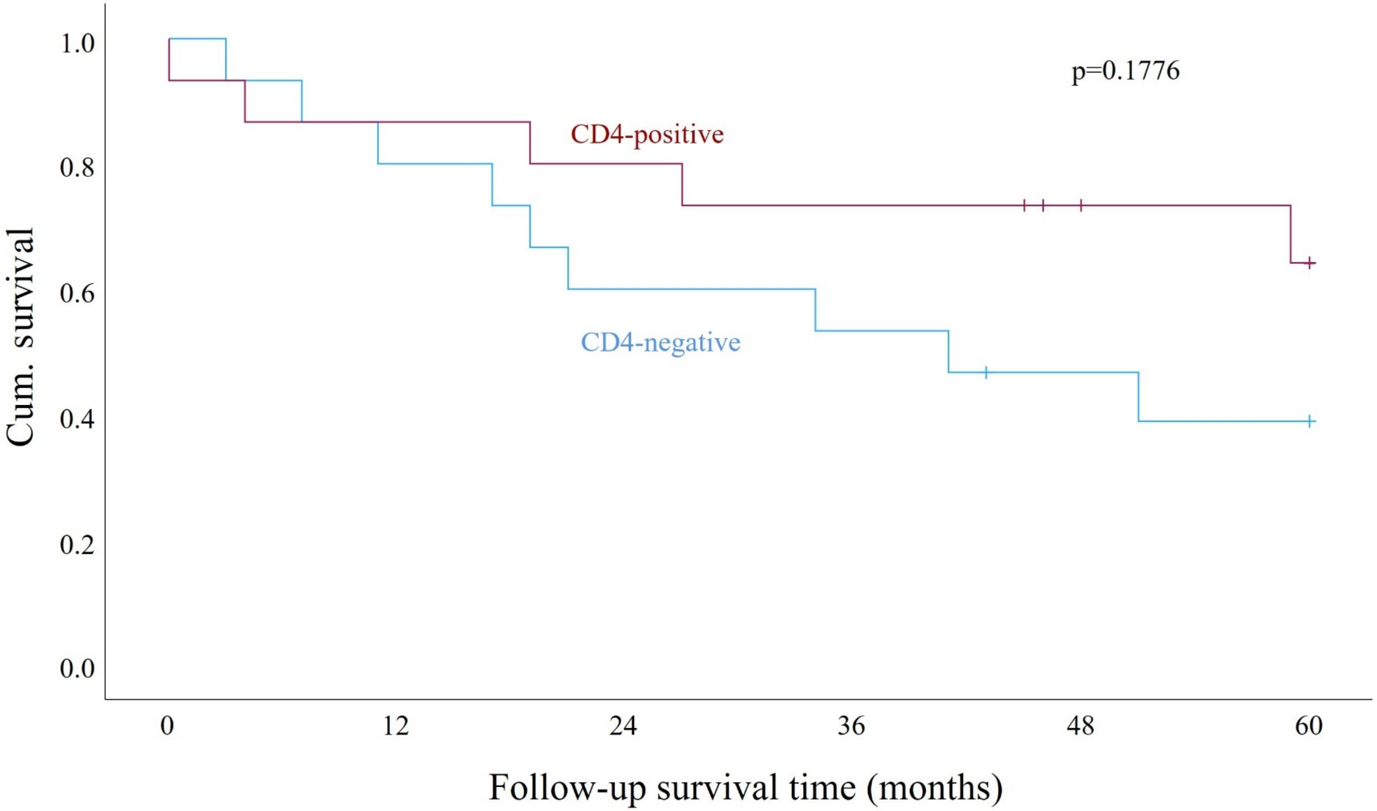

p=0.1776
CD4-positive
CD4-negative
Cum. survival
1.0
0.8
0.6
0.4
0.2
0.0
0
12
24
36
48
60
Follow-up survival time (months)

D

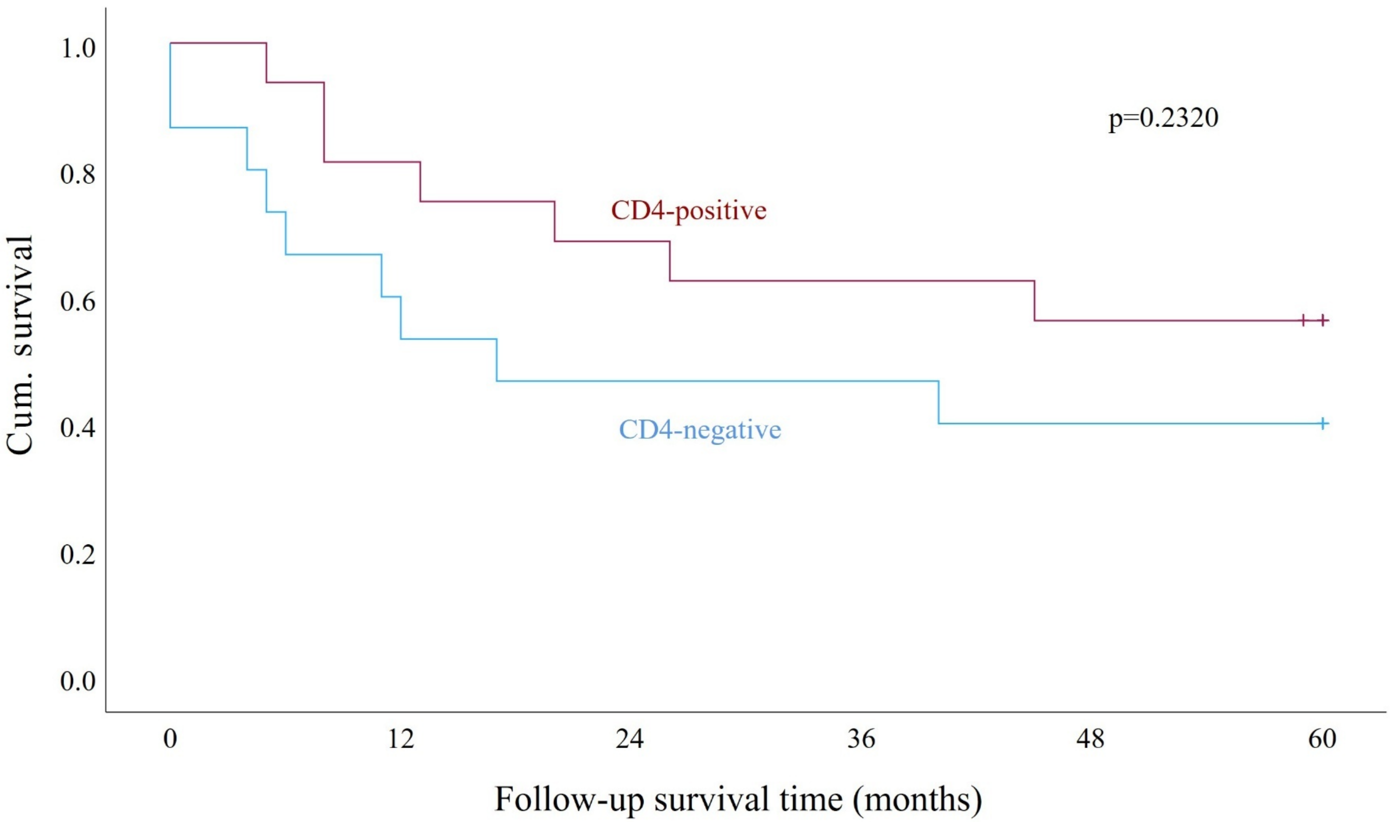

Figure S1A-D: Kaplan-Meier analysis for CD4 expression in A) total cohort B) angiosarcoma C) leiomyosarcoma D) undifferentiated pleomorphic sarcoma

A

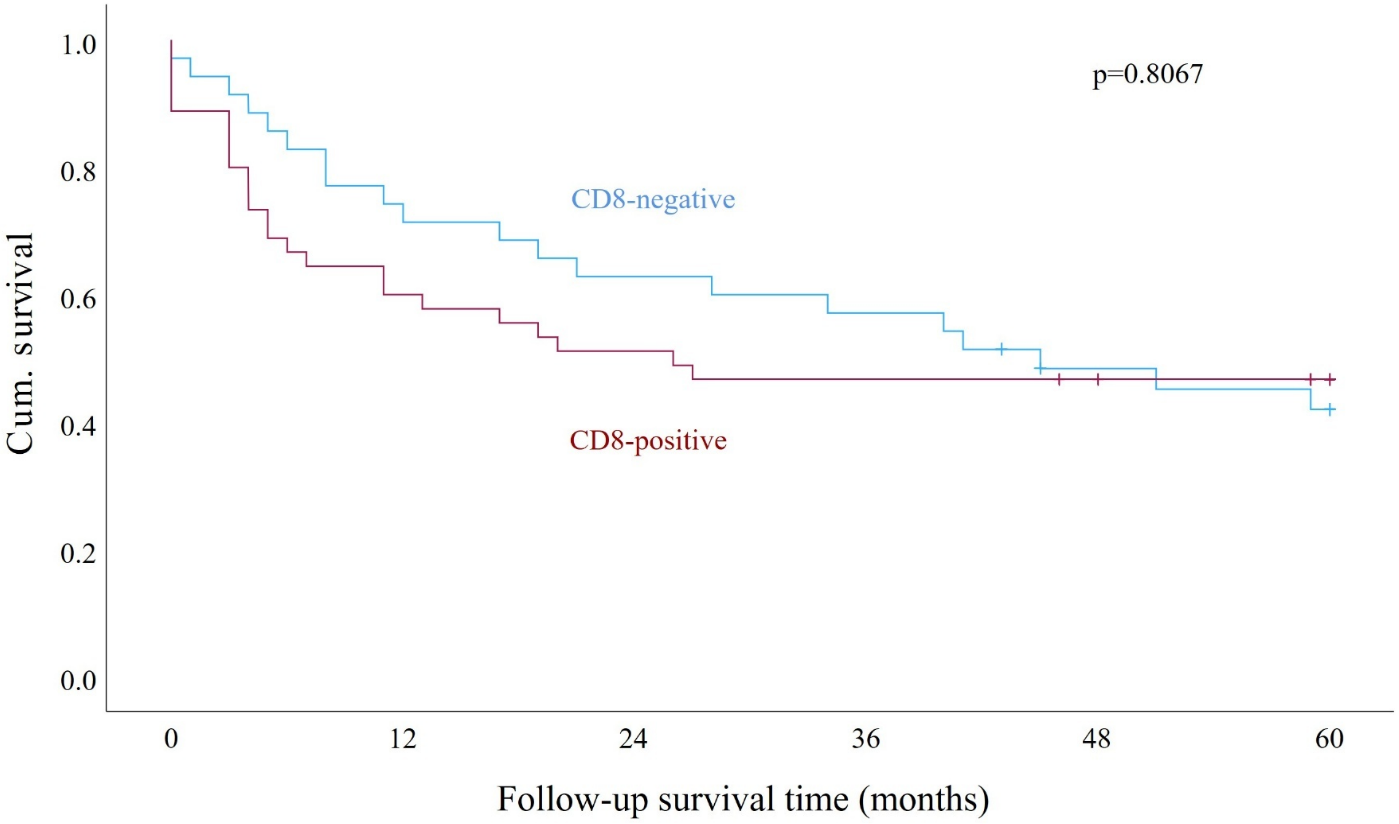
p=0.8067
CD8-negative
CD8-positive
Cum. survival
1.0
0.8
0.6
0.4
0.2
0.0
0
12
24
36
48
60
Follow-up survival time (months)

B

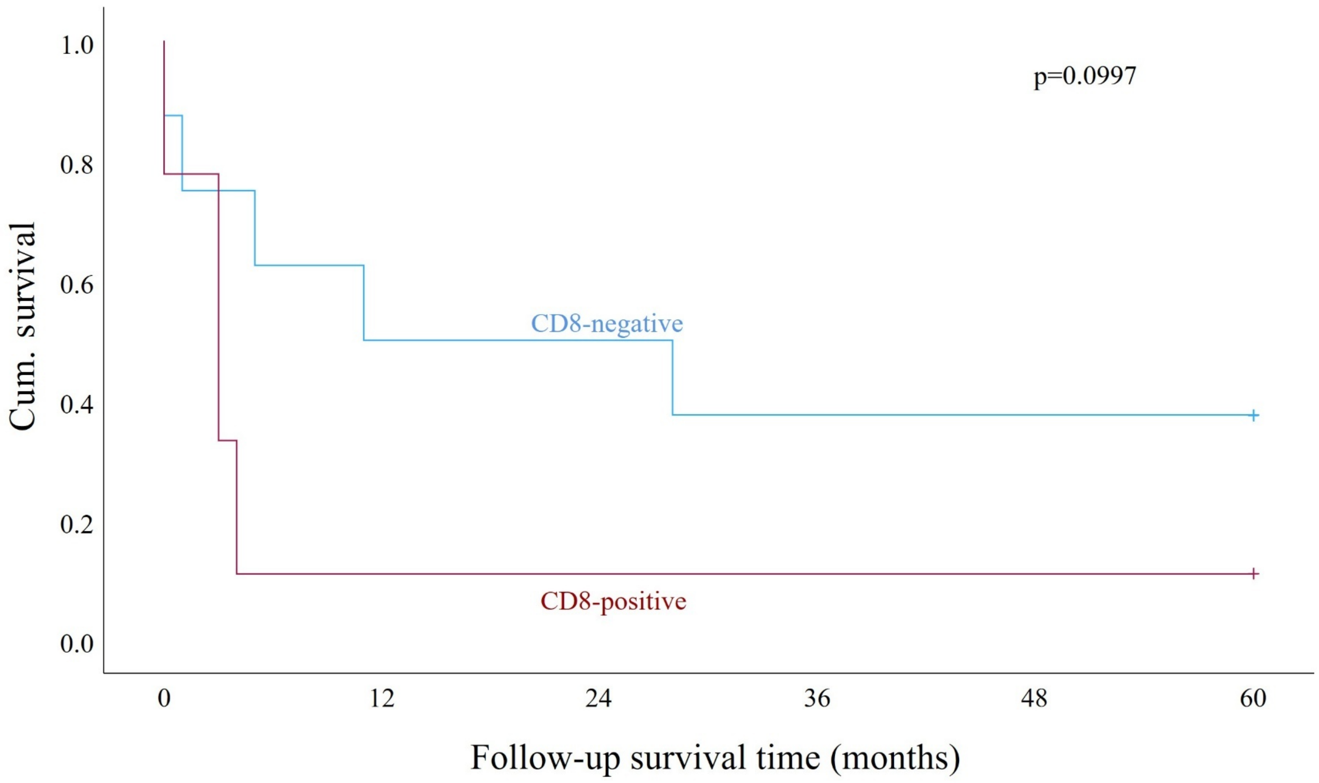

1.0
0.8
0.6
0.4
0.2
0.0
Cum. survival
p=0.0997
CD8-negative
CD8-positive
0
12
24
36
48
60
Follow-up survival time (months)

C

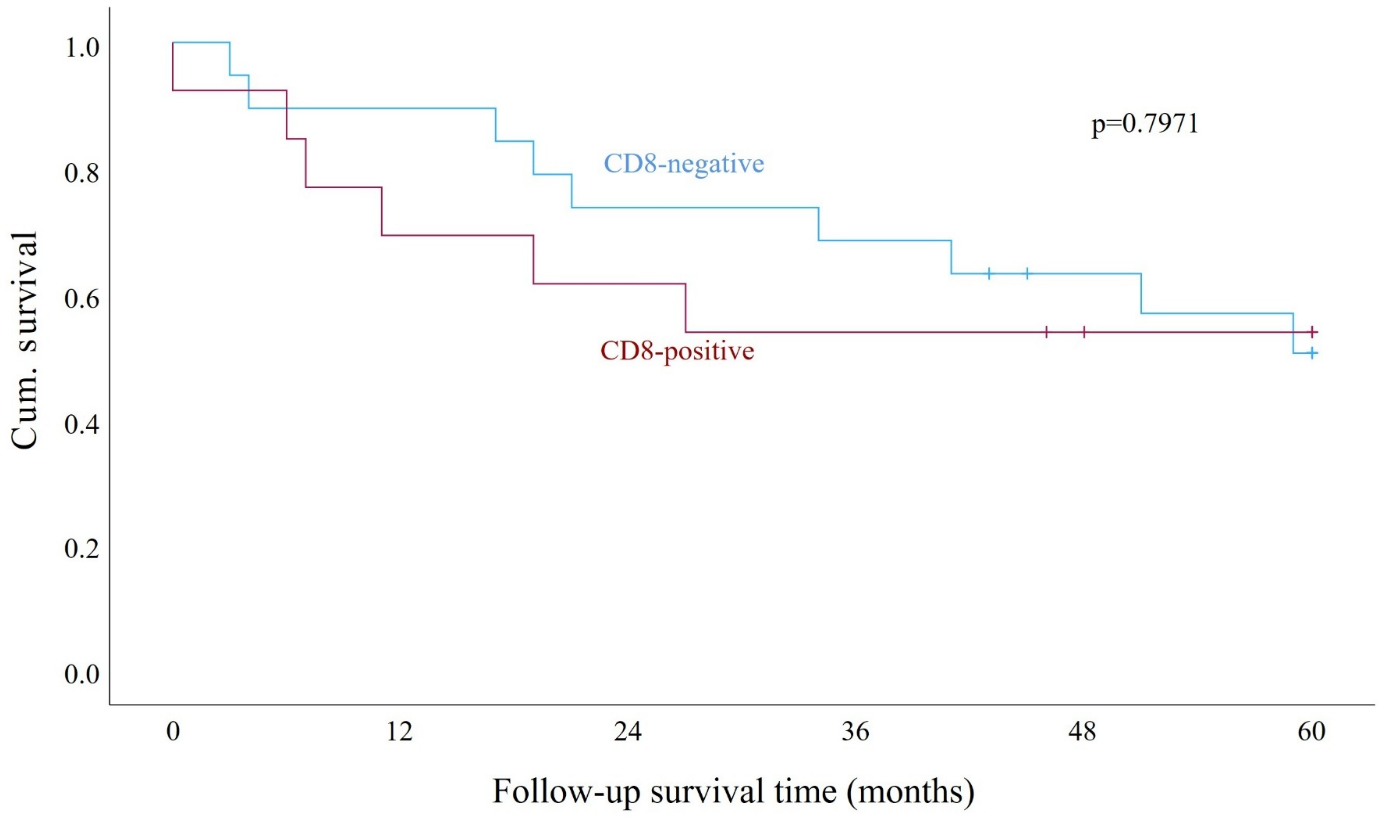

1.0
0.8
0.6
0.4
0.2
0.0
Cum. survival
p=0.7971
CD8-negative
CD8-positive
0
12
24
36
48
60
Follow-up survival time (months)

D

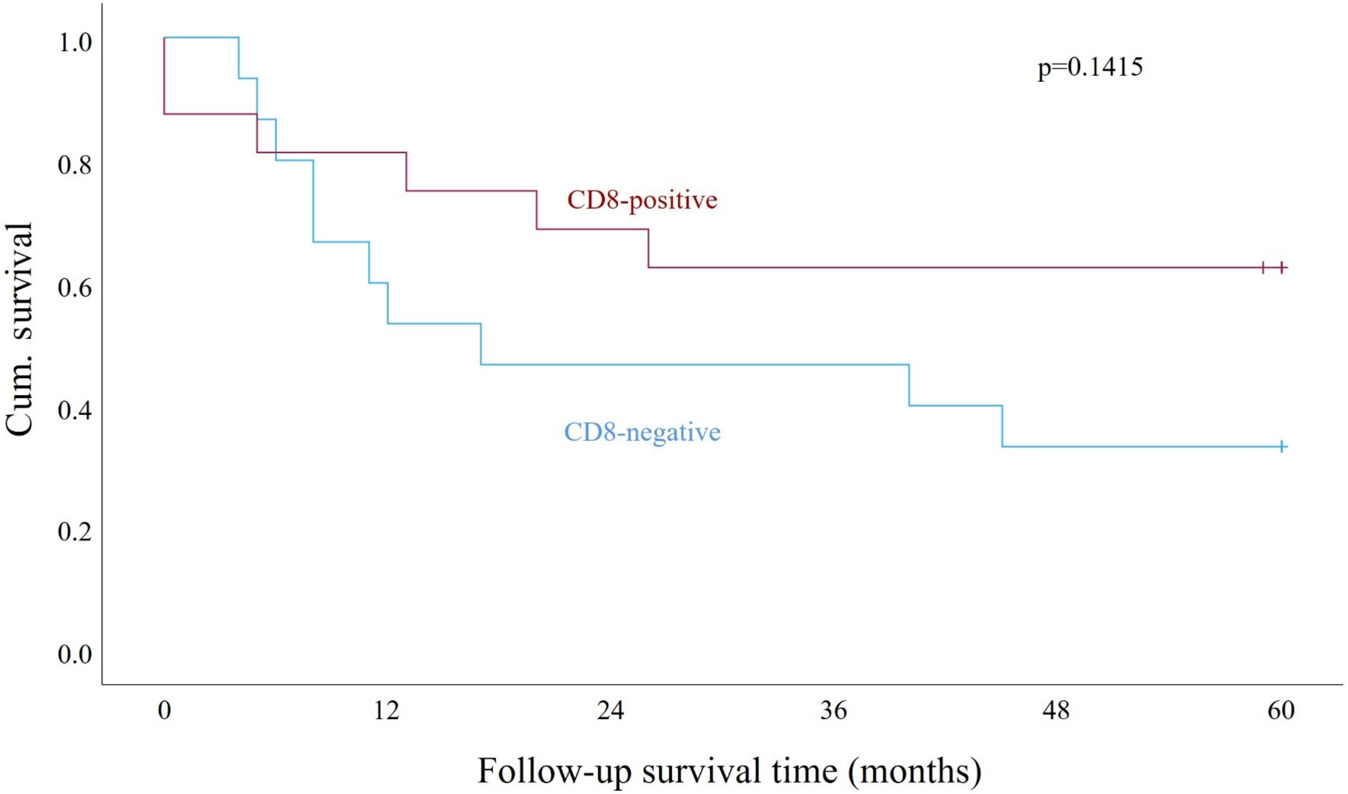

Figure S2A-D: Kaplan-Meier analysis for CD8 expression in A) general cohort B) angiosarcoma C) leiomyosarcoma D) undifferentiated pleomorphic sarcoma

A

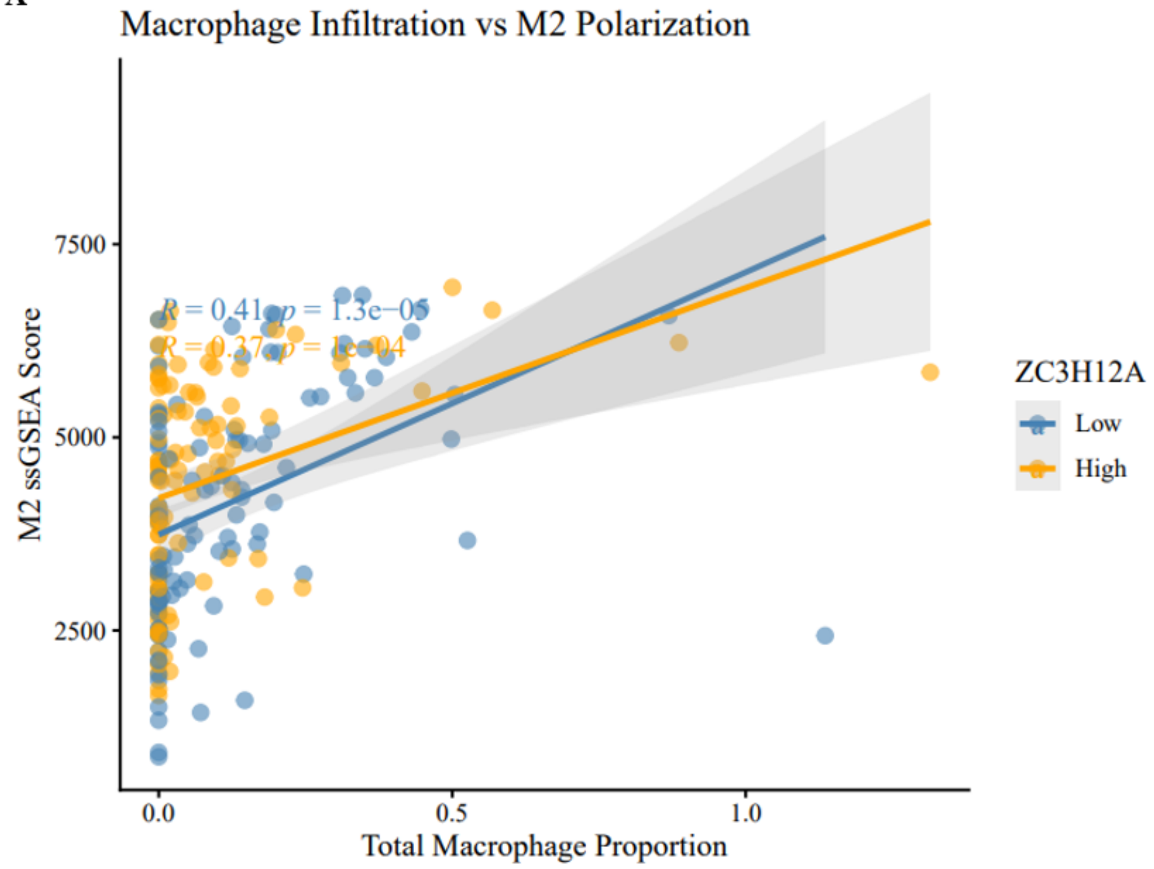

Macrophage Infiltration vs M2 Polarization
M2 ssGSEA Score
7500
5000
2500
0.0
0.5
1.0
Total Macrophage Proportion
R = 0.41, p = 1.3e−05
R = 0.37, p = 1e−04
ZC3H12A
Low
High

B

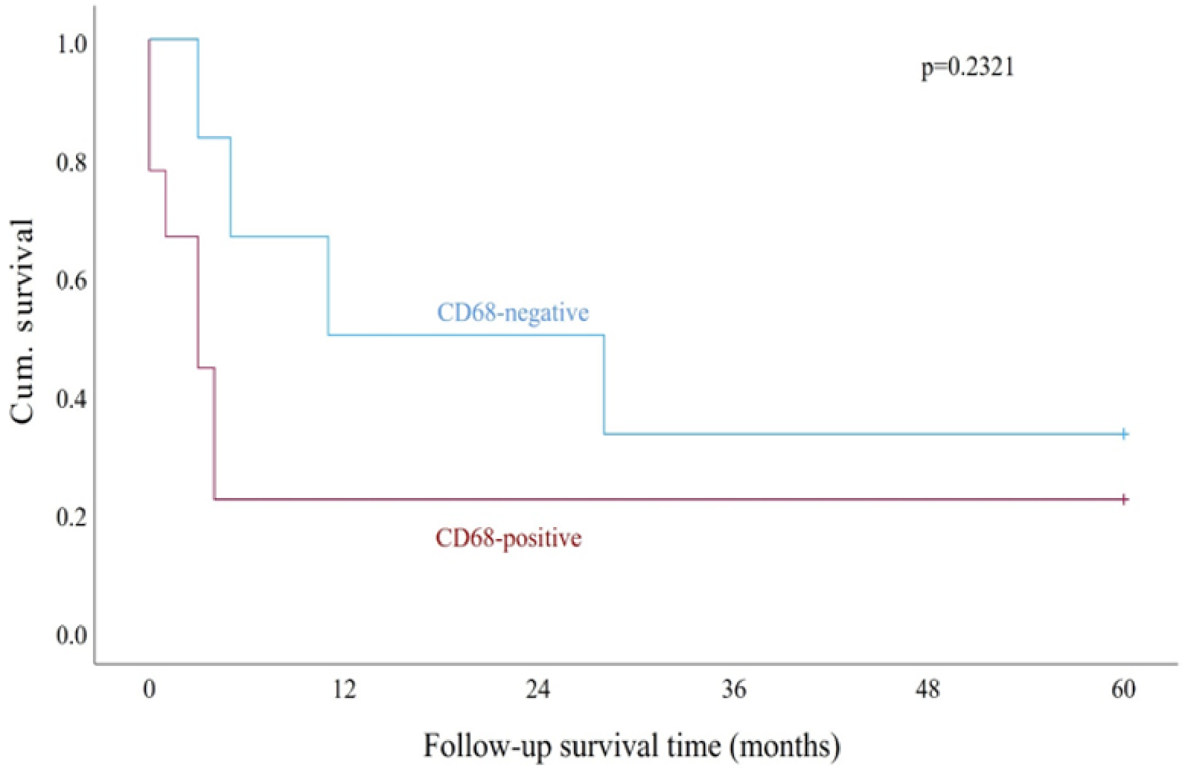

p=0.2321
CD68-negative
CD68-positive
Cum. survival
1.0
0.8
0.6
0.4
0.2
0.0
0
12
24
36
48
60
Follow-up survival time (months)

C

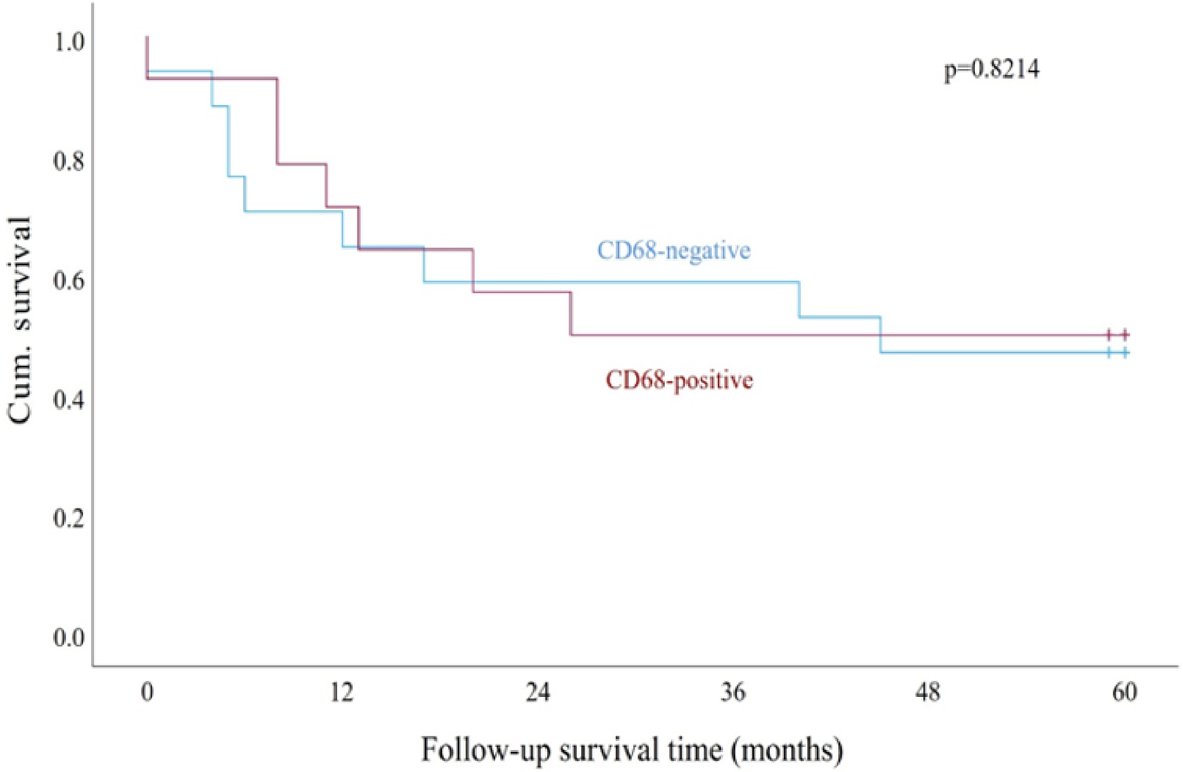

p=0.8214
CD68-negative
CD68-positive
Cum. survival
1.0
0.8
0.6
0.4
0.2
0.0
0
12
24
36
48
60
Follow-up survival time (months)

D

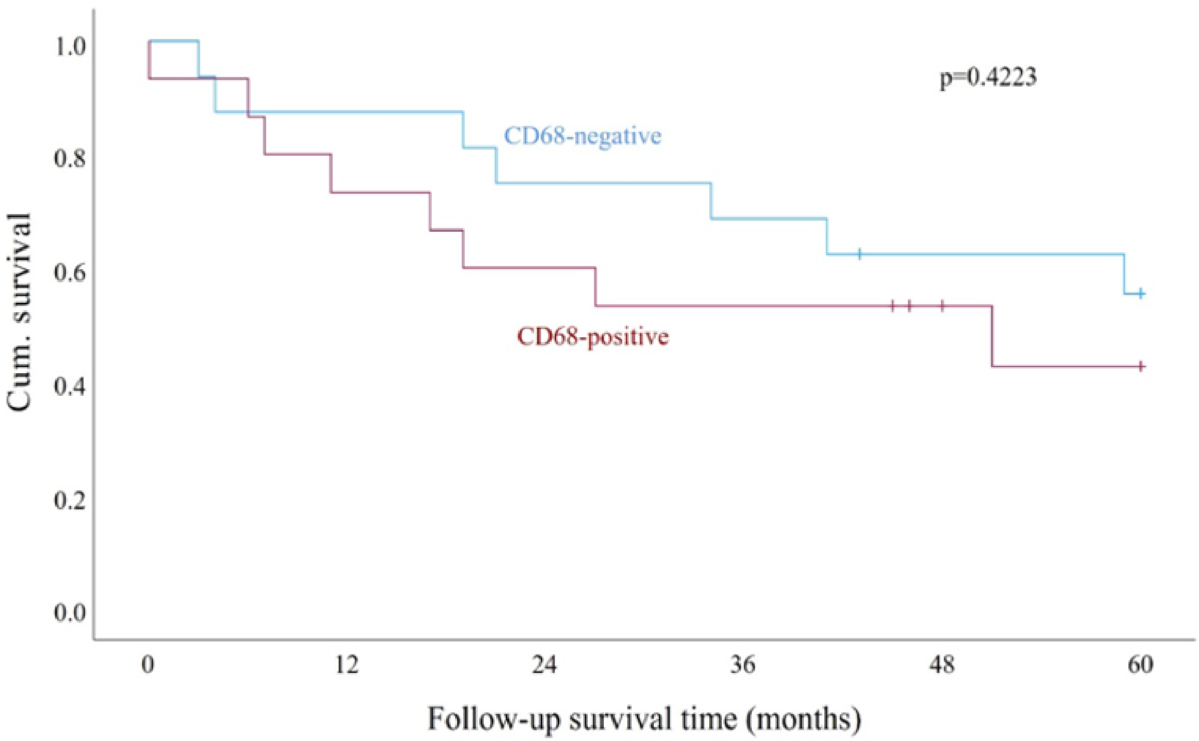

Figure S3A-D. Kaplan-Meier survival analysis stratified by CD68 expression. (A) Significant association between total TAM infiltration and M2-like polarization in both ZC3H12A-low and ZC3H12A-high tumours (ZC3H12A-low: $R = 0.41$, $p = 1.3 \times 10^{-5}$; ZC3H12A-high: $R = 0.37$, $p = 1 \times 10^{-4}$) indicate a relative expansion of M2-like over M1-like-TAMs across both groups. Subgroup analyses of angiosarcoma (B), leiomyosarcoma (C), and undifferentiated pleomorphic sarcoma (D) showed no significant differences in overall survival between CD68-negative and CD68-positive tumors.

A

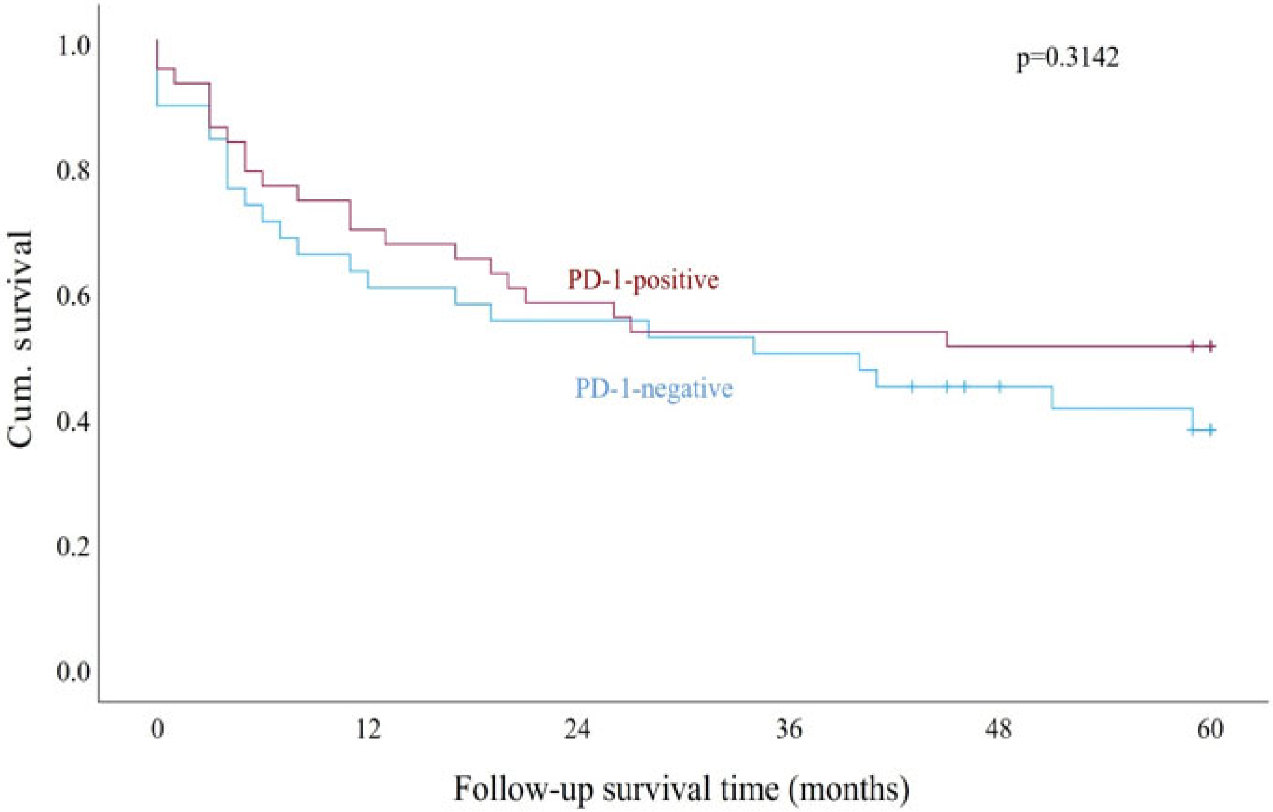
p=0.3142
PD-1-positive
PD-1-negative
Cum. survival
1.0
0.8
0.6
0.4
0.2
0.0
0
12
24
36
48
60
Follow-up survival time (months)

B

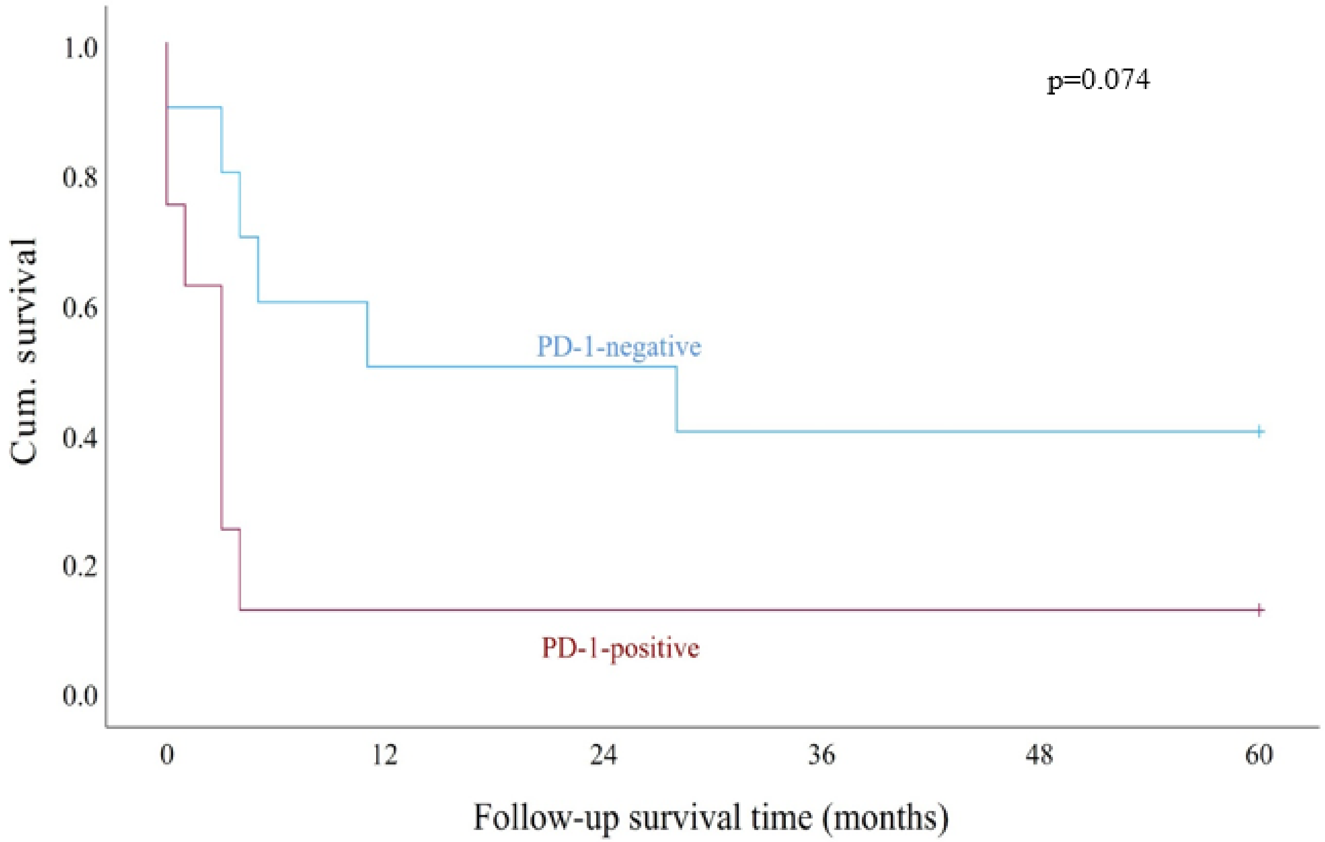

p=0.074
Cum. survival
1.0
0.8
0.6
0.4
0.2
0.0
PD-1-negative
PD-1-positive
0
12
24
36
48
60
Follow-up survival time (months)

C

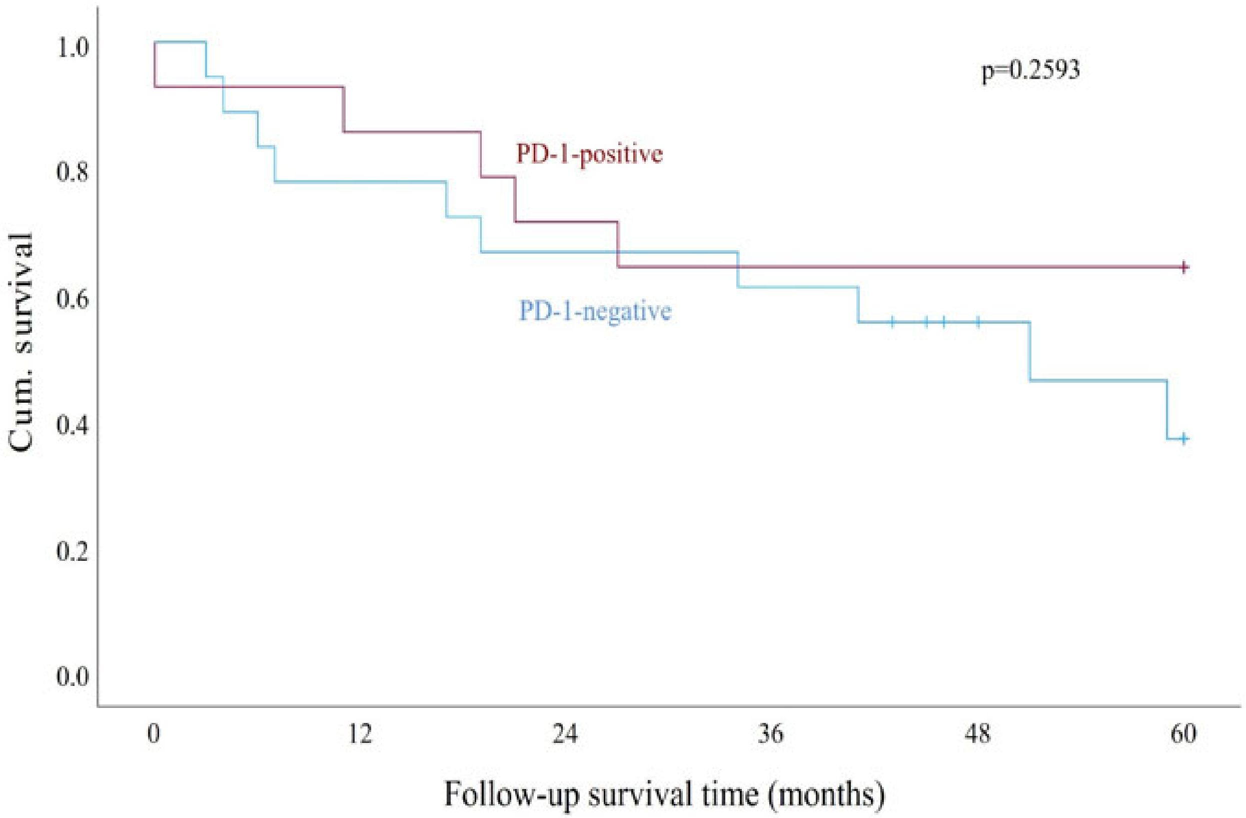

p=0.2593
PD-1-positive
PD-1-negative
Cum. survival
1.0
0.8
0.6
0.4
0.2
0.0
0
12
24
36
48
60
Follow-up survival time (months)

D

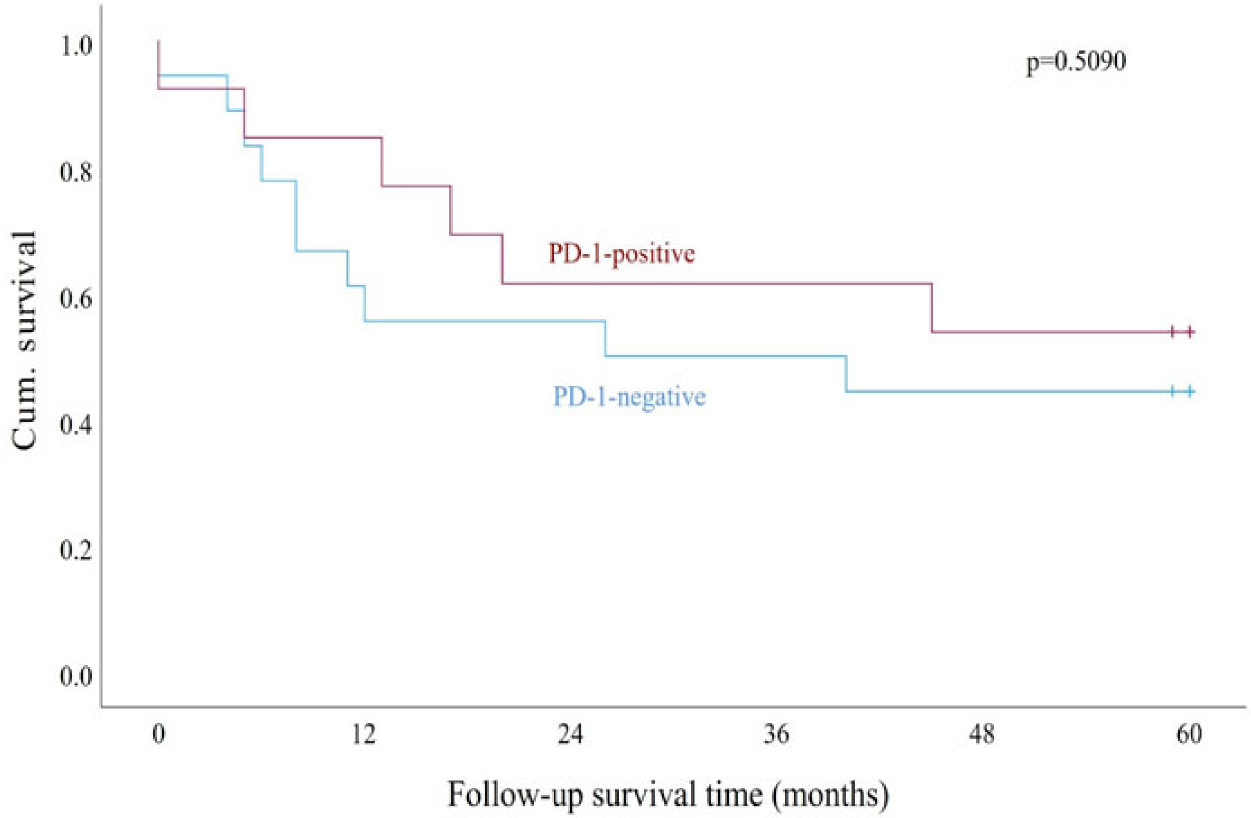

Figure S4A-D. Kaplan-Meier survival analysis stratified by PD-1 expression. In the overall cohort (A), in angiosarcoma (B) in leiomyosarcoma (C), and in undifferentiated pleomorphic sarcoma (D) no significant difference in overall survival was observed between PD-1-positive and PD-1-negative tumors.

A

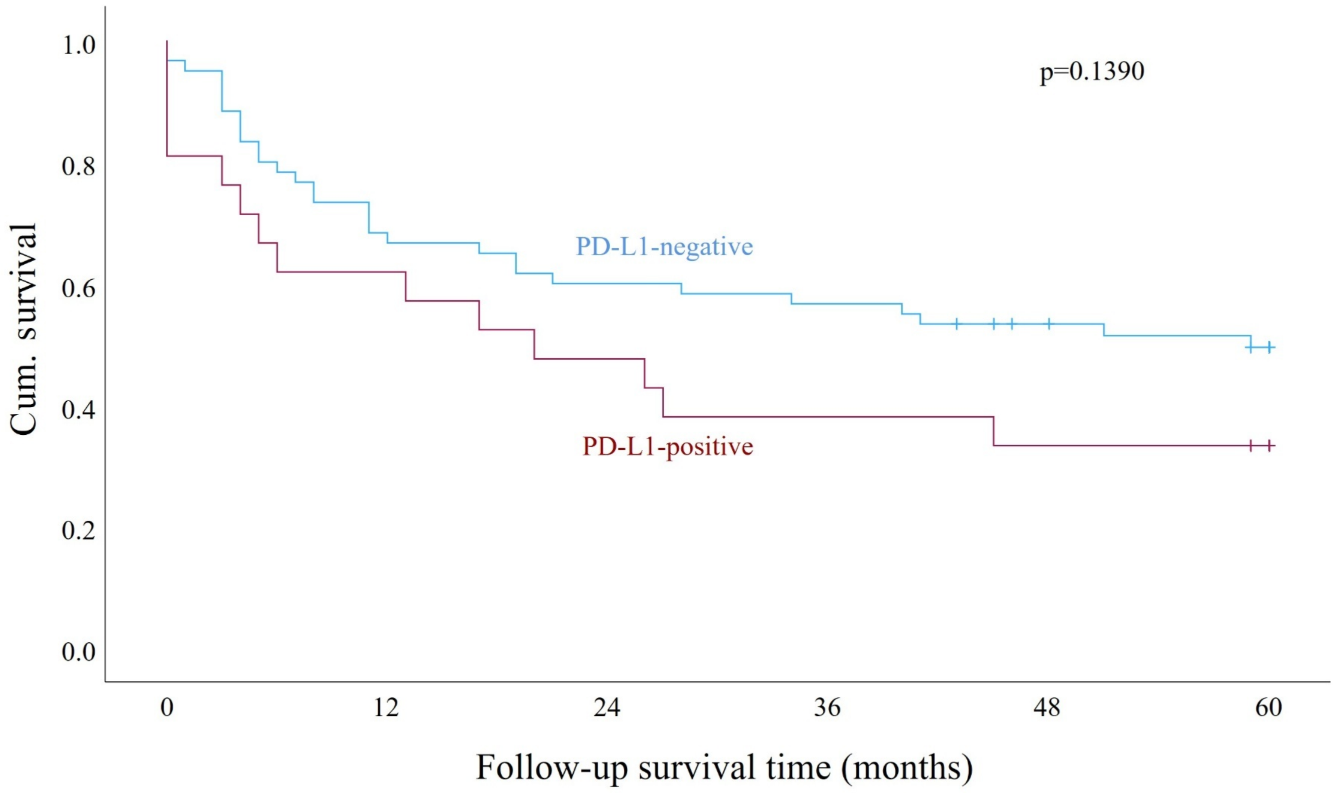
1.0
0.8
0.6
0.4
0.2
0.0
Cum. survival
p=0.1390
PD-L1-negative
PD-L1-positive
0
12
24
36
48
60
Follow-up survival time (months)

B

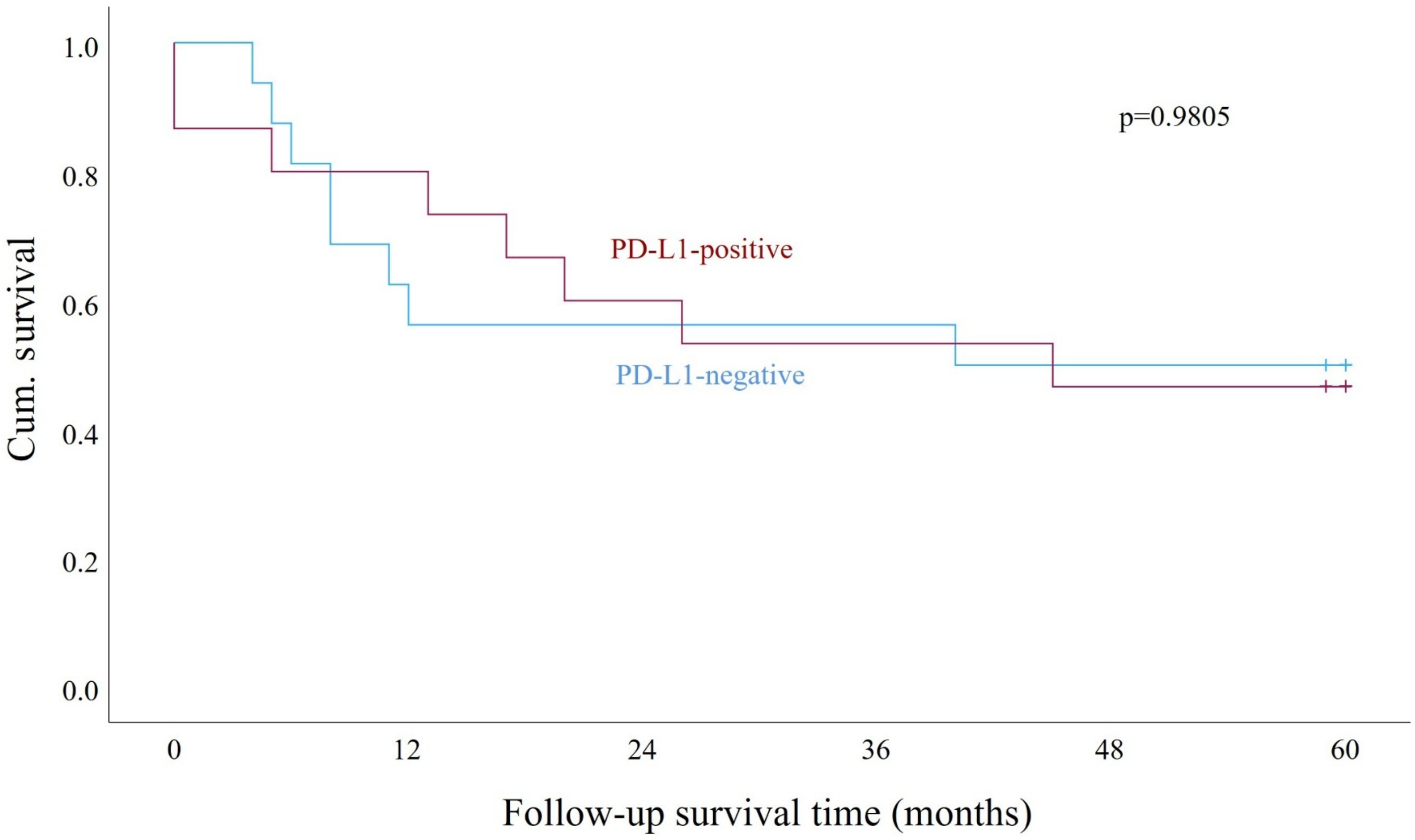

Figure S5A, and B: Kaplan-Meier analysis for PD-L1 expression in A) general cohort B) undifferentiated pleomorphic sarcoma

A

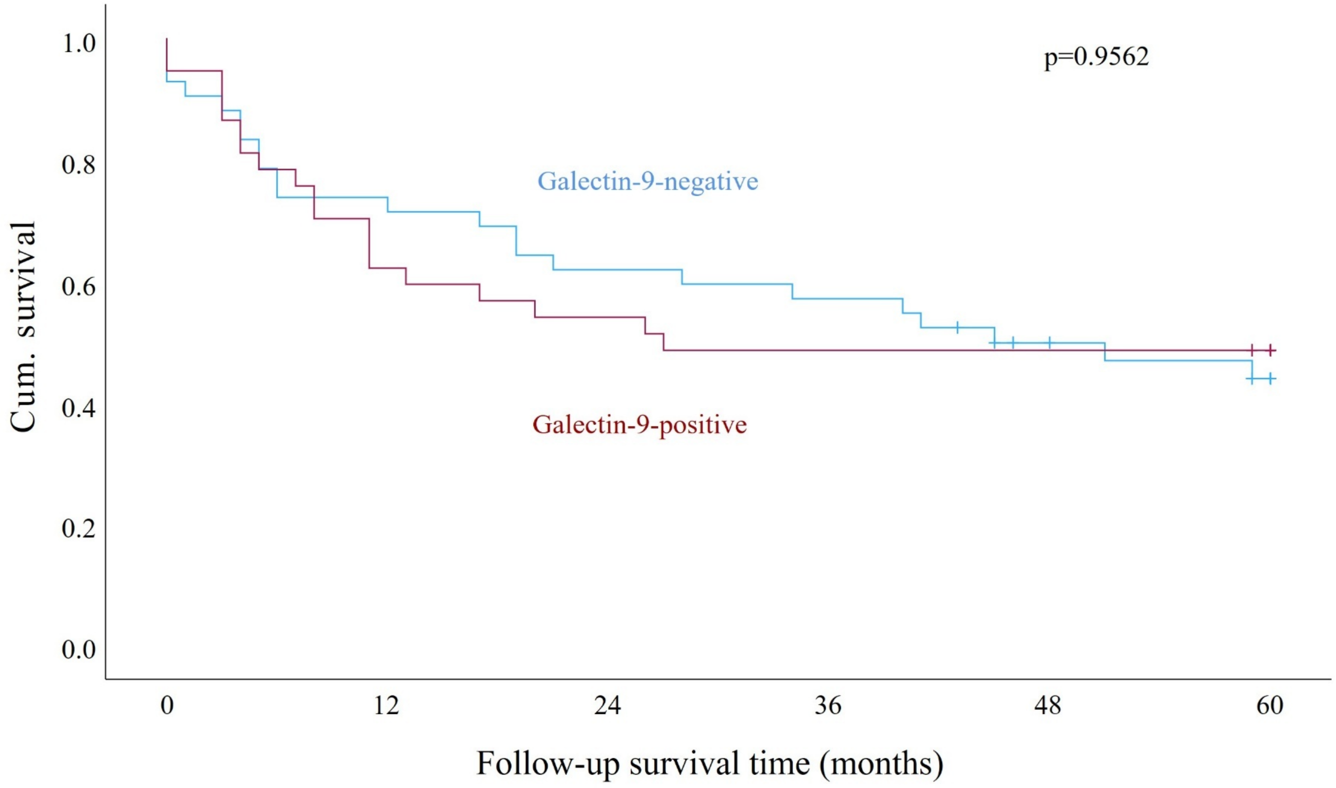

p=0.9562
Galectin-9-negative
Galectin-9-positive
Cum. survival
1.0
0.8
0.6
0.4
0.2
0.0
0
12
24
36
48
60
Follow-up survival time (months)

B

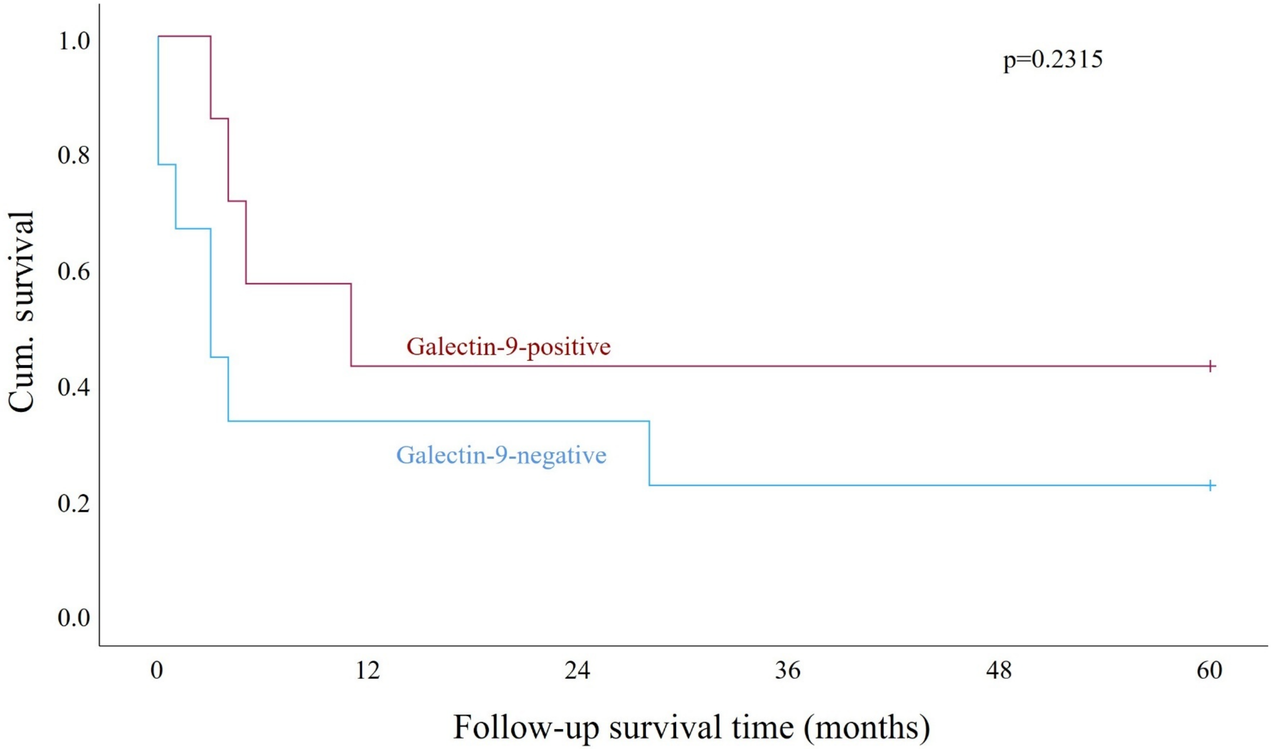
p=0.2315
Cum. survival
1.0
0.8
0.6
0.4
0.2
0.0
Galectin-9-positive
Galectin-9-negative
0
12
24
36
48
60
Follow-up survival time (months)

C

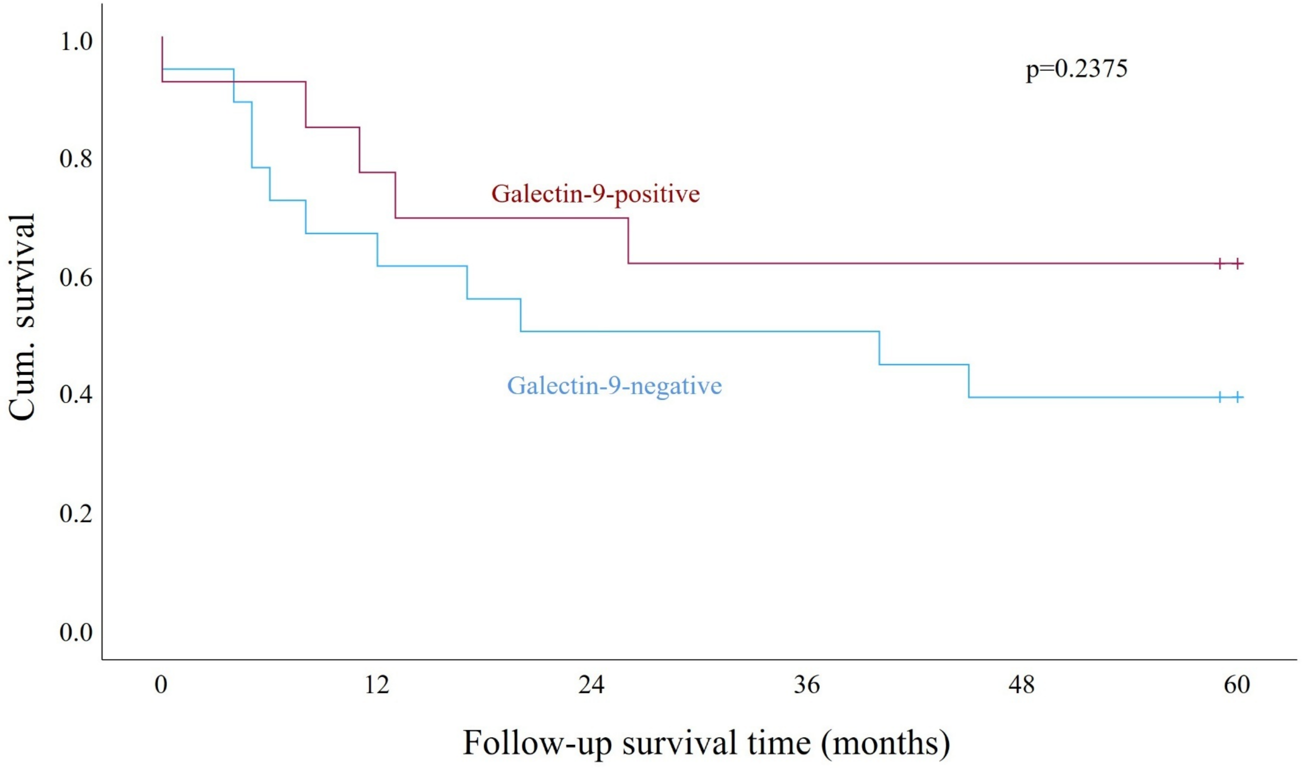

Figure S6A-C: Kaplan-Meier analysis for Galectin-9 expression in A) general cohort B) angiosarcoma C) undifferentiated pleomorphic sarcoma

A

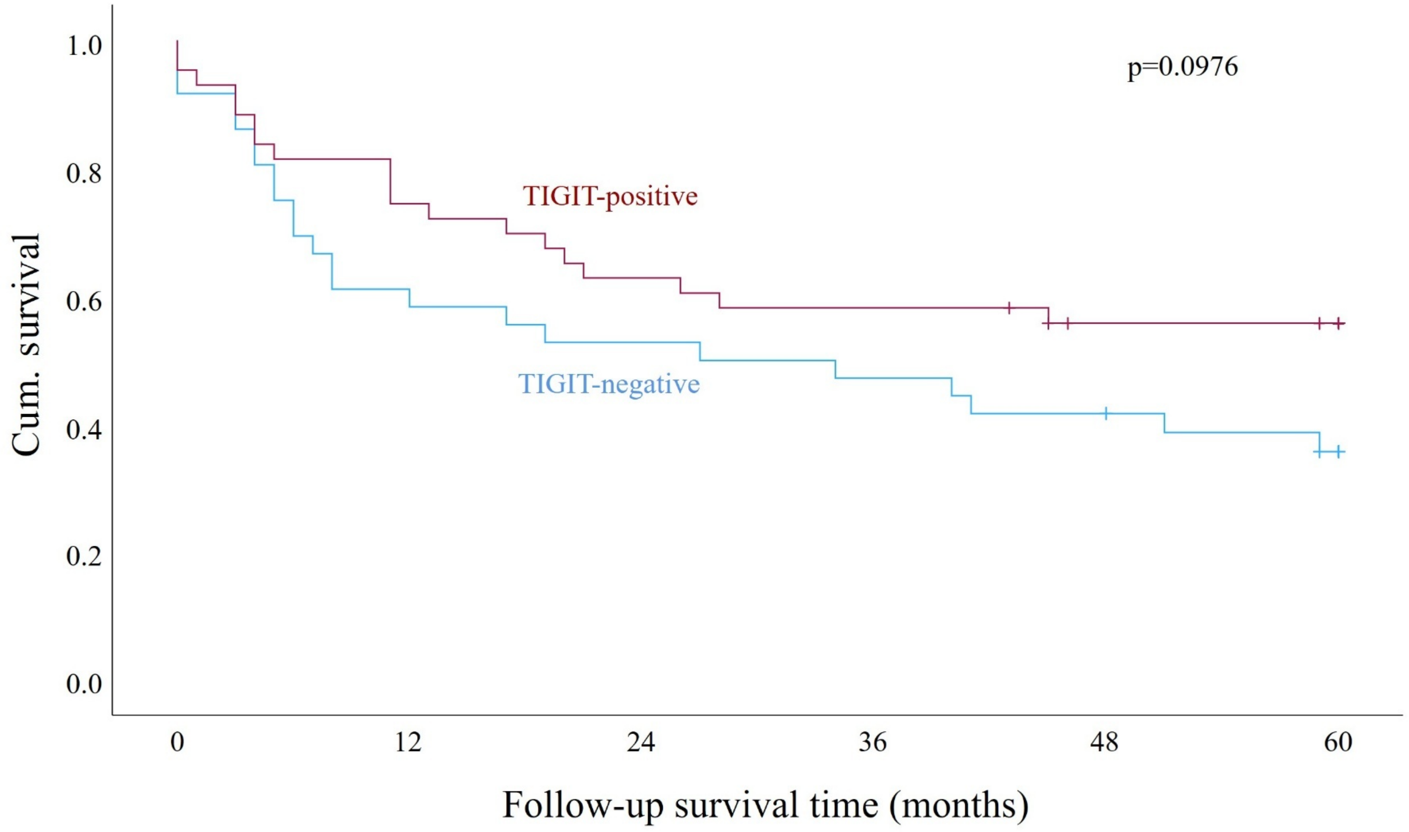
p=0.0976
TIGIT-positive
TIGIT-negative
Cum. survival
1.0
0.8
0.6
0.4
0.2
0.0
0
12
24
36
48
60
Follow-up survival time (months)

B

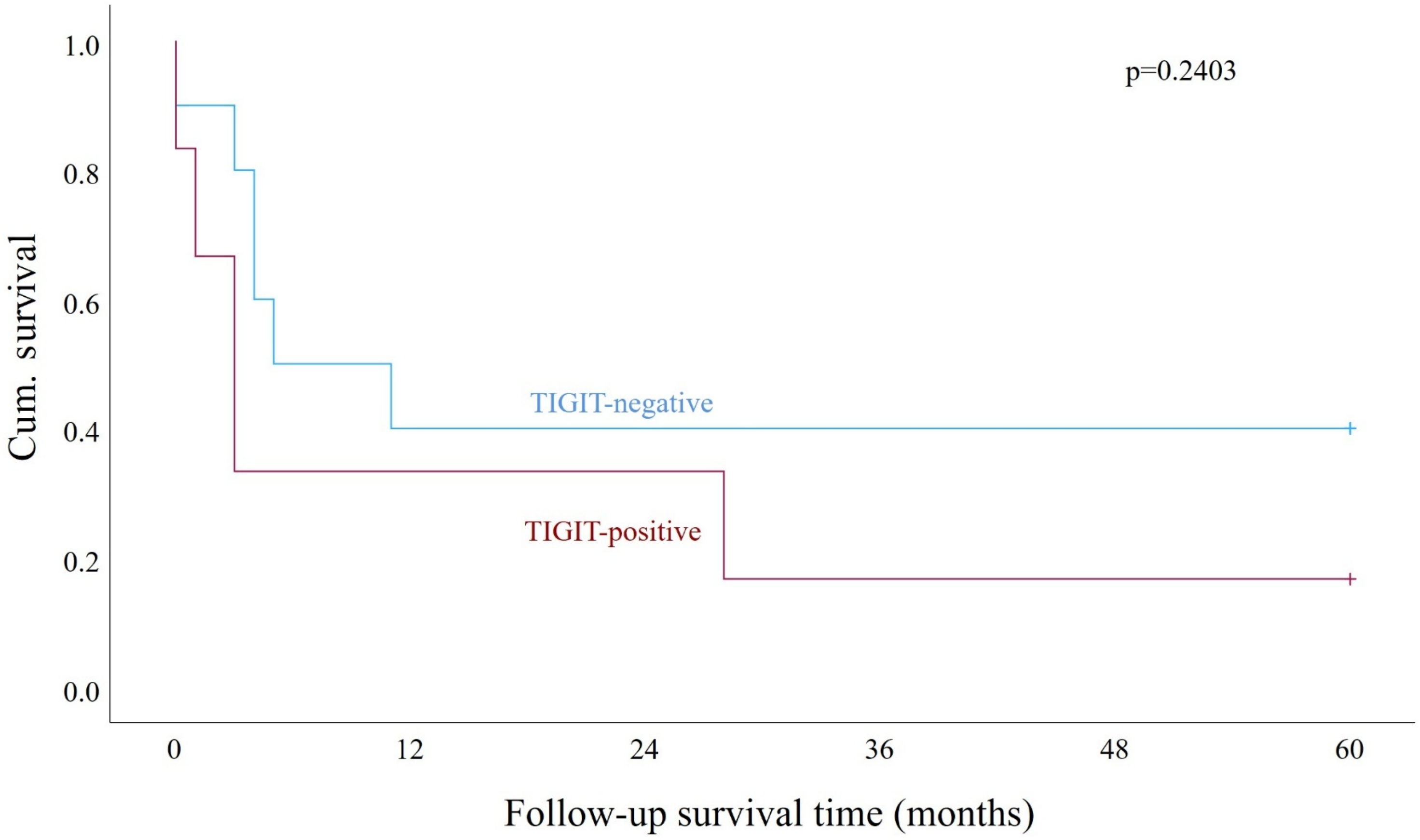
1.0
0.8
0.6
0.4
0.2
0.0
Cum. survival
p=0.2403
TIGIT-negative
TIGIT-positive
0
12
24
36
48
60
Follow-up survival time (months)

C

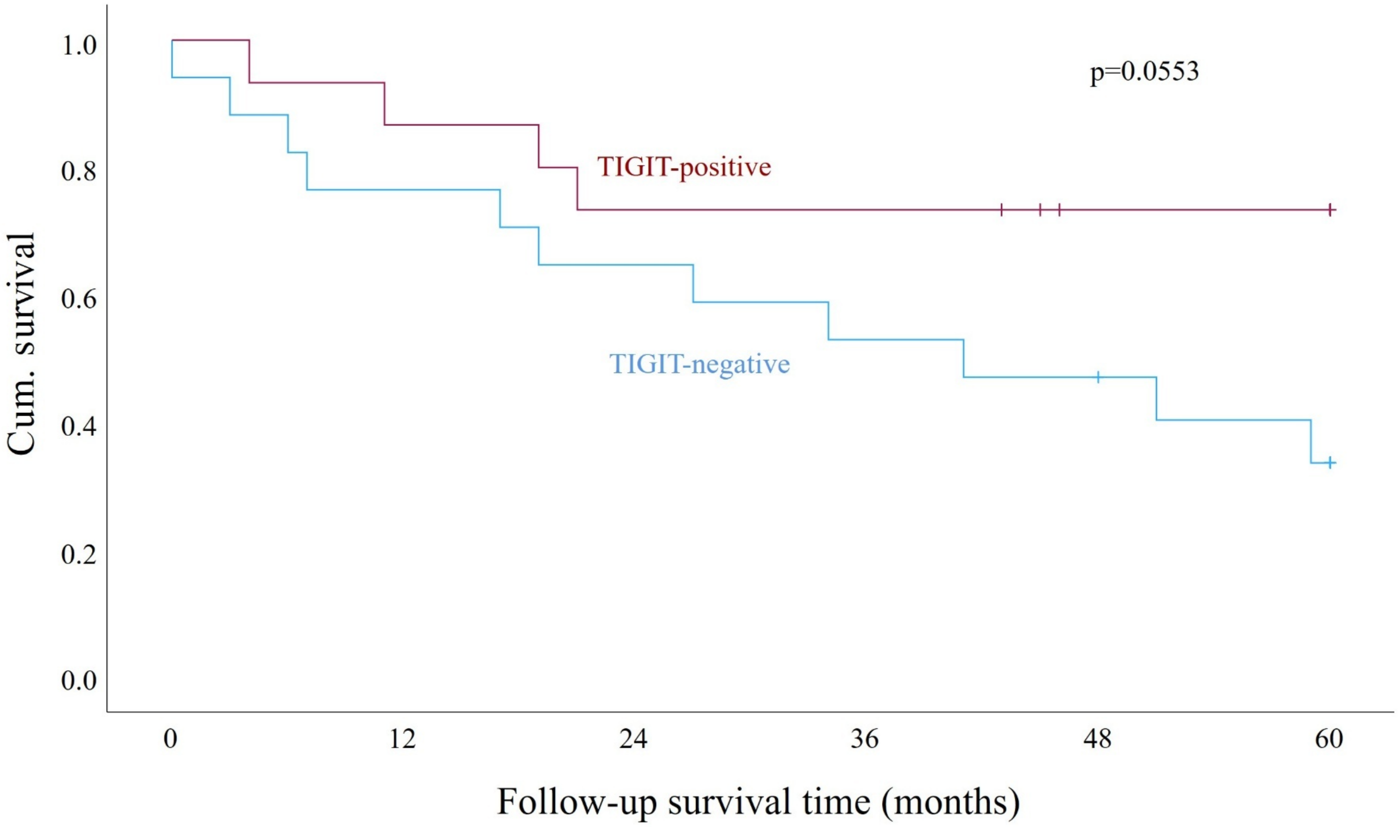

1.0
0.8
0.6
0.4
0.2
0.0
Cum. survival
p=0.0553
TIGIT-positive
TIGIT-negative
0
12
24
36
48
60
Follow-up survival time (months)

D

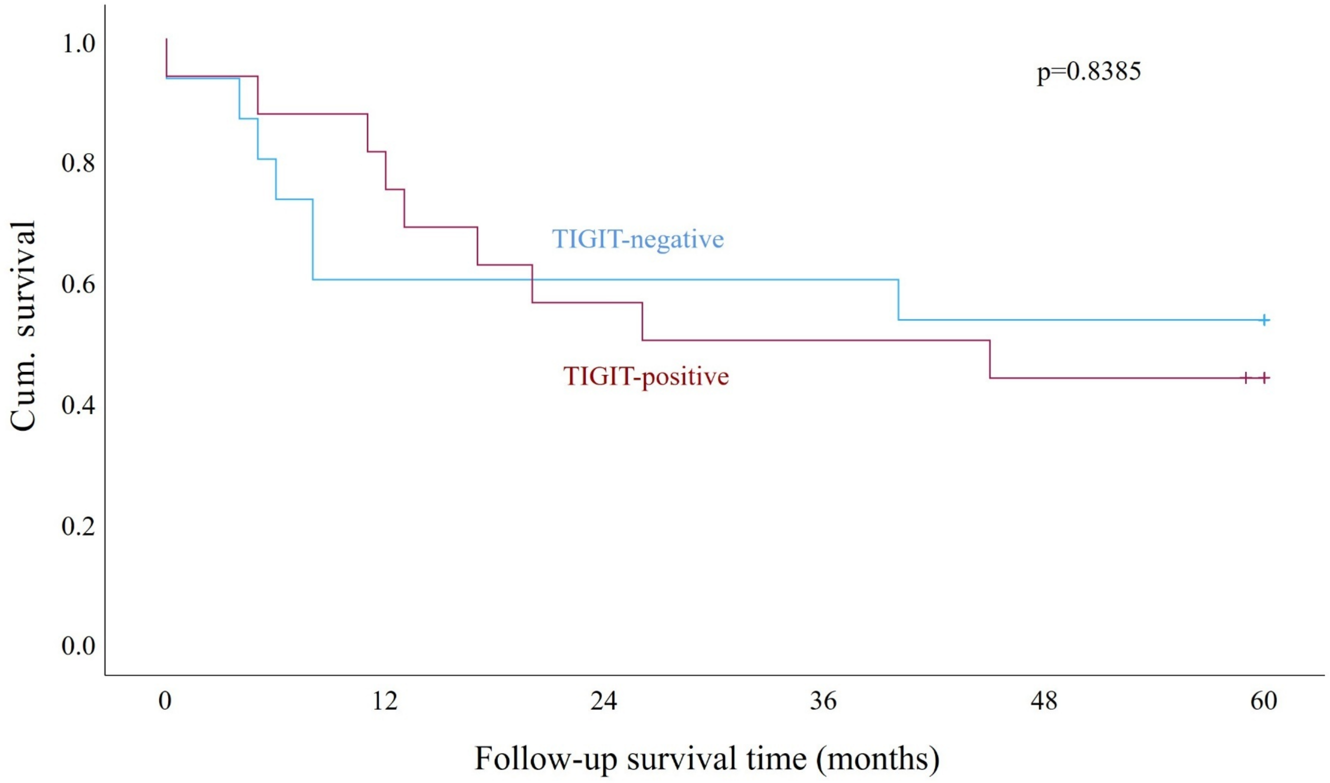

Figure S7A-D: Kaplan-Meer analysis for TIGIT expression in A) general cohort B) angiosarcoma C) leiomyosarcoma D) undifferentiated pleomorphic sarcoma

A

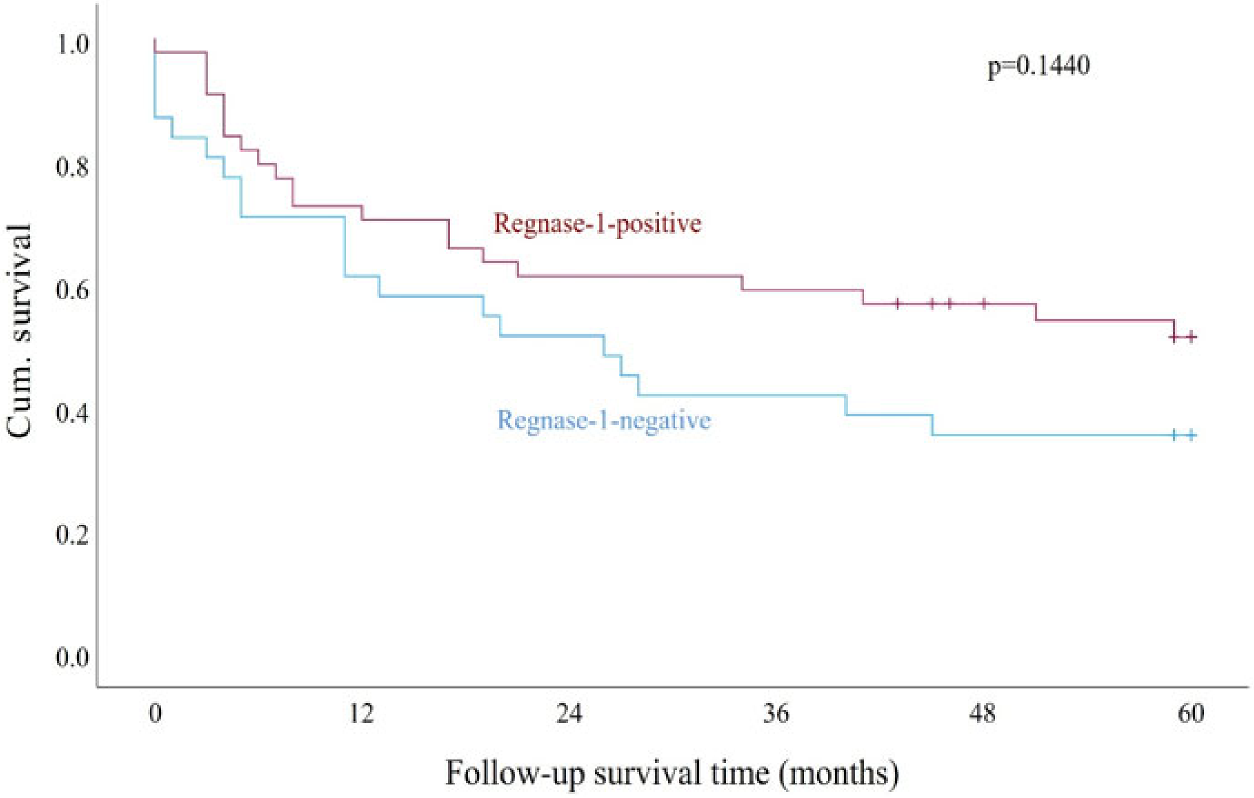

p=0.1440
Regnase-1-positive
Regnase-1-negative
Cum. survival
1.0
0.8
0.6
0.4
0.2
0.0
0
12
24
36
48
60
Follow-up survival time (months)

B

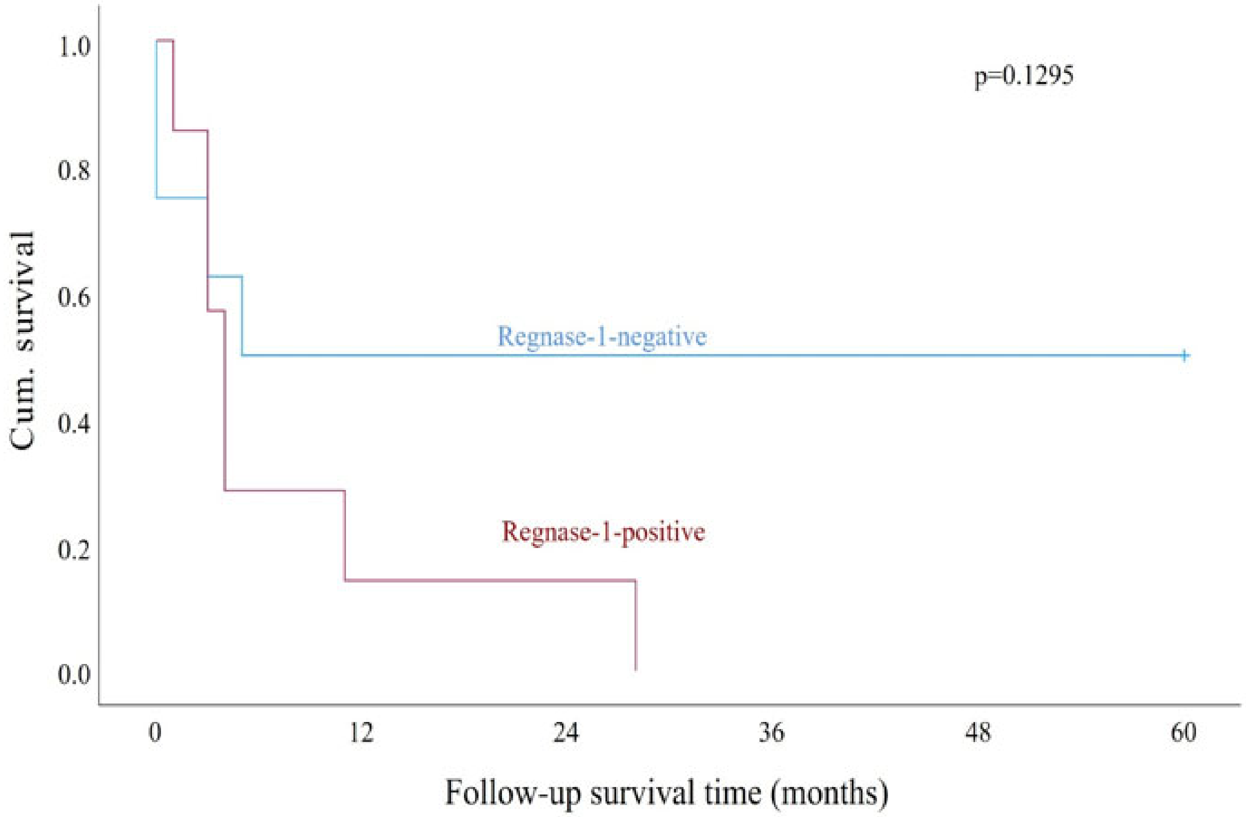
1.0
0.8
0.6
0.4
0.2
0.0
Cum. survival
p=0.1295
Regnase-1-negative
Regnase-1-positive
0
12
24
36
48
60
Follow-up survival time (months)

C

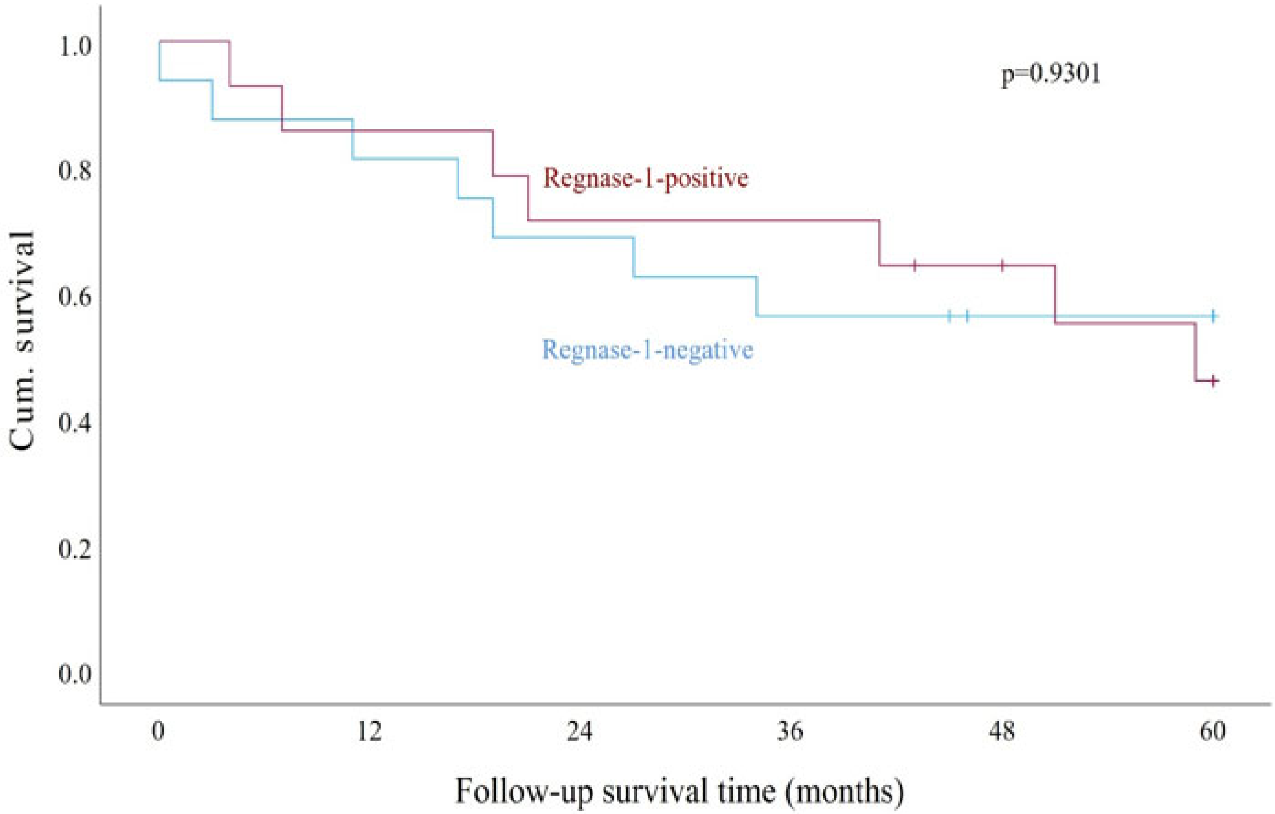

Figure S8A-C. Kaplan–Meier survival analysis stratified by Regnase-1 expression. In the overall cohort (A), a numerical trend toward better overall survival was observed between Regnase-1-positive and Regnase-1-negative tumors (*P* = 0.1440). Subgroup analyses of angiosarcoma (B), and leiomyosarcoma (C) did not show a significant survival impact for Regnase-1-positive vs. Regnase-1-negative.

A

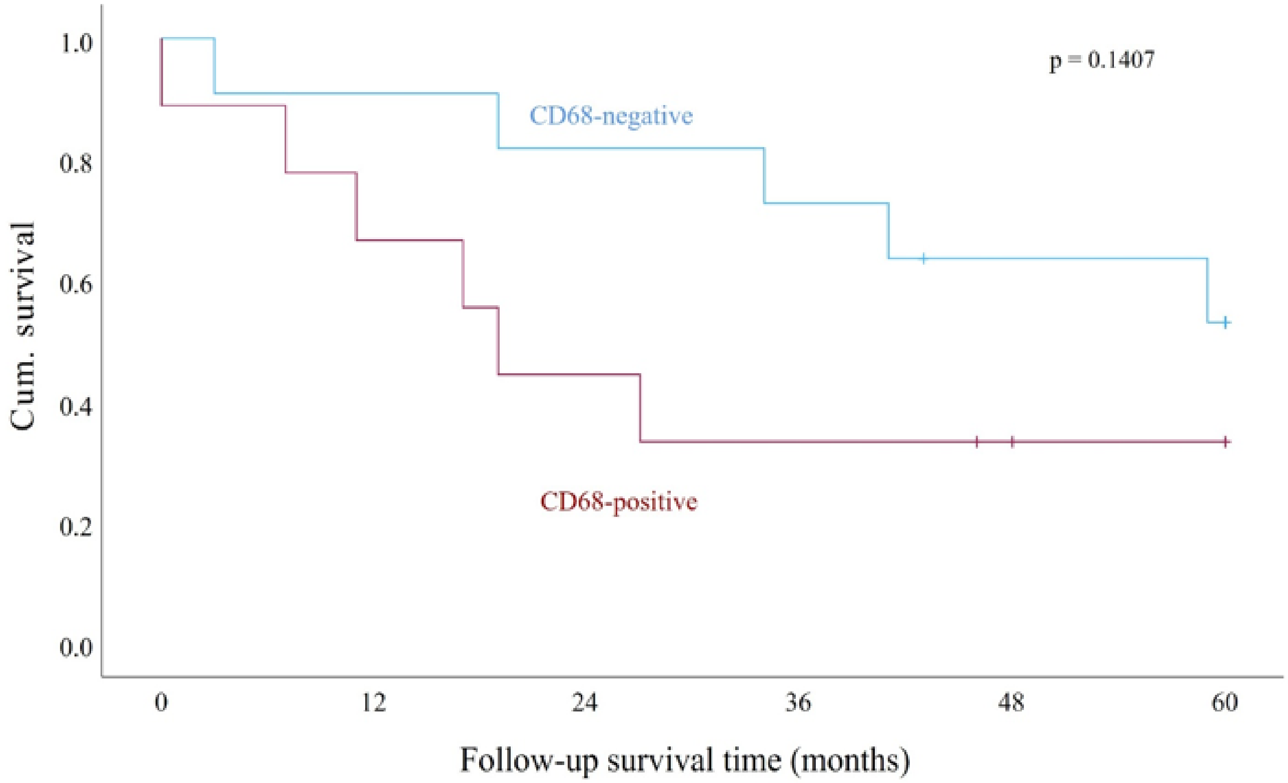

p = 0.1407
CD68-negative
CD68-positive
Cum. survival
1.0
0.8
0.6
0.4
0.2
0.0
0
12
24
36
48
60
Follow-up survival time (months)

B

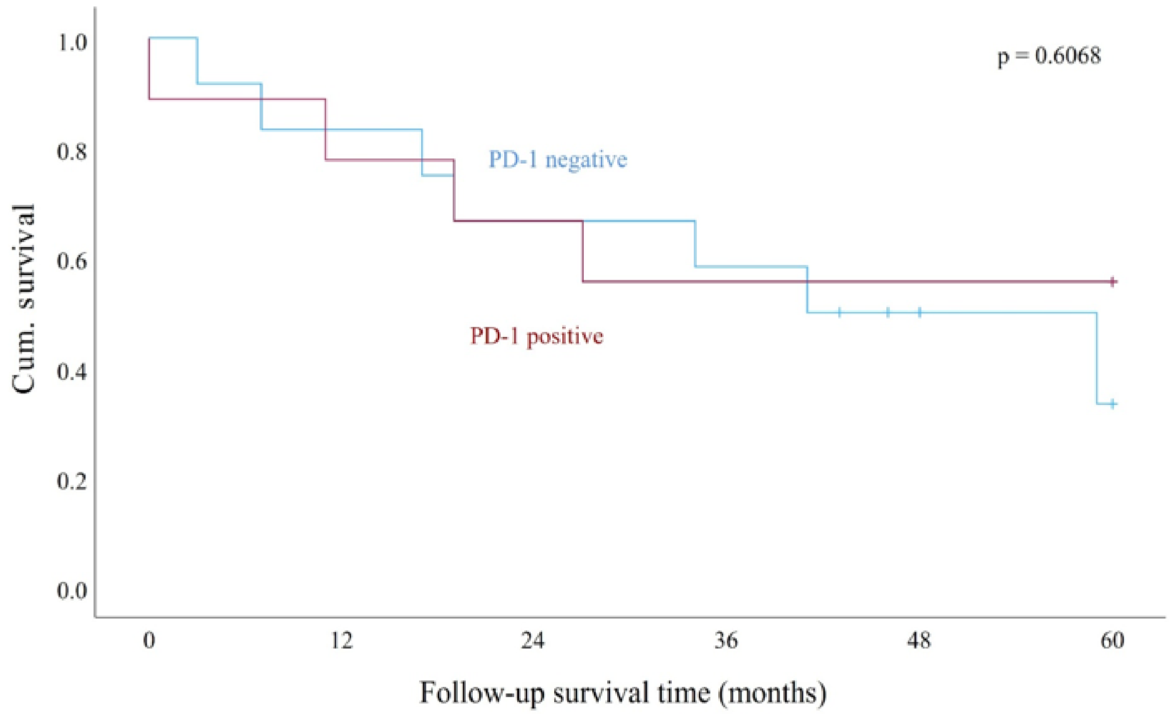

Figure S9A-B. **Kaplan–Meier survival analysis for non-uterine leiomyosarcoma in the overall cohort. Stratification by CD68 and PD-1.** No siginifcant changes were observerd by CD68 (A) or PD-1 expression status (B).

A

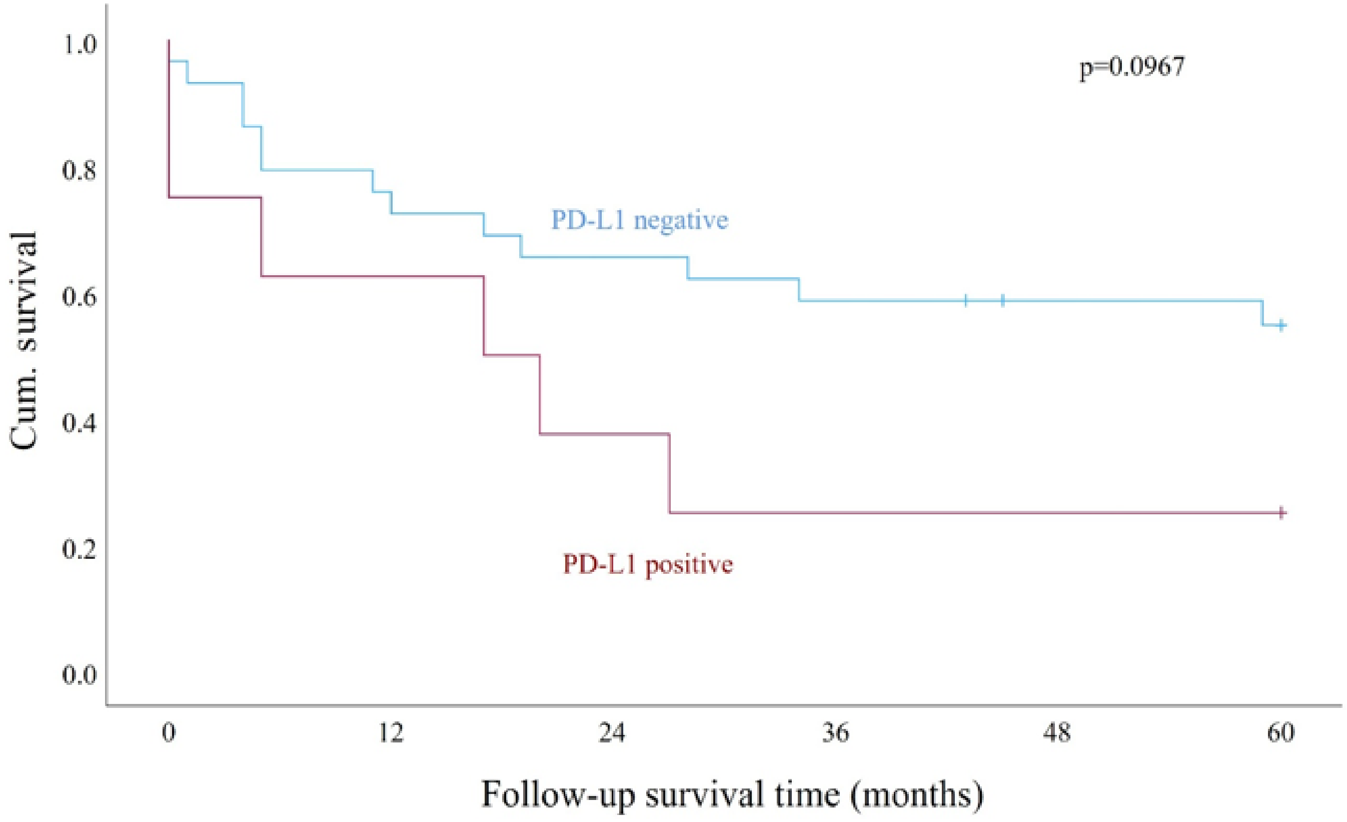

p=0.0967
PD-L1 negative
PD-L1 positive
Cum. survival
1.0
0.8
0.6
0.4
0.2
0.0
0
12
24
36
48
60
Follow-up survival time (months)

B

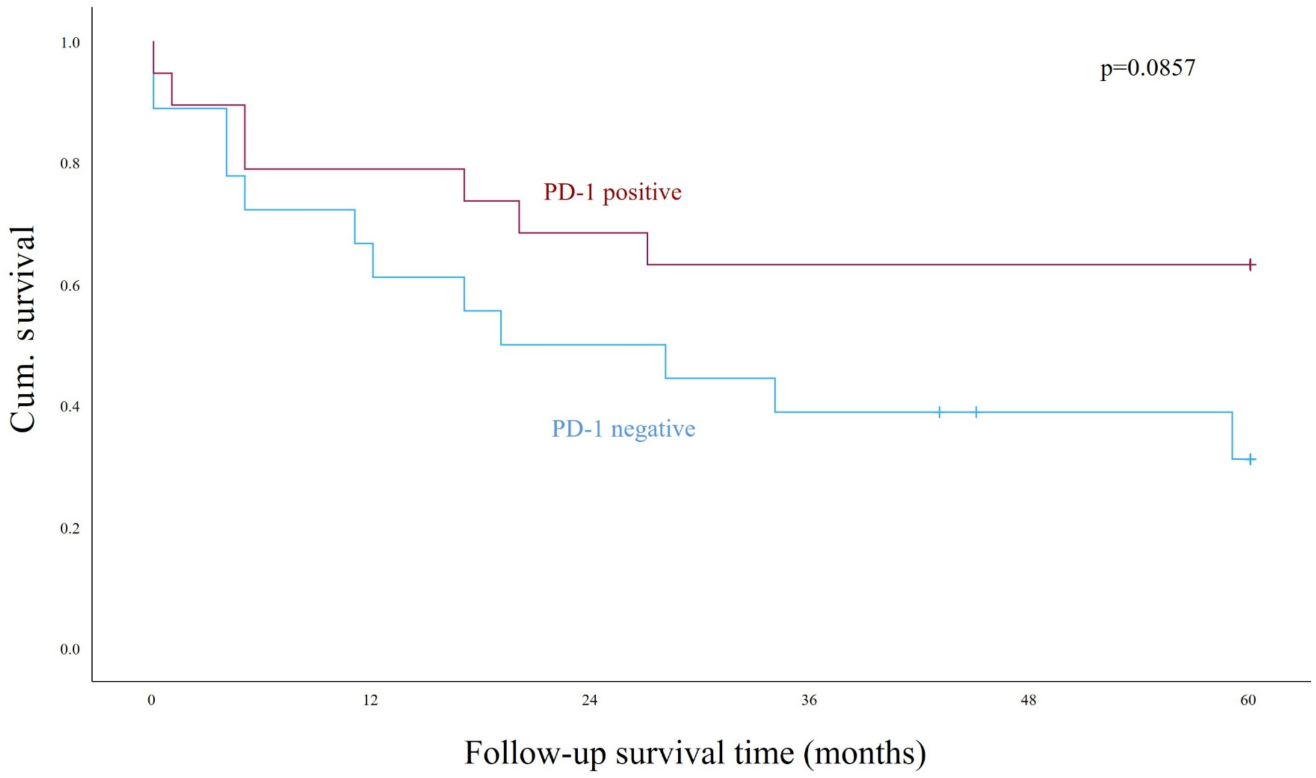

Figure S10A, and B: Kaplan-Meier analysis for PD-1 expression in patients treated with surgery only. (A) PD-L1/CPS positivity was numerically but not significantly associated with reduced mOS in the cohort of surgery-only treated patients: mOS: 17.0 vs. NR; P = 0.0967). (B) No significant effect by PD-1 expression status.

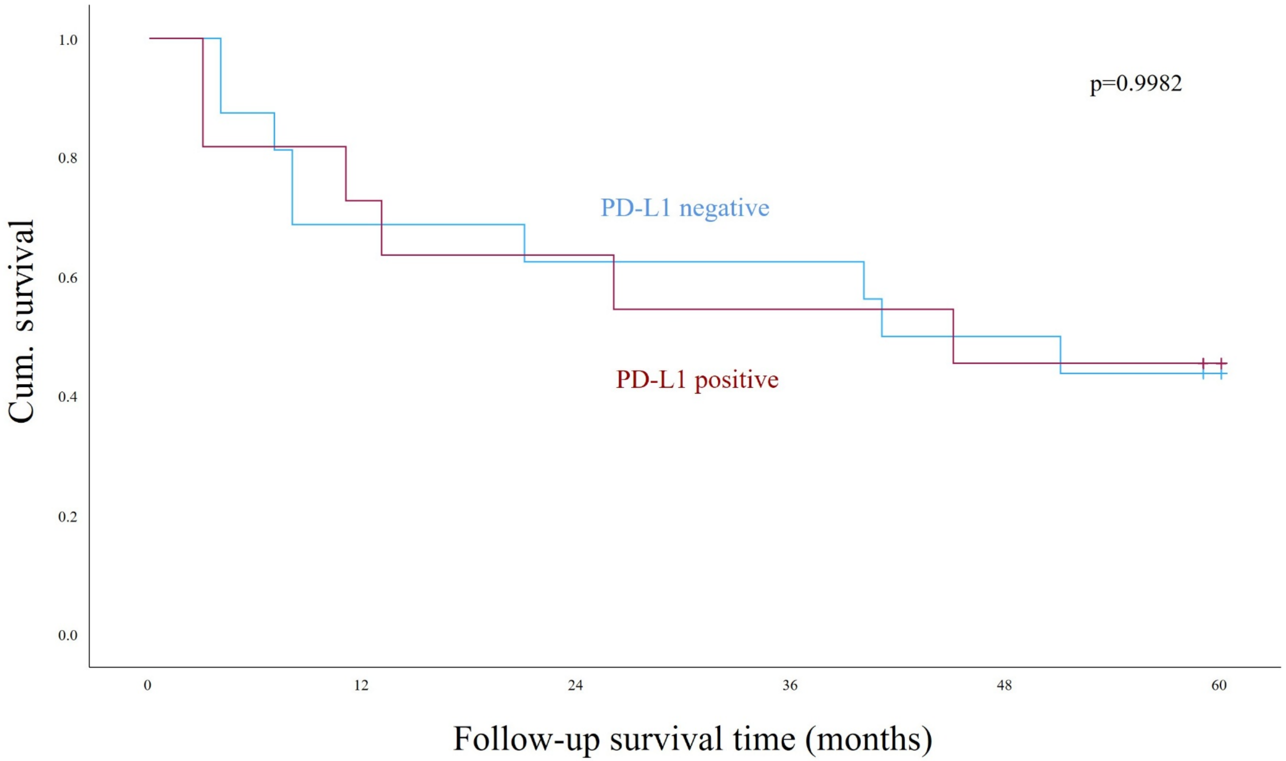

Figure S11: Kaplan-Meier survival analysis for PD-L1 expression in subgroup "patients with surgery and radiation therapy"

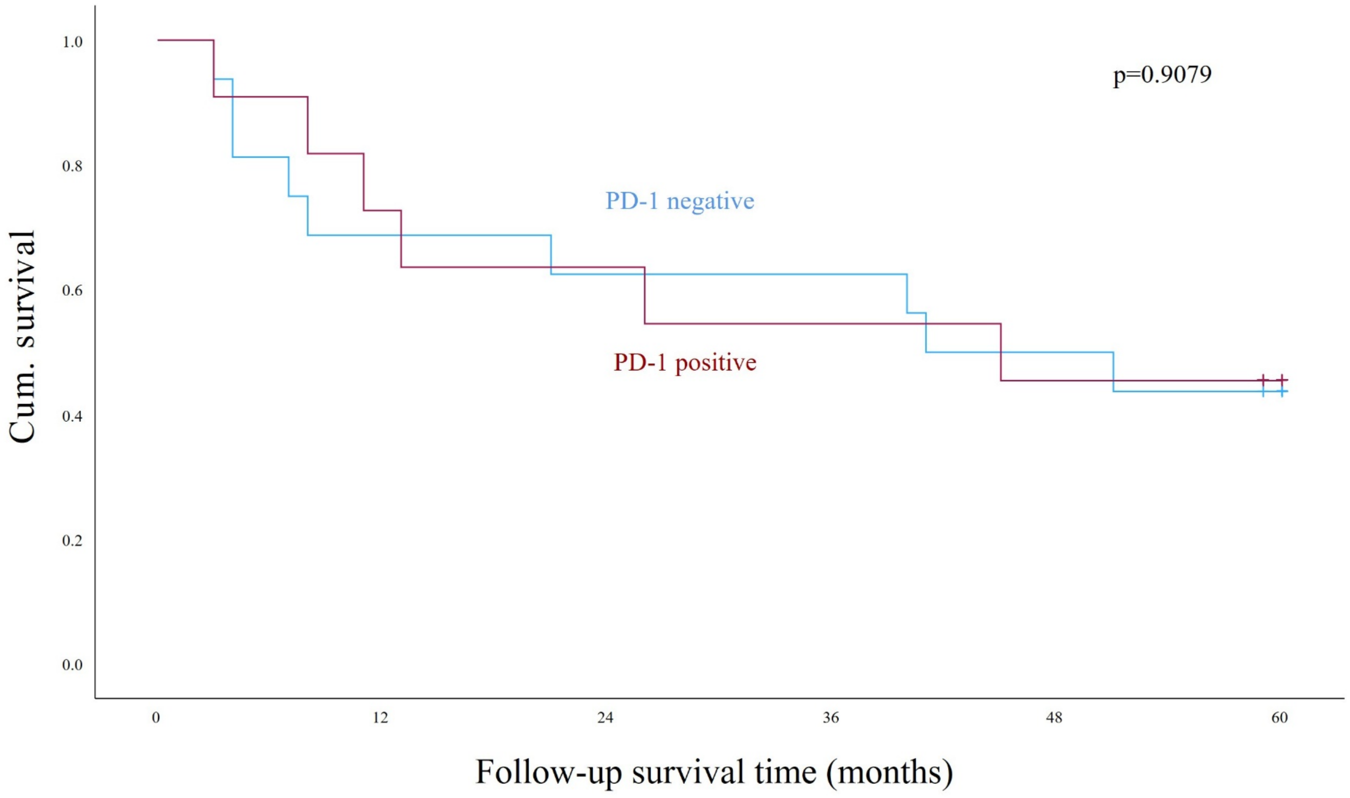

Figure S12: Kaplan-Meier analysis for PD-1 expression in subgroup "patients with surgery and radiation therapy"

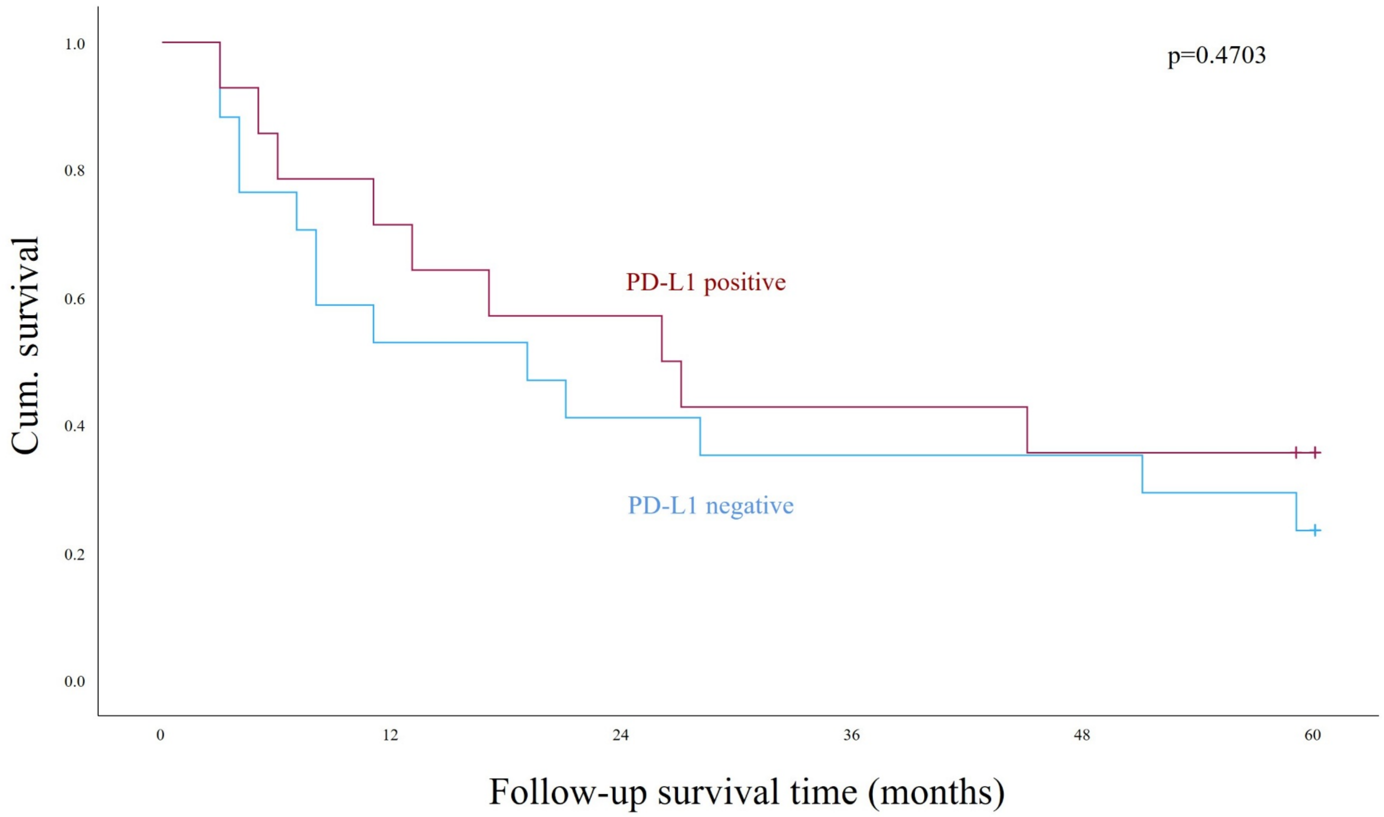

Figure S13: Kaplan-Meier analysis for PD-L1 expression in subgroup "patients with chemotherapy"

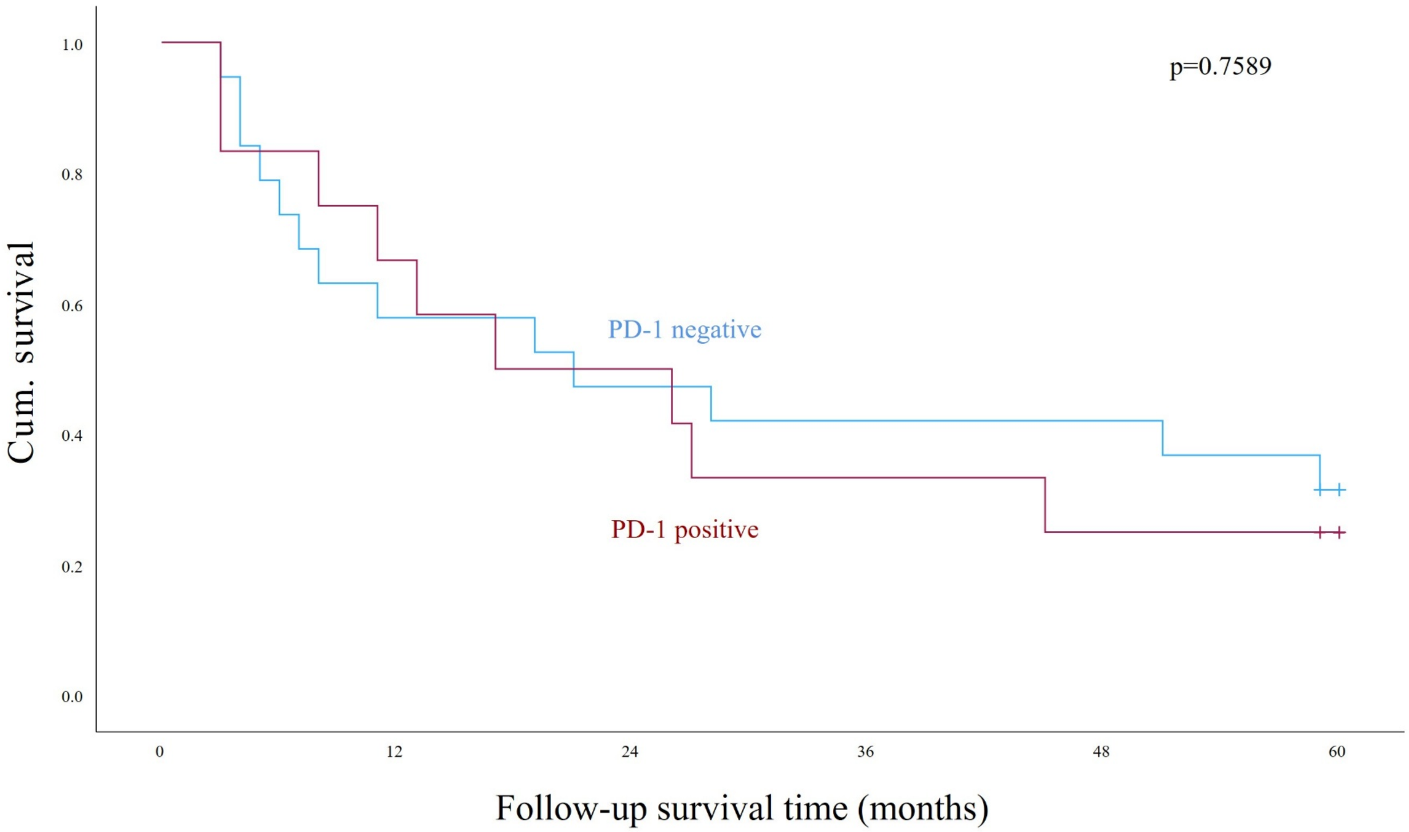

Figure S14: Kaplan-Meier analysis for PD-1 expression in subgroup "patients with chemotherapy"

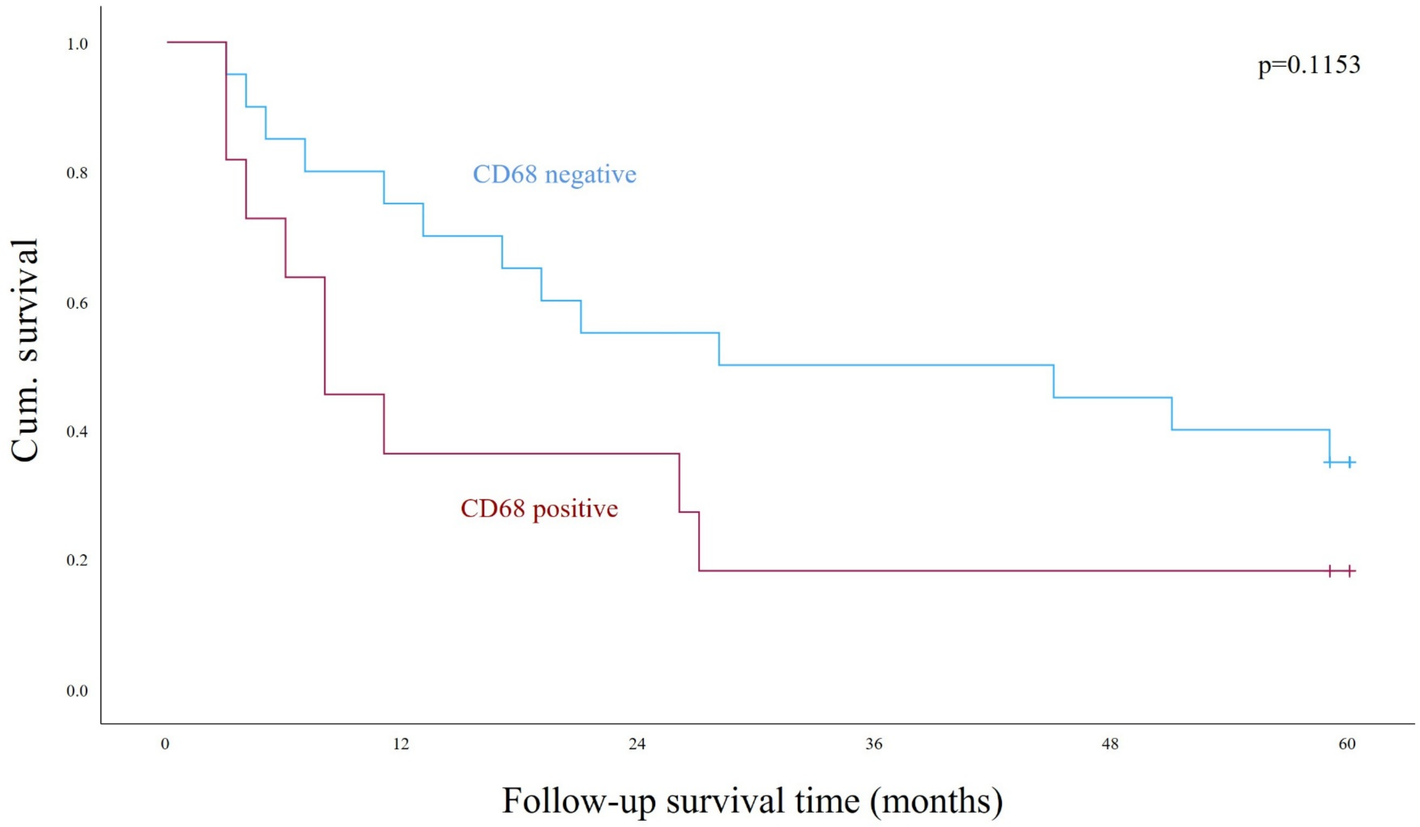

Figure S15: Kaplan-Meier analysis for CD68 expression in subgroup "patients with chemotherapy"

:

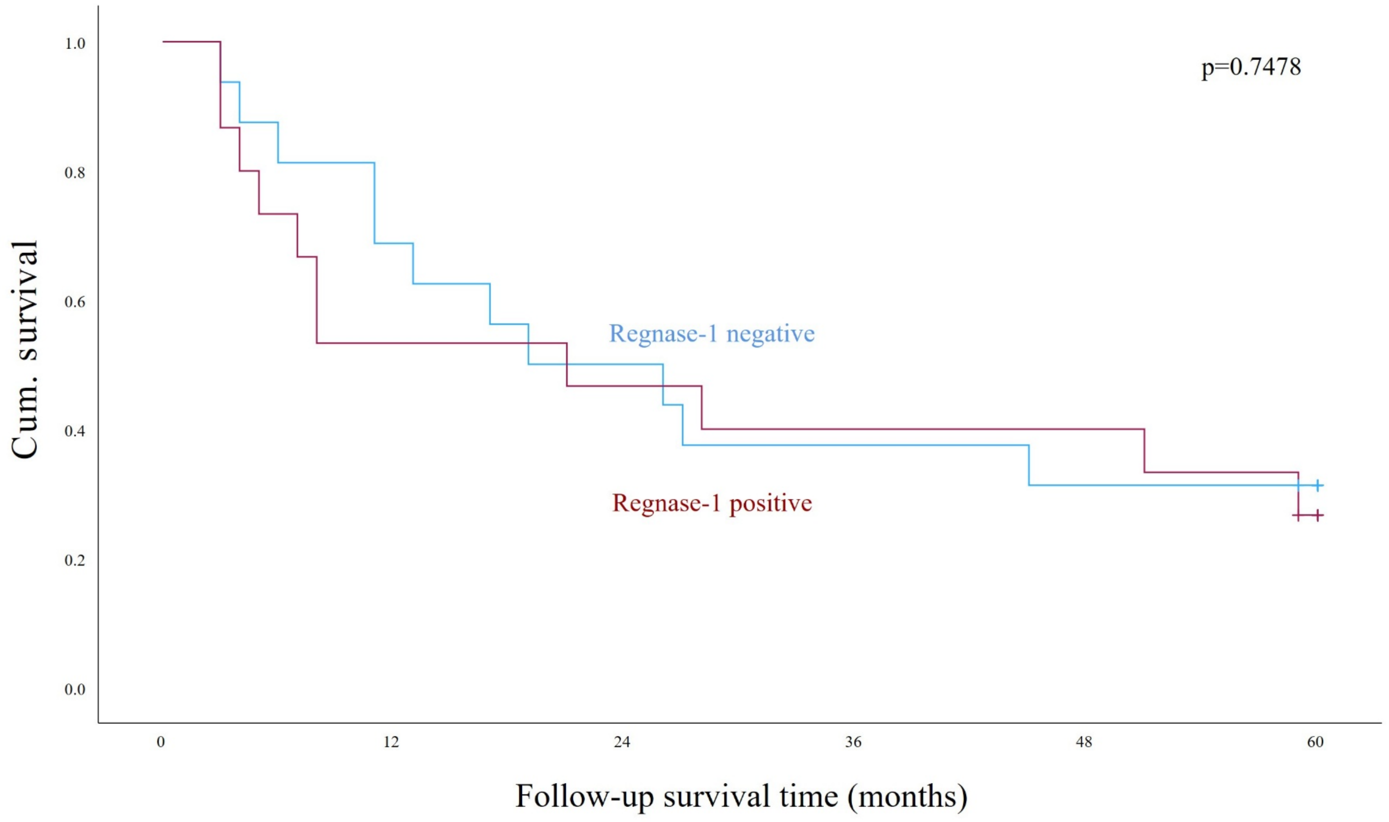

Figure S16: Kaplan-Meier analysis for Regnase-1 expression in subgroup "patients with chemotherapy"

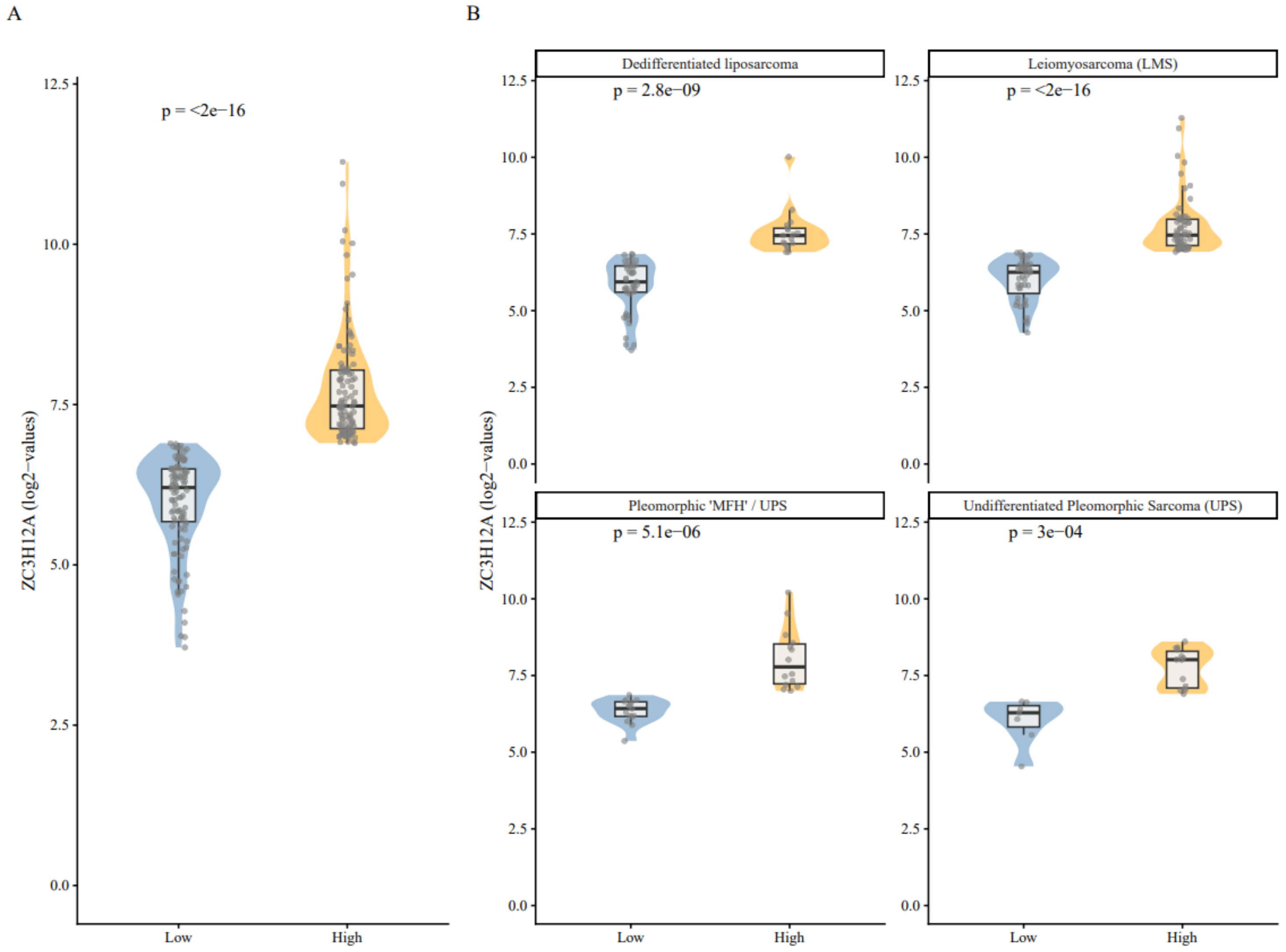

Figure S17A, and B. Median-based dichotomization of ZC3H12A/Regnase-1 expression levels

A

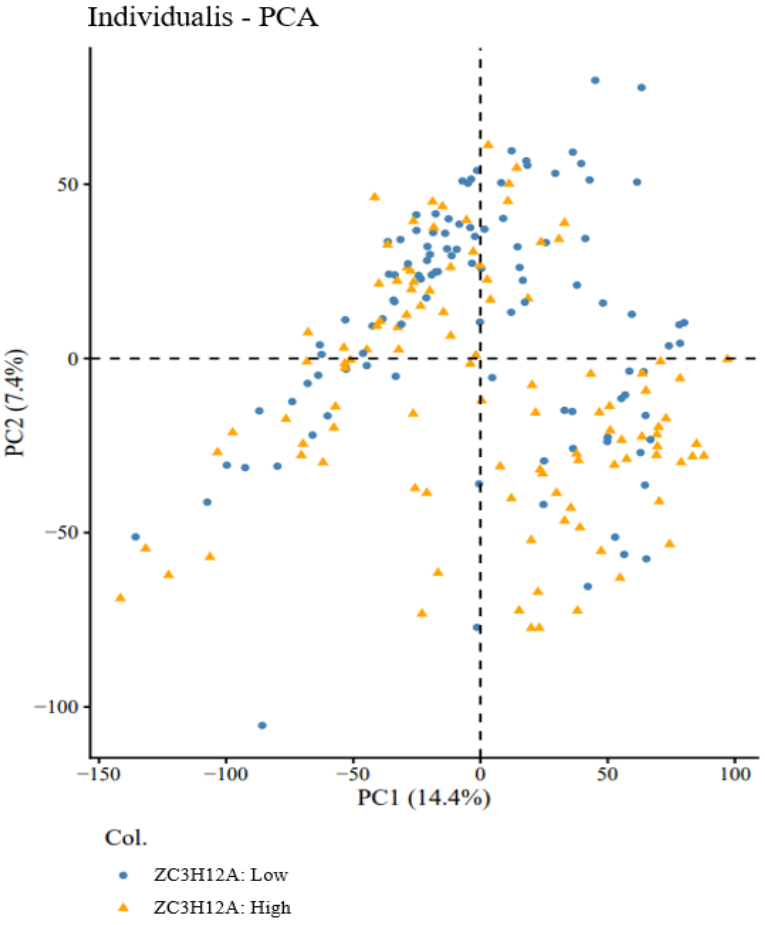
Individualis - PCA
PC2 (7.4%)
PC1 (14.4%)
50
0
−50
−100
−150
−100
−50
0
50
100
Col.
ZC3H12A: Low
ZC3H12A: High

B

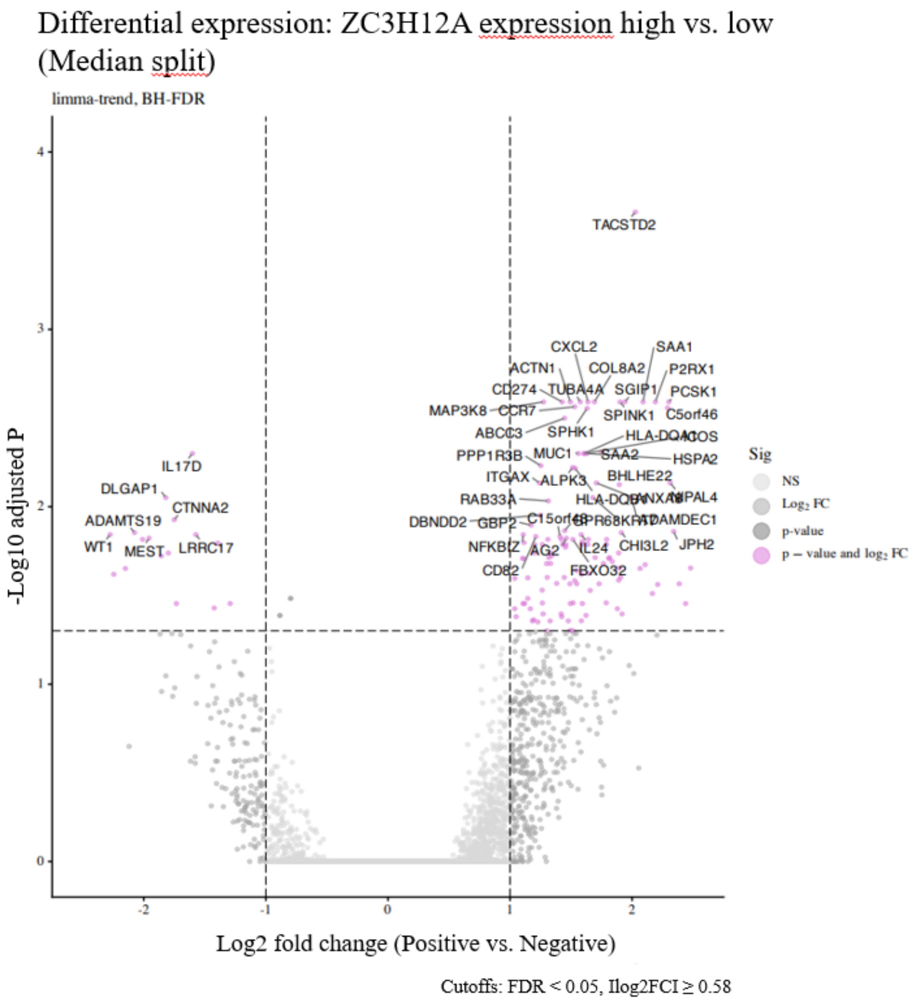

Figure S18A, and B. Gene expression analysis in *ZC3H12A*-positive STS using a median split model. (A) Sample distribution based on *ZC3H12A* expression. (B) Differential gene expression between *ZC3H12A*-positive and *ZC3H12A*-negative samples

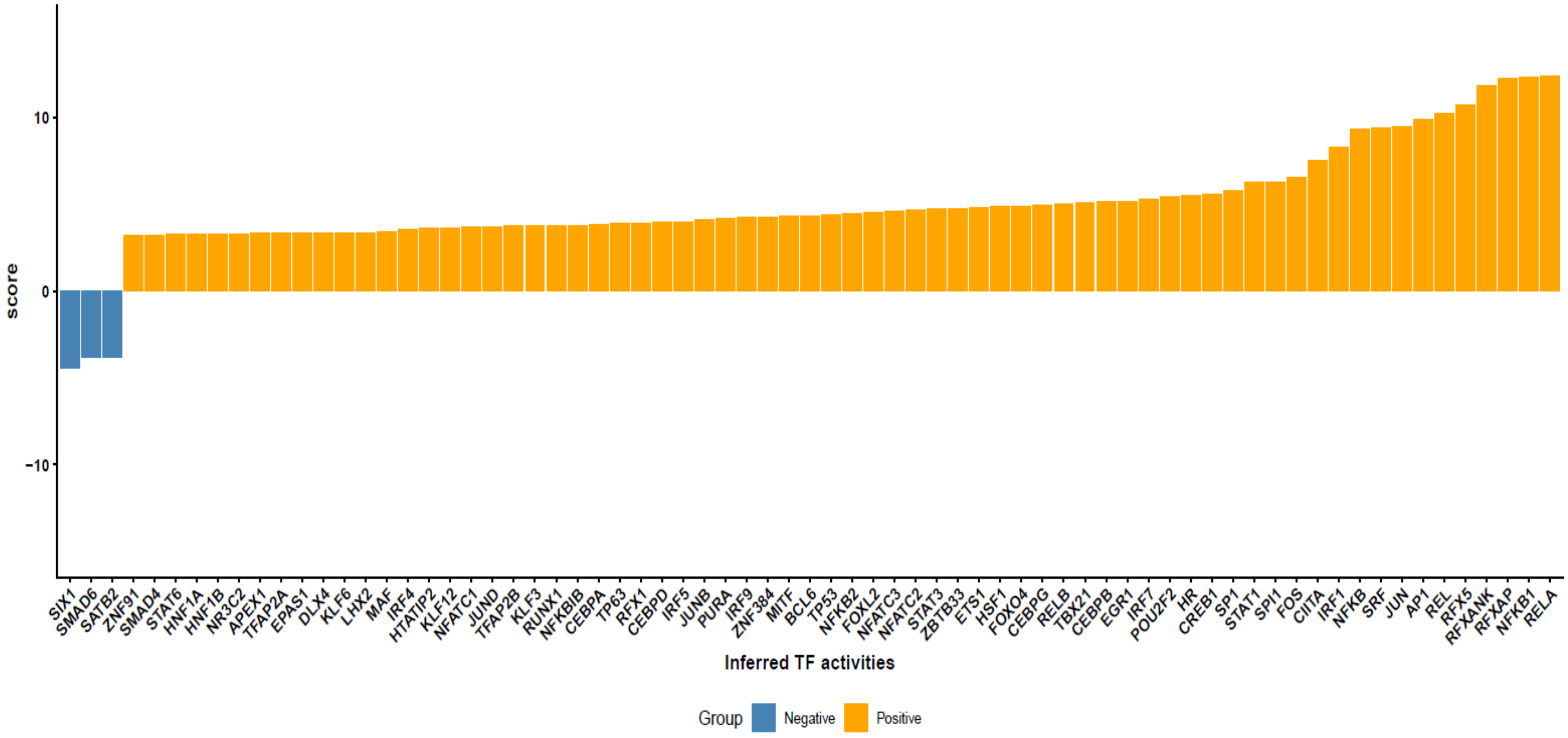

Figure S19. Analysis of transcription factor regulation in ZC3H12A-positive vs. ZC3H12A-negative STSs using median split model
